# Effectiveness of YCMAP (youth culturally adapted manual assisted problem solving) intervention in adolescents after self-harm in Pakistan: multicentre, randomised controlled trial

**DOI:** 10.1136/bmj-2024-083272

**Published:** 2025-09-12

**Authors:** Nusrat Husain, Sehrish Tofique, Tayyeba Kiran, Matthias Pierce, Imran B Chaudhry, M Omair Husain, Rakhshi Memon, Ahmed Waqas, Nadeem Gire, Sarah Edwards, Paul Bassitt, Suleman Shakoor, Zainab F Zaddeh, Usman Arshad, Erminia Colucci, Faraz Mughal, Samia Shahid, Maria Panagioti, Asad T Nizami, Farhat Jafri, Farooq Naeem, Richard Emsley, Moin A Ansari, Sarwat Sultan, Shilpa Aggarwal, Christopher Williams, Nasim Chaudhry

**Affiliations:** 1 Division of Psychology and Mental Health, The University of Manchester School of Health Sciences, Manchester, UK; 2 Global Centre for Research on Mental Health Inequalities, Mersey Care NHS Foundation Trust, Prescot, UK; 3 Division of Neuro-Cognitive Disorder, Older Adults Mental Health, Pakistan Institute of Living and Learning, Karachi, Pakistan; 4 Division of Mood Disorder, Pakistan Institute of Living and Learning, Karachi, Pakistan; 5 Dr Ziauddin Hospital, Karachi, Pakistan; 6 Campbell Family Mental Health Research Institute, Centre for Addiction and Mental Health, Toronto, Ontario, Canada; 7 Department of Psychiatry, Temerty Faculty of Medicine, University of Toronto, Toronto, Ontario, Canada; 8 Science and Technology Studies, University College London, London, UK; 9 Manchester Global Foundation, Manchester, UK; 10 Faculty of Health and Life Sciences, University of Liverpool, Liverpool UK; 11 Statsconsultancy, Amersham, UK; 12 Division of Biostatistics, Pakistan Institute of Living and Learning, Karachi, Pakistan; 13 Division of Child and Adolescents, Pakistan Institute of Living and Learning, Karachi, Pakistan; 14 Department of Evidence Synthesis, Pakistan Institute of Living and Learning, Karachi, Pakistan; 15 Department of Psychology, Middlesex University, London, UK; 16 Department of General Practice and Primary Care, Melbourne Medical School, University of Melbourne, Australia; 17 NIHR Greater Manchester Patient Safety Research Collaboration, University of Manchester, Manchester, UK; 18 Department of Applied Health Sciences, University of Birmingham, Birmingham, UK; 19 Division of Population Health, Health Services Research & Primary Care, Manchester, UK; 20 Institute of Psychiatry, Punjab, Pakistan; 21 Community Medicine, Karachi Medical and Dental College, Karachi, Pakistan; 22 Department of Psychiatry, University of Toronto, Toronto, Ontario, Canada; 23 Department of Biostatistics and Health Informatics, Institute of Psychiatry, Psychology and Neuroscience, King's College London, London, UK; 24 Department of Psychiatry, Sir Cowasjee Jehangir Institute, Hyderabad, Pakistan; 25 Department of Applied Psychology, Bahauddin Zakariya University, Multan, Pakistan; 26 IMPACT Strategic Research Centre, School of Medicine, Deakin University, Geelong, Australia; 27 Centre for Adolescent Health, Murdoch’s Children’s Research Institute, Melbourne, Australia; 28 School of Health & Wellbeing, University of Glasgow, Glasgow, UK; 29 Five Areas, Glasgow, UK; 30 Pakistan Institute of Living and Learning, Karachi, Pakistan

## Abstract

**Objective:**

To evaluate the clinical effectiveness of the YCMAP intervention (Youth Culturally Adapted Manual Assisted Problem Solving) for adolescents after self-harm in Pakistan.

**Design:**

Multicentre, randomised controlled trial that compared YCMAP with enhanced treatment as usual.

**Settings:**

General practices, emergency departments, medical wards of participating hospitals, and community centres across Karachi, Hyderabad, Lahore, Multan, and Rawalpindi.

**Participants:**

Adolescents with a recent history of self-harm identified at participating health centres by treating physicians between 5 November 2019 and 31 August 2021.

**Intervention:**

The YCMAP group received up to 10 treatment sessions over three months; the intervention was based on the principles of cognitive behaviour therapy.

**Main outcome measure:**

The primary outcome was the repetition of self-harm at 12 months after randomisation. Secondary outcomes were distress, hopelessness, suicidal ideation, and health related quality of life at three, six, nine, and 12 months after randomisation. Participants’ satisfaction with the services was assessed at three and 12 months after randomisation.

**Results:**

This trial was conducted between November 2019 and February 2023 and included 684 adolescents randomised to YCMAP (n=342) or enhanced treatment as usual (n=342). The YCMAP group had a significantly lower risk of self-harm repetition than the enhanced treatment as usual group at 12 months after randomisation (odds ratio 0.20, 95% confidence interval 0.06 to 0.70, P=0.006). YCMAP participants showed a statistically significant reduction in distress, hopelessness, and suicidal ideation at three months, but these differences were not statistically significant at 12 months. YCMAP participants also reported significantly better quality of life and satisfaction with services at three months, with these effects sustained at all follow-up points.

**Conclusion:**

The YCMAP intervention was shown to be beneficial in self-harm prevention among adolescents. Further research and replication of findings in diverse settings are recommended to strengthen the evidence base for this public health intervention.

**Trial registration:**

ClinicalTrials.gov NCT04131179 and ISRCTN registry ISRCTN57325925.

## Introduction

Suicide is a major global public health concern and is a leading cause of death among young people aged 15-29 years.^1^ In 2019, more than 700 000 deaths by suicide were recorded globally,^1^ with over two thirds of these deaths occurring in low and middle income countries.^1^ A history of self-harm is strongly associated with subsequent suicide,^2^ making it a critical focus for prevention efforts. Self-harm is particularly high among young people in South Asia,^3^ including Pakistan,^4^ where it leads to substantial treatment costs and has a major economic impact.^5^ Adolescents who have self-harmed are at future risk of repeat self-harm, mental illness, poor educational and employment outcomes, and suicide.^6^

Adolescent self-harm is a growing concern worldwide, with increasing recognition of the need for interventions that are evidence based and culturally sensitive. However, in low and middle income countries, there is a major gap in the availability and testing of such tailored interventions. Various risk factors for self-harm among young people in low and middle income countries have been identified, including family conflict, truancy, associations with peers who self-harm, and school absenteeism.^7^ In Pakistan, adolescents have reported interpersonal conflicts and emotional dysregulation as key predisposing factors for self-harm episodes.^8^ These young people have also emphasised the need for intervention programmes that offer emotional catharsis, promote healthy routines, and teach problem solving skills to enhance coping mechanisms.^8^

Adolescence is a critical developmental period characterised by emotional, social, and cognitive growth, during which people are particularly vulnerable to impulsivity, emotional dysregulation, peer influences, and heightened sensitivity to social rejection and external stressors.^7^ ^8^ Given the rising rates of self-harm in Pakistan (incidence rate per 100 000 population: 61.8, 95% confidence interval 37.67 to 92.09), it is critical we develop and test interventions to reduce self-harm recurrence.^9^ A meta-analysis on suicide prevention in young people highlighted that interventions delivered in educational and clinical settings are effective in reducing self-harm immediately after the intervention, but the effects diminish at follow-up.^10^ Typically, cognitive behavioural therapy (CBT) interventions for self-harm involve structured, one-to-one sessions delivered by trained clinicians, with a focus on cognitive restructuring, problem solving, emotion regulation techniques, and crisis planning.^11^ However, interventions might also involve families, be delivered in group settings, or use medium to low intensity approaches administered by non-specialist mental health workers.^11^ Increasingly, smartphone applications and text or call based services have been developed for adolescent self-harm.^12^ Considering the benefits of CBT based psychotherapies in reducing self-harm in adults,^13^ there is a strong case for exploring similar treatments for adolescents. Existing evidence underscores the need for targeted suicide prevention interventions for adolescents,^10^ taking into account the diverse cultural and socioeconomic contexts across different countries.^14^

A CBT based culturally adapted manualised intervention (CMAP) has already been evaluated in Pakistan with adults after self-harm, showing effectiveness in reducing depression (mean difference −7.1, 95% confidence interval −8.7 to −5.4), hopelessness (−2.6, −3.4 to −1.8), and suicidal ideation (−3.6, −4.9 to −2.4) at the end of the intervention (three months after randomisation).^15^ ^16^ Although the CMAP intervention also reduced the repetition of self-harm, the difference between the intervention and control groups was not statistically significant.^16^ However, the effectiveness of CMAP for young people in Pakistan has not yet been established. Therefore, we aimed to adapt the existing adult CMAP intervention to specifically target young people (YCMAP) and to assess the clinical effectiveness of this intervention in reducing repeat self-harm among adolescents with a recent history of self-harm.

## Methods

### Study design

This study was a two arm, multicentre, randomised controlled trial with an internal pilot (stop or go criteria),^17^ funded by the Medical Research Council, the Department for International Development, and a National Institute for Health and Care Research programme (MR/R022461/1). The trial was conducted across five major cities in Pakistan: Karachi, Hyderabad, Lahore, Multan, and Rawalpindi. These sites were selected to ensure geographical and cultural diversity in our sample, rather than because they are the only major hubs offering self-harm specific healthcare services. Each city has established healthcare facilities (including primary care centres, emergency departments, and medical wards) capable of providing interventions for self-harm, but these locations also serve a broader range of healthcare needs. Ethical approval was obtained from the National Bioethics Committee of Pakistan and the Research Ethics Committee of the University of Manchester before the enrolment of participants. The trial is registered with ClinicalTrials.gov (NCT04131179) and ISRCTN (ISRCTN57325925). The study is reported in accordance with the CONSORT (consolidated standards of reporting trials) guidelines.^18^ A detailed protocol of the study has been published elsewhere.^17^

### Participants

The study included adolescents aged 12-18 years who were identified by clinicians at the participating recruitment sites as having a recent history of self-harm (within the past three months). Eligible participants were residents of the trial catchment area and were already registered with clinical or health services to ensure they had access to routine care. Adolescents were excluded by trained assessors if they had a severe mental illness already diagnosed by a clinician (such as schizophrenia spectrum and other psychotic disorders, bipolar disorder, or severe depressive disorder), conditions that would limit their ability to engage with the assessment or intervention (such as an intellectual disability), or if they were temporary residents with a lower likelihood of being available for follow-up.

Self-harm was defined as “an act with non-fatal outcome, in which an individual deliberately initiates a non-habitual behaviour that, without interventions from others, will cause self-harm, or deliberately ingests a substance in excess of the prescribed or generally recognised therapeutic dosage, and which is aimed at realizing changes which the subject desired via the actual or expected physical consequences.”^19^ This was the criterion that we also used in the earlier study.^15^ ^16^

The sample size was based on the primary outcome: repetition of self-harm in a 12 month period (yes or no). The treatment as usual arm of the study was expected to have a self-harm rate of 20%.^20^ Theory of change sessions and in-depth consultations with multidisciplinary stakeholders including experts in mental health, emergency care, and medical specialists agreed that a reduction to 7.5% in the intervention group after treatment would be considered clinically meaningful. This reduction also aligns with an evidence synthesis exercise.^11^ Under these assumptions, and with a 5% significance level and 90% power, a study with no clustering would require 158 patients per group. The study has a partially nested design owing to therapist clustering in the Y-CMAP group. Our sample size calculation took this into account by adjusting the sample size upwards, conservatively assuming clustering in both groups. Based on a previous analysis of therapist trials, we expected that the intraclass correlation coefficient is likely to have a value between 0.01 and 0.05 for this type of outcome measure. Assuming an intraclass correlation coefficient of 0.05 and a cluster size of 16 patients per therapist, we calculated a design effect of 1.75, increasing the numbers required to 277 participants per group. Furthermore, with an expected loss to follow-up of 15%, the final numbers recruited were calculated as 326 per group, 652 in total.

### Randomisation and masking

Once eligibility was confirmed, and consent obtained, the research team notified the independent randomisation centre, which then assigned each adolescent a trial number. Recruited adolescents were randomly allocated in a 1:1 ratio to one of two trial arms: YCMAP plus enhanced treatment as usual, or enhanced treatment as usual alone. Treatment assignment was carried out using stochastic minimisation,^21^ controlling for age, sex at birth, and method of self-harm. Stochastic minimisation was used to ensure balanced allocation of participants across treatment groups with respect to key prognostic variables: age, sex, and method of self-harm. Stochastic minimisation is widely used in clinical trials when strict balance across groups is desired, while maintaining an element of randomness to prevent predictability in allocation.^22^ An independent statistician communicated the random assignments to the project manager through a password protected Excel sheet. Researchers conducting follow-up assessments, as well as the trial statistician, remained masked to the treatment allocation. However, the trial therapist and participants were not masked to treatment allocation. Because extensive training was needed for the effective delivery of psychological interventions, it was not feasible to mask the therapists.^23^

### Procedure

Potential participants were identified by the treating clinicians between 5 November 2019 and 31 August 2021 at participating general practices, emergency departments, medical wards of participating hospitals, and community centres in Karachi, Hyderabad, Lahore, Multan, and Rawalpindi. After an initial introduction by treating clinicians, eligible participants were approached by the research team, who explained the study in detail and obtained informed consent. A trained research team member provided detailed study information and the participant information leaflet in Urdu language to the potential participant and their parent or guardian. Eligible adolescents were then invited to participate. For those who were unable to read the leaflet (13% of the sample), a detailed description of the study was provided by the research team in the presence of a trusted community member identified by the parent or guardian. Those who met the criteria were invited to take part. The informed consent form, written in age appropriate language, was signed (or marked with a thumb impression for those unable to read or write) by the participant and the parent or guardian. For those unable to read or write, the trusted community member also signed the consent form. During the covid-19 outbreak, potential participants were approached by telephone, and verbal consent was audio recorded. These consent recordings were securely stored in password protected systems.

Baseline assessments were completed for all eligible participants who had given consent (face to face or by telephone or video call), and a unique study identification number was assigned to each participant by the project manager or site leads. These numbers, along with other relevant information, were sent to the independent statistician for randomisation. All participants were informed of their randomisation status within a week after the randomisation. Participants randomised to the intervention group were approached by the therapists (by telephone call) within two weeks of randomisation to begin intervention sessions. All participants, regardless of treatment allocation, completed assessments at three months (after intervention), six, nine, and 12 months after randomisation (face to face or through a telephone or video call). All participants were reimbursed for their time and travel. For participants who were unable to read or write, the self-report questionnaires were administered verbally by trained researchers. The researcher read each question aloud to the participant and recorded their responses, ensuring that the participant's answers were accurately captured. This approach maintained the integrity of the data while accommodating participants with literacy challenges. All assessments were conducted with careful consideration to ensure that all participants, regardless of their literacy level, were able to complete the scales in a consistent and reliable manner.

#### Covid-19 continuity plan

In March 2020, amendments to the initially agreed trial procedures were implemented because of restrictions (lockdown, social distancing) during the first wave of the covid-19 global pandemic. At this time, the trial was in the internal pilot phase, and to ensure the project continued smoothly, the following changes were made to recruitment, assessments, follow-up, and the delivery of the intervention: shift from face-to-face written (thumb impression) consent procedures to audio recorded verbal consent; transition of assessments and intervention delivery from face-to-face to remote procedures (telephone or video call).

### Intervention

The experimental intervention, Y-CMAP, consisted of psychoeducation and a structured cognitive behavioural approach^17^ specifically tailored for young people who have engaged in self-harm. This intervention^17^ is grounded in the principles of CBT and was adapted,^24^ with permission, from three existing intervention manuals: CMAP,^16^ “Life after self-harm,”^25^ and “Cutting down: A CBT workbook for treating young people who self-harm.”^26^

The intervention used virtual narratives featuring four relatable characters to enable cognitive behavioural assessment and therapeutic dialogues. Therapists used these narratives to guide participants in exploring and understanding their self-harm episodes, including identification of triggers, emotional experiences before and after the incidents, and family reactions. Participants collaboratively developed personalised crisis plans designed to mitigate the risk of future self-harm episodes. The intervention integrated problem solving strategies alongside CBT and dialectical behaviour therapy techniques, aiming to strengthen participants' ability to recognise and modify maladaptive thought patterns. Additional components included training in assertiveness, anger management, emotion regulation, and developing coping and problem solving skills to enhance resilience (box 1).

Box 1 **Description of YCMAP (youth culturally adapted manual assisted problem solving) intervention content**

**Session 1: Getting started—helping participating adolescents to make sense of their self-harm attempt**

Understanding reasons of self-harm, feelings after self-harm
Timeline of self-harm

**Session 2: What to do in a crisis**

Getting support
Keeping yourself safe
Activity scheduling
Crisis plan

**Session 3: Psychoeducation about cognitive behavioural therapy (CBT) with emphasis on helping adolescents to understand feelings and emotions**

Identifying problems and goals
What is CBT model
What are emotions
Feelings diary/record

**Session 4: Motivation to change**

Feedback on feeling diary
Motivation to change
Pros and cons of self-harm
Thinking about future

**Session 5: Negative automatic thoughts**

Understanding negative automatic thoughts
Thoughts distortions
Thought challenging
Thought recording

**Session 6: Core beliefs**

Identifying core beliefs
Case formulation

**Session 7: Coping strategies**

Coping tree
Problem solving

**Session 8: Assertiveness**

Asserting yourself
Assertiveness role play

**Session 9: Anger management**

Anger scale
Anger management techniques
Crisis first aid kit

**Session 10: Family session**

Understanding own feelings
Non-critical listening
Getting involved with life again
Professional help in crisis

In YCMAP, session 10 (“family session”) was an optional session involving family members, and was offered to all participants. This session aimed to increase participants' sense of self-worth by reinforcing their value within the family and broader social networks. The session guided family members to actively engage with participants, openly express support through crises, and encourage professional help-seeking when needed.

The YCMAP intervention was delivered by trained therapists holding a four-year degree in psychology, amounting to a total of 16 years of education, under the supervision of qualified professionals. Therapists received rigorous training refreshers and participated in regular supervision meetings conducted by CBT experts from the UK and Pakistan to ensure consistent implementation of the intervention. The intervention was delivered in 8-10 sessions over three months, in person or over a telephone or video call owing to covid-19 restrictions. The first eight sessions were offered weekly, followed by fortnightly sessions, with each session lasting up to 60 minutes on a one-to-one basis.

#### Manual refinements during the internal pilot

During the internal pilot, all trial therapists were requested to provide feedback on each session of the manual based on the following:

·What aspects of the session delivery were helpful and what needed improvement.
- Any challenges they encountered, including issues with word selection in the local language.
- The content of the sessions.
- General feedback from trial participants.

Two versions of the therapist manual and participant handouts were created—one in English and one in Urdu (box 1). Based on the feedback, certain words in the Urdu version were replaced by incorporating words with therapist suggested alternatives, additional notes were included to help therapists in explaining session activities, and extra examples were added to show various techniques, such as coping techniques, the ABC cycle, and recognising thinking distortions. Furthermore, completed worksheets (eg, coping tree, anger management) featuring hypothetical examples were incorporated to help therapists guide participants through the worksheets during the session. Owing to space constraints, qualitative insights from the participants’ and delivery agents’ feedback will be reported in a separate publication.

Regular consultation meetings were held with patient and public involvement and engagement groups to seek their advice on refining the session content, particularly to ensure that the content and language were accessible for the participants. The intervention manual is included as an online supplementary file.

### Enhanced treatment as usual

Enhanced treatment as usual allowed participants to continue receiving standard care routinely provided by local clinical and healthcare services, ensuring no constraints on usual care access owing to study participation. To systematically monitor and document the standard care provided, we used the client service receipt inventory, capturing the type and frequency of interventions received during the study period up to the 12 month follow-up.^27^ Moreover, all participants underwent comprehensive wellbeing assessments conducted by trained researchers at predetermined intervals. To adhere to the study’s safety protocols, participants from both arms received brief monthly check-in calls from researchers. 

### Training and supervision

All research staff were psychology graduates and were trained in good clinical practice.^28^ A two day training workshop at each recruitment site was led by the research team (NC, TK, ST) to provide orientation to the study, and use of assessment questionnaires. The training involved role play sessions and inter-rater reliability of scoring. This initial training was followed by the monthly training refresher sessions and a monthly whole group meeting where researchers shared their experiences with each other, including any challenges during assessments—remote assessments in particular.

For the intervention, all therapists received regular fortnightly supervision from international CBT experts (CW, FN) over a Zoom call and local experts (ZFZ, TK), either face to face or over a Zoom call. These meetings involved case discussions and strategies for identifying and managing distress in participants. Distress and risk assessment protocols adapted from earlier suicide prevention trials^15^ ^16^ were in place. These included details on managing difficult situations arising in research, participant and researcher safety, and lone working arrangements.

### Outcome measures

All assessments were conducted by masked assessors to ensure consistency and minimise bias. For self-reported measures, adolescents completed the questionnaires with the support of a researcher.

The primary outcome was the repetition of self-harm at 12 months after randomisation, assessed using the adapted suicide attempt self-injury interview.^29^ This interview^29^ is a semi-structured instrument designed to comprehensively evaluate factors associated with non-fatal suicide attempts and intentional self-injury. The instrument comprises five key domains: frequency, methods, intent and degree of self-injury, contextual factors, and functions of self-harm. The first domain assesses frequency of suicide attempts by directly recording the number of self-harm events occurring within the past year.

Secondary outcomes included levels of distress, feelings of hopelessness, suicidal ideation, health related quality of life and participants’ satisfaction with services. The following measures were used:

- Kessler psychological distress scale^30^—consists of 10 items, usually taking about two or three minutes to complete; scores range from 10 to 50, with higher scores indicating greater psychological distress.
- Beck hopelessness scale^31^—a 20 item true or false measure requiring around five minutes, yielding scores between 0 and 20, where higher scores signal greater hopelessness.
- Beck scale for suicidal ideation^32^—includes 19 items and also takes around five minutes to administer; scores range from 0 to 38, with higher scores reflecting greater suicidal ideation.
- EQ-5D-Y^33^—comprises five core questions plus a 0-100 visual analogue scale, typically completed in about two minutes; it generates an index value (usually from below 0 to 1) and a visual analogue scale score (0-100), with higher values indicating better overall health.
- Client satisfaction questionnaire 8^34^—contains eight items and takes approximately two minutes to complete; it produces scores between 8 and 32, with higher scores denoting greater satisfaction with services.
- Safety monitoring was conducted using a trial specific adverse event record form to document any adverse or serious adverse events during the trial.

All measures were translated using the approach described by Rahman and colleagues^35^ and had been used in previous suicide prevention trials in Pakistan.^15^ ^16^ Assessments were completed at baseline, at three months (after intervention), six, nine, and 12 months after randomisation. Additionally, in-depth qualitative interviews were conducted with participants from the intervention group at the end of the intervention, and with trial therapists (results reported separately).

### Statistical analysis

Statistical analyses were based on intention-to-treat principles. During the trial, periodic random quality checks of data were carried out by the trial statistician who was masked to treatment allocation. When data entry was completed, preliminary data analysis was carried out with treatment allocation masked. Baseline and follow-up data were summarised using the descriptive statistics.

Upon receiving the final dataset, several deviations from the original statistical analysis plan were necessary. Firstly, one of the patients in the YCMAP group died by suicide; therefore, it was decided to group this patient with the repetition of self-harm patients in the analysis of the primary outcome. Secondly, only 18 patients (three in one trial group) showed the primary outcome (repetition of self-harm). Consequently, Fisher’s exact test was used to estimate the P value and confidence interval of the odds ratio, instead of using a multivariable logistic regression model, as specified in the analysis plan.

Finally, 10 patients (1.5%) were lost to follow-up by 12 months (eight in the YCMAP group and two in the control group). Analyses found that loss to follow-up was associated with baseline age and self-harm variables (supplementary table 1). Therefore, a sensitivity analysis was conducted to assess potential bias in the primary results because of missing data, assuming data were missing at random. The primary outcome was reanalysed after applying stabilised weights, calculated from a logistic regression model to account for the missing data. Because Fisher's exact test cannot accommodate weights, odds ratios were calculated using weighted logistic regression models, and therefore the standard error was non-conservative. To assess whether the results were robust to the most extreme form of missing not at random, a second sensitivity analysis was conducted, assuming that all those who were censored experienced self-harm.

Continuous secondary outcome measures were analysed according to the initial plan, using linear mixed models. A single model was fitted across all time points that included a fixed effect for time, and interactions between time and treatment group. Two random intercepts were included at the participant level to account for the longitudinal nature of the data, and at the therapist level for those in the intervention group, to account for clustering by therapist. Based on Roberts and Roberts^36^ random intercepts were also included for each participant (ie, clusters of size one) in the control group. Additional fixed effects were included for baseline values of the outcome, age, sex, and type of self-harm as fixed effects.

### Patient and public involvement

The patient and public involvement and engagement group was established before the start of the trial. During the protocol development stage, we formed two patient and public involvement and engagement groups: one consisting of male and female adolescents (n=8) and another comprising care givers and parents (n=12). The groups played a crucial part in selecting primary and secondary outcomes, developing recruitment and retention strategies for trial participants, and contributing to study documents such as participant information leaflets, study advertisements, and interview schedules for process evaluation. Regular consultations were held with the groups during the refinement of the intervention. These groups made important contributions to the translation of the manual and suicide prevention awareness materials into local languages. Additionally, two members were part of the trial steering committee and attended all meetings. The adolescent group also helped to develop lay summaries of the findings.

The integrity of the data was continually monitored by the research team under the supervision of the chief investigator (NH). Additionally, site audits were conducted across all five study locations to ensure the accuracy and consistency of the data. Throughout the trial, participants remained in regular contact with the research team (site leads) to confirm they were not involved in any other concurrent trial.

## Results

**Fig 1:**
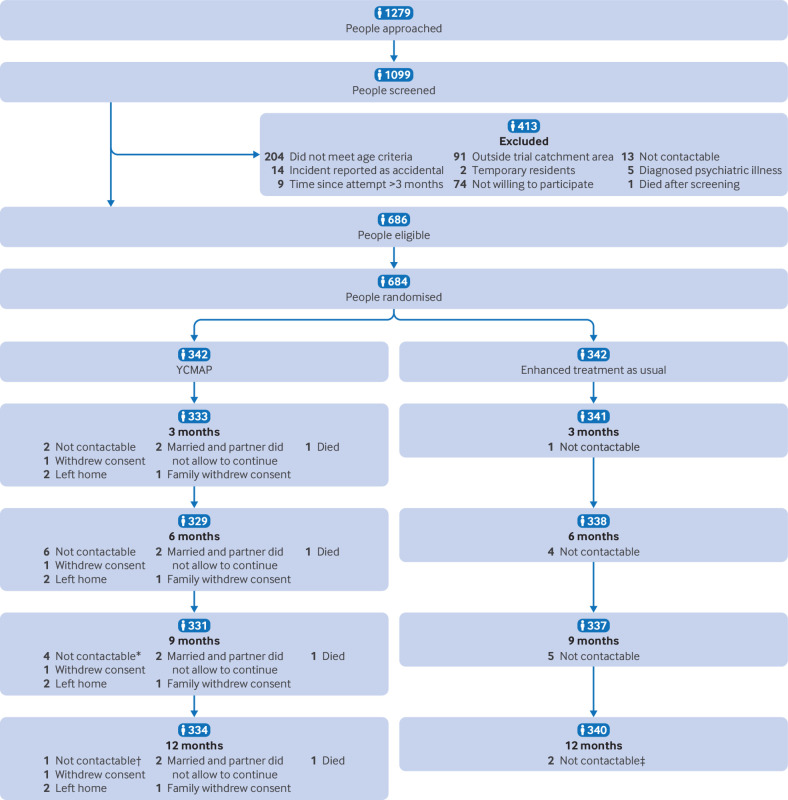
CONSORT (consolidated standards of reporting trials) flow diagram. YCMAP=youth culturally adapted manual assisted problem solving. *Two participants reconnected at nine months in YCMAP group. †Three participants reconnected at 12 months in YCMAP group. ‡Three participants reconnected at 12 months in enhanced treatment as usual group

A total of 1279 adolescents were approached through treating clinicians at recruitment sites, of whom 1099 were screened, and 684 met the eligibility criteria. These participants completed baseline assessments and were randomly assigned to the two study arms between 5 November 2019 and 31 August 2021 (fig 1). Table 1 summarises the demographic and clinical characteristics of the sample. Of the 684 participants, 365 (53%) were female and 319 (47%) were male, with a mean age of 16.1 years (standard deviation 1.7). Most participants (660, 96%) were single, while 22 (3%) were married, and two (1%) were separated or divorced. Most participants came from families who owned their homes (64%); 401 (59%) had received more than eight years of schooling, and 591 (86%) were unemployed. In the previous three months, 561 participants (82%) reported consulting a doctor.

**Table 1:** Demographic and clinical characteristics of participants at baseline

Characteristics	YCMAP plus enhanced treatment as usual (n=342)	Enhanced treatment as usual (n=342)	Total (n=684)
Age, mean (SD)	16.1 (1.7)	16.2 (1.6)	16.1 (1.7)
Total income (rupees), median (IQR)	20 000 (14 500-30 000)	20 000 (15 000-30 000)	20 000 (15 000-30 000)
Highest qualification at home, median (IQR)	10 (7-12)	10 (6-12)	10 (6-12)
**Sex**			
Male	160 (47)	159 (46)	319 (47)
Female	182 (53)	183 (54)	365 (53)
**Marital status**			
Single	332 (97)	328 (96)	660 (96)
Married	8 (2)	14 (4)	22 (3)
Separated or divorced	2 (1)	—	2 (1)
**Status of home**			
Own	223 (65)	216 (63)	439 (64)
Rent	119 (35)	126 (37)	245 (36)
**Education**			
No formal education	44 (13)	48 (1)	92 (13)
Middle or secondary (8 years)	95 (28)	87 (25)	182 (27)
Matric (10 years)	74 (22)	87 (25)	161 (23)
Intermediate (12 years)	25 (7)	24 (7)	49 (7)
Other (diploma)	3 (1)	6 (2)	9 (1)
**Employment**			
No	298 (87)	293 (86)	591 (86)
Yes	44 (13)	49 (14)	93 (14)
**Consulted any doctor during past three months**			
No	68 (20)	57 (17)	123 (18)
Yes	274 (80)	285 (83)	561 (82)
**Intent to die (suicide attempt self-injury interview)**			
Obviously no intent or minimal	46 (13)	38 (11)	84 (12)
Definite intent but very ambivalent	91 (27)	96 (28)	187 (27)
Serious intent or extreme intent	205 (60)	208 (61)	413 (61)
Communicated about self-harm*			
No communication	269 (79)	274 (80)	543 (79)
Indirect communication	09 (2)	12 (4)	21 (3)
Direct communication	64 (19)	56 (16)	120 (18)
**Method of self-harm**			
Ingestion of medication	143 (42)	135 (39)	278 (41)
Pesticide	83 (24)	91 (27)	174 (25)
Ingestion of toxic chemicals	84 (25)	78 (23)	162 (24)
Others (cuts, gunshot, and jumping from heights)	32 (9)	38 (11)	70 (10)

1000 rupees=£2.60; $3.50; €3.00.

Data are numbers (%) unless stated otherwise.

IQR=interquartile range; SD=standard deviation; YCMAP=youth culturally adapted manual assisted problem solving.

* Assessed using suicide attempt self-injury interview.

Most participants who self-harmed (n=600, 88%) reported doing so with the intent to die, with male and female participants expressing serious suicidal intent (n=280, 88% and n=320, 88%, respectively). Most participants (n=543, 79%) did not communicate their thoughts or plans of self-harm to anyone. The most commonly reported method of self-harm was the ingestion of medication (n=278, 41%), followed by the use of pesticides (n=174, 25%; table 1).

A total of 672 participants (98%) completed assessments at 12 months after randomisation. Analysis of the primary outcome revealed that there were only two self-harm repetitions (1%) in the intervention group at 12 months, along with one suicide, compared with 15 self-harm repetitions (4%) in the enhanced treatment as usual group (table 2).

**Table 2:** Odds of repetition of self-harm at 12 month follow-up by treatment group

Treatment group	No repetition	Repetition	Odds ratio (95% CI)	P value
Enhanced treatment as usual (reference)	325 (96%)	15 (4%)	Reference	—
YCMAP plus enhanced treatment as usual	331 (99%)	3 (1%)*	0.20 (0.06 to 0.70)	0.006

YCMAP=youth culturally adapted manual assisted problem solving.

* Includes one suicide in intervention group.

Table 3 compares clinical variables between the YCMAP plus enhanced treatment as usual and enhanced treatment as usual alone groups using independent sample t tests. Participants in the intervention group showed significantly lower scores on the Beck scale for suicidal ideation, the Beck hopelessness scale, and the Kessler psychological distress scale at three months after randomisation compared with those in the enhanced treatment as usual group; however, these differences were not sustained at subsequent follow-ups. Additionally, participants in the intervention arm had significantly higher EQ-5D scores at three months than the enhanced treatment as usual group, and the EQ-5D visual analogue scale scores remained significantly better at each follow-up time point.

**Table 3:** Analysis of secondary outcomes according to YCMAP plus enhanced treatment as usual or treatment as usual alone

Outcome and time	YCMAP plus enhanced treatment as usual	Enhanced treatment as usual	Difference*	P value
**Suicidal ideation—Beck scale for suicidal ideation†**				
Baseline	8.15 (8.95)	8.43 (9.27)	—	—
3 months	0.37 (2.25)	1.69 (4.98)	−0.86 (−1.65 to −0.07)	0.03
6 months	0.68 (2.97)	1.07 (4.12)	0.05 (−0.75 to 0.84)	0.91
9 months	0.22 (1.64)	0.49 (2.47)	0.18 (−0.61 to 0.97)	0.66
12 months	0.21 (1.71)	0.39 (2.23)	0.28 (−0.51 to 1.07)	0.49
**Hopelessness—Beck hopelessness scale‡**				
Baseline	11.15 (4.93)	11.27 (4.64)	—	—
3 months	5.40 (4.34)	7.26 (5.23)	−1.21 (−2.09 to −0.33)	0.007
6 months	7.88 (4.94)	8.33 (5.05)	0.21 (−0.67 to 1.10)	0.64
9 months	5.73 (5.04)	6.10 (5.18)	0.30 (−0.58 to 1.19)	0.50
12 months	5.23 (4.27)	5.71 (4.37)	0.18 (−0.70 to 1.06)	0.69
**Distress—Kessler psychological distress scale§**				
Baseline	28.13 (8.85)	29.05 (8.94)	—	—
3 months	15.28 (6.59)	19.48 (7.37)	−3.83 (−5.10 to −2.56)	<0.001
6 months	14.95 (6.82)	16.37 (7.57)	−1.06 (−2.34 to 0.21)	0.10
9 months	14.05 (6.10)	15.18 (6.58)	−0.78 (−2.06 to 0.49)	0.23
12 months	12.89 (5.28)	13.47 (5.11)	−0.21 (−1.48 to 1.06)	0.75
**Health related quality of life—EQ-5D§¶**				
Baseline	0.53 (0.36)	0.53 (0.34)	—	—
3 months	0.90 (0.20)	0.80 (0.27)	0.09 (0.04 to 0.14)	<0.001
6 months	0.90 (0.20)	0.86 (0.23)	0.02 (−0.03 to 0.07)	0.36
9 months	0.92 (0.16)	0.88 (0.21)	0.03 (−0.02 to 0.08)	0.21
12 months	0.94 (0.16)	0.91 (0.18)	0.01 (−0.03 to 0.06)	0.58
Health status—EQ-5D visual analogue scale**				
Baseline	57.06 (19.47)	57.59 (19.82)	—	—
3 months	79.64 (13.97)	72.21 (18.08)	8.59 (5.39 to 11.79)	<0.001
6 months	77.90 (14.89)	74.22 (16.58)	4.88 (1.66 to 8.09)	0.003
9 months	78.47 (13.71)	76.01 (14.15)	3.61 (0.40 to 6.82)	0.03
12 months	81.61 (12.41)	78.94 (13.04)	3.80 (0.60 to 7.00)	0.02

Summary statistics are mean (standard deviation) in each group. Mean difference (95% confidence interval) between groups also reported.

YCMAP=youth culturally adapted manual assisted problem solving.

* Difference reported as adjusted difference in mean outcome for YCMAP plus enhanced treatment as usual minus mean outcome for enhanced treatment as usual alone.

† Scores range from 0 to 38; higher scores indicate greater suicidal ideation.

‡ Scores range from 0 to 20; higher scores indicate greater hopelessness.

§ Scores range from 10 to 50; higher scores indicate greater psychological distress.

¶ EuroQol five dimensional youth questionnaire (EQ-5D-Y); index scores typically range from below 0 to 1; higher scores indicate better health related quality of life.

** Scores range from 0 to 100; higher scores indicate better self-reported health status.

To assess the robustness of the primary outcome to missing data, we conducted two sensitivity analyses. Under the assumption that data were missing at random, the significant intervention effect persisted (supplementary table 2). However, when assuming the most conservative scenario (ie, all participants lost to follow-up experienced self-harm), the between group difference was no longer statistically significant (supplementary table 3).

### Healthcare services sought by enhanced treatment as usual participants

Supplementary table 4 presents the pattern of healthcare service use among trial participants in the YCMAP and enhanced treatment as usual groups. Participants in the enhanced treatment as usual group reported a higher number of outpatient clinic visits for physical health concerns (902 visits *v* 163 among YCMAP recipients) and more consultations with general practitioners (376 *v* 64). Additionally, enhanced treatment as usual participants sought help from faith healers more frequently (141 *v* 92 sessions in the YCMAP group). Psychological healthcare use was minimal across both groups. The mean number of sessions attended by the YCMAP participants was 8.67 (*v* 1.36 for enhanced treatment as usual; supplementary table 4).

### Adverse and serious adverse events

In addition to the suicide in the intervention group, there were two further serious adverse events—one involving a diagnosis of covid-19 and the other substance misuse; both occurring in the intervention group. However, neither of these events was deemed by the study's medical assessors to be attributable to the intervention. A total of 11 adverse events were reported during the study: 10 in the enhanced treatment as usual group (fever=4, seasonal allergy=1, road accident=2, flu=2, high blood pressure=1) and one in the intervention arm (flu).

## Discussion

### Principal findings

This randomised controlled trial from a low or lower middle income country evaluated a manual assisted suicide prevention intervention that involved 684 adolescents aged 12-18 years who had self-harmed. The YCMAP intervention significantly reduced the repetition of self-harm compared with the enhanced treatment as usual group (1% *v* 4%, P=0.007). Participants in the intervention group also showed greater reductions in distress, hopelessness, and suicidal ideation, along with improved health related quality of life and higher satisfaction with services at three month and 12 month follow-ups. This study benefits from a rigorous randomised controlled design, ensuring robust data, and its long term follow-up and high retention rate strengthen the reliability of the findings. The active involvement of patient and public groups added valuable perspectives, making the intervention more relevant and effective. However, the absence of masking for therapists and participants, the adjustments required because of covid-19, and the relatively few outcome events present challenges that suggest areas for further exploration.

### Comparison with other studies

The reduction in self-harm repetition is particularly important given that young people who self-harm face a suicide risk more than 30 times higher than the general population.^37^ These findings contrast with previous reviews,^10^ ^38^ which found limited evidence supporting the effectiveness of clinical interventions in reducing self-harm and suicidality. A similar trend was observed in a randomised controlled trial of a CBT based CMAP intervention in Pakistan for adults after self-harm, which improved depression, hopelessness, and suicidal ideation, but not self-harm repetition.^16^ Additionally, a meta-analysis of psychosocial interventions for adolescents highlighted that, although these interventions reduced suicidal ideation, they did not significantly affect self-harm behaviour.^39^

The findings of this multicentre randomised controlled trial align with a practitioners' review^40^ highlighting growing evidence for the effectiveness of psychological interventions, particularly dialectical behaviour therapy adapted for adolescents, in reducing self-harm. However, this treatment has primarily been evaluated in adolescents with borderline personality disorder traits, with trials involving relatively small sample sizes (77^41^ and 173^42^ participants) and limited evidence of sustained long term effects.^41^ Given that dialectical behaviour therapy adapted for adolescents is an intensive intervention designed for people with severe and recurrent self-harm, its accessibility and scalability in low resource settings remain a challenge. YCMAP, a structured problem solving intervention, offers a brief and culturally adapted alternative that considers the ongoing risk of self-harm throughout the 12 months after enrolment into the trial.^43^ Although not a direct substitute for dialectical behaviour therapy adapted for adolescents, YCMAP provides a scalable option for broader adolescent populations, particularly where access to intensive treatments is limited.

In the current trial, scores on all three clinical outcomes—distress, hopelessness, and suicidal ideation—decreased for participants in both trial groups. However, after intervention, the reduction in these outcomes was significantly greater for participants in the intervention group than the control group. However, the difference between the groups at long term follow-up (12 months after randomisation) was not statistically significant. This finding aligns with a trial comparing dialectical behaviour therapy with routine care, which also reported reduced scores for both groups, but no long term between group difference in suicidal ideation, hopelessness, and depression.^41^ The YCMAP intervention also led to significant health related quality of life improvements, a key healthcare indicator and a predictor of suicide risk.^44^ Additionally, higher satisfaction with services among intervention group participants underscores the importance of these findings for health policy decision makers.^45^

### Policy implications

Although the overall self-harm rate remained relatively low in both groups, several considerations highlight the clinical importance of our findings. At the three month follow-up, participants in the YCMAP group showed markedly faster and greater reductions in psychological distress, hopelessness, and suicidal ideation than those receiving enhanced usual care. Although the enhanced usual care eventually caught up in later assessments, this early advantage is clinically meaningful because a quicker recovery might help mitigate prolonged distress and its potential long term consequences. Furthermore, even small reductions in self-harm carry substantial value in high risk populations when viewed through absolute and relative risk differences. Qualitative feedback from adolescents and therapists highlighted the real world acceptability, feasibility, and potential impact of YCMAP, while preliminary economic analyses further suggest the cost effectiveness of scaling this approach in existing healthcare systems in Pakistan and similar contexts.

To contextualise these observations, we note that a meta-analytical database compiled by van Ballegooijen and colleagues included 29 studies examining various psychosocial interventions for suicide prevention among children and adolescents.^46^ Although the pooled effect size (Hedges’ g=0.07, 95% confidence interval −0.08 to 0.22, P=0.360) was small and did not reach statistical significance, there was notable variation across intervention types—most prominently in dialectical therapy (g=0.361, P=0.021). The number needed to treat (56.4) in this meta-analysis underscored the difficulty of achieving large scale reductions in self-harm behaviours. Our findings align with recent individual participant data meta-analytic evidence indicating limited overall effectiveness of interventions compared with controls in preventing repeat self-harm at 12 month follow-up (odds ratio 1.06, 95% confidence interval 0.86 to 1.31), but highlight that interventions might yield greater benefits among adolescents with several self-harm episodes.^11^

However, in line with our findings, even modest effects can be clinically consequential, especially when linked to improved engagement, acceptability, and cost effectiveness. Future research should therefore concentrate on optimising specific intervention components, identifying subgroups most likely to benefit, and considering multifaceted outcomes—including broader familial and social factors—that can further elucidate the real world impact of self-harm interventions for adolescents. The recent individual participant data meta-analysis similarly highlights the critical need for funders and researchers to agree on a core set of outcome measures, highlighting the importance of consistent reporting to enable robust comparisons and improve the effectiveness of self-harm interventions.^11^

This study addresses a critical evidence gap in suicide prevention interventions for adolescents, particularly in low and middle income countries, where there is limited research on long term outcomes.

### Strengths and limitations of this study

The trial has several strengths, including the use of robust trial methodology, a prospective and long term follow-up design, and rigorous procedures for data collection across several sites. The study also benefited from a strong governance structure (trial steering committee, data monitoring, and ethics committee) and systems for building the capacity and capability of the research team throughout the trial, including inter-rater reliability sessions to ensure the high integrity of ratings. Moreover, the study achieved a high retention rate at 12 month follow-up.

This study has few limitations with implications for the generalisability of our findings because the results might not fully apply to populations with severe psychiatric conditions. Patients with severe mental illnesses, such as schizophrenia spectrum disorders, other psychotic disorders, bipolar disorder, and severe depressive disorders, were intentionally excluded from this study because the intervention was specifically developed to address self-harm among people experiencing common mental health conditions. The exclusion aimed to maintain a homogeneous study population and ensure the intervention was tailored effectively for the targeted group. The exclusion of people with severe mental illnesses and borderline personality disorder could partly explain the lower than anticipated event rate of self-harm observed in the trial because these conditions are often associated with a higher risk of self-harm behaviours. Future research is necessary to explore adaptations of this intervention suitable for people with severe mental illnesses, which would help to clarify its broader clinical applicability and effectiveness across diverse patient groups. Lastly, our research was conducted within a context where legislation, informed by Islamic religious beliefs, historically criminalised suicidal behaviours.^47^ Such legislation might contribute to underreporting of self-harm incidents, reduced help seeking behaviours, and potentially lower engagement with mental health interventions, affecting the generalisability and interpretation of findings. Because of the nature of the study, participant masking was not feasible; however, we implemented measures to reduce social desirability bias, including careful explanation of the value of both arms to participants and standardised assessor protocols.

To maximise the potential impact of the intervention, it is essential to explore the long term sustainability of its effects. This would involve tracking participants over an extended period to assess whether the observed benefits are maintained and identifying any factors that could influence the durability of these outcomes. Additionally, pinpointing the most effective components of the intervention is critical. By understanding which elements—such as specific therapeutic techniques, family involvement, or the use of culturally adapted materials—are driving the positive results, future iterations of the programme can be refined and optimised. This targeted approach will ensure that the intervention remains efficient and effective, providing the greatest possible benefit to those at risk of self-harm and suicide.

### Conclusion

The YCMAP intervention was shown to be beneficial in self-harm prevention among adolescents. Further research and replication in diverse settings are recommended to strengthen the evidence base for this public health intervention.

**What is already known on this topic**

Self-harm in adolescents is a major public health issue, with a high risk of recurrence and progression to suicide, especially in low and middle income countries
Previous studies have shown that cognitive behavioural therapy based interventions can reduce self-harm in adults, but there is limited evidence on their effectiveness in adolescents, particularly in culturally diverse settings like Pakistan
Culturally adapted interventions are needed to address the unique sociocultural factors influencing adolescent self-harm in low and middle income countries

**What this study adds**

A culturally adapted cognitive behavioural therapy based intervention, YCMAP (youth culturally adapted manual assisted problem solving), was found to significantly reduce the recurrence of self-harm and associated psychological burden among adolescents in Pakistan
The findings highlight the potential of culturally sensitive interventions to reduce self-harm in low and middle income countries, providing a basis for replication and implementation in similar contexts

## Data Availability

Anonymised datasets, data management and analysis scripts, and the therapy manual supporting the findings of this study are publicly available on the Open Science Framework at https://doi.org/10.17605/OSF.IO/6JQ8K. Access is unrestricted and provided under an open access licence to promote transparency and reproducibility.

## Web extra

Extra material supplied by authors

Web appendix 1: YCMAP manual

Web appendix 2: Supplementary tables and figure

## Notes

Contributors: NH, NC, IBC, and TK conceived the idea of the trial. SuS, ATN, MAA, SaSh, and UA supported identification, recruitment and retention of the trial participants. ST managed the trial as trial manager and monitored and supervised research teams. TK, CW, ZFZ, and FN led on the cultural adaptation of the intervention and trained and supervised trial therapists. RM and FJ worked closely with the patient and public involvement and engagement and community engagement officers throughout the trial. EC supervised the qualitative component of the trial. SE and RM led on ethics and governance.RE worked on the design and analysis plan of the trial, and RE, MP, SuS, and Steven Lane performed the statistical analysis and described the findings in results section. ST, TK, AW, and FM prepared initial draft of the manuscript. All authors contributed to the interpretation of the results and reviewed and edited the manuscript for intellectual content. All authors approved the final version of the manuscript and agreed to be accountable for all aspects of the work. NH is responsible for the content of this manuscript, as guarantor. The corresponding author attests that all listed authors meet authorship criteria and that no others meeting the criteria have been omitted.

Transparency: The lead author (the manuscript’s guarantor) affirms that the manuscript is an honest, accurate, and transparent account of the study being reported; that no important aspects of the study have been omitted; and that any discrepancies from the study as planned (and, if relevant, registered) have been explained.

Dissemination to participants and related patient and public communities: For dissemination to study participants, we will disseminate infographics summarising YCMAP through study social media channels and partner organisation websites. A press release will be prepared for national newspapers and FM radio health slots timed with World Suicide Prevention Day (10 September 2025).

Provenance and peer review: Not commissioned; externally peer reviewed.
