# Supplementary material for: Effectiveness of YCMAP (youth culturally adapted manual assisted problem solving) intervention in adolescents after self-harm in Pakistan: multicentre, randomised controlled trial
Source: BMJ. 2025 Sep 12;390:e083272. doi: 10.1136/bmj-2024-083272 (PMC12426884; doi:10.1136/bmj-2024-083272)
Supplement: Supplementary file 1 — Web appendix 1: YCMAP manual [file husn083272.ww1.pdf]

Culturally Adapted Manual Assisted Problem Solving Intervention for Young People who Self Harm (Y CMAP)

Pakistan Institute of Living and Learning (PILL)

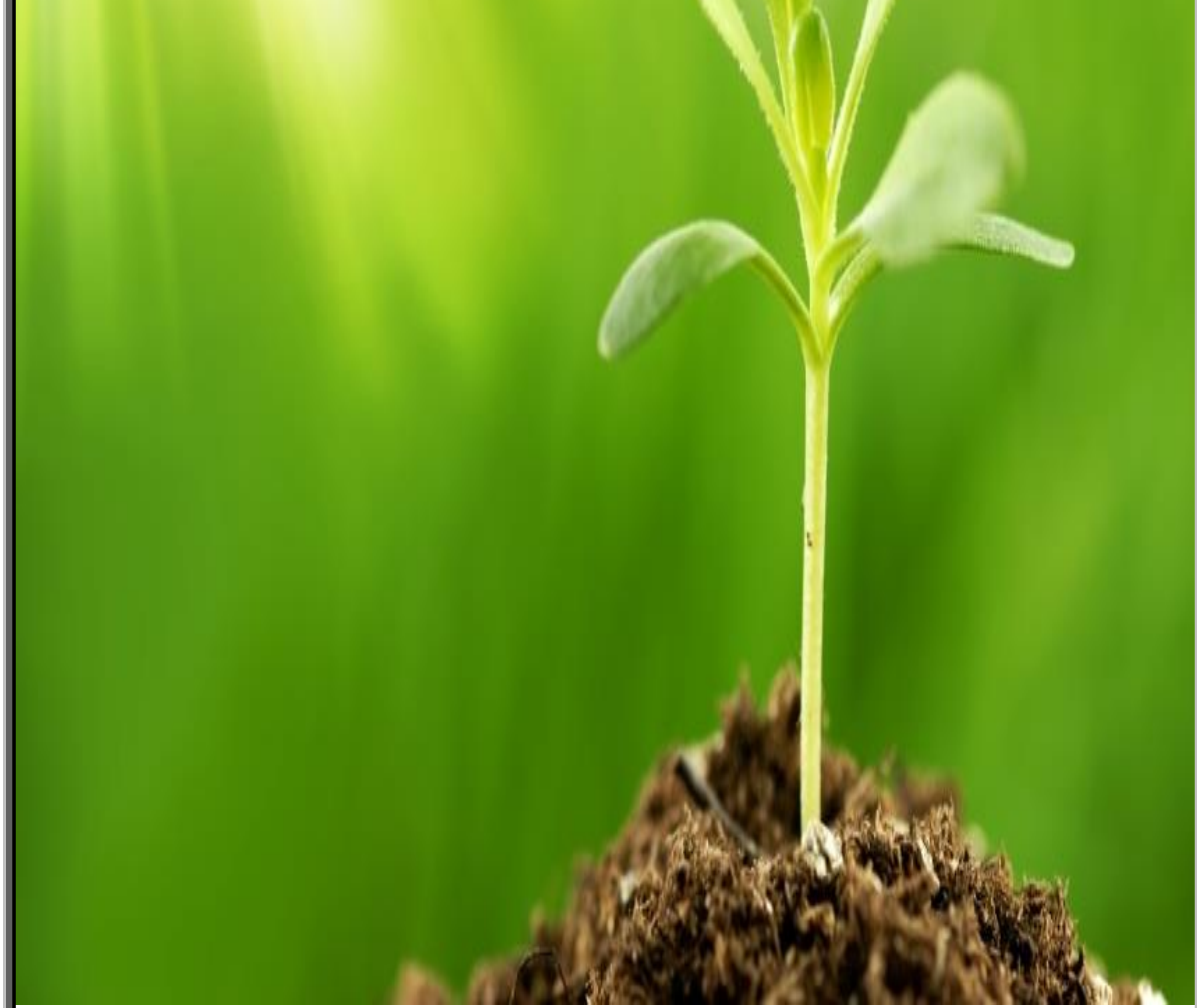

Adapted from;

Life After Self-Harm: A Guide to the Future (Ulrike Schmidt and Kate Davidson)
Cutting Down:A CBT workbook for treating young people who self harm (Lucy Taylor, Mima Simic & Ulrik Schmidt)
CMAP (Pakistan Institute of Living and Learning)

Introduction

You have recently attempted to harm yourself. Perhaps it is the first time in your life; perhaps it is something you have done several times before, when you felt unable to cope with problems, tensions or crises. You may have intended to die, or harmed yourself for a different reason, or perhaps you are unsure about why you did what you did.

At the moment you may be experiencing a mixture of feelings and thoughts. You may still feel extremely shaken up by what you did. Part of you may feel pleased or relieved to be alive. You may feel angry that someone stopped you from harming yourself, or you may feel ashamed about what you did. You don't want to be reminded of what happened, as the problem or crisis that caused you to harm yourself has now blown over. May be the problem that led you to harm yourself is still looming as large as ever and seems completely impossible to overcome. Perhaps harming yourself is the only way you know of dealing with certain intense feelings or desires and nothing else works as well.

In any case you may be asking yourself why you should bother to attend these training sessions and use learned skills.. May be you are double minded about it. You may even be tempted to throw it away and declare it useless before you have looked at it. Feeling that everything is useless, is a very common feeling in people who are suicidal. Our intention in writing this training manual is to getting you to stop and think. Don't just say: "I am past being helped, I have tried it all and I know it all."

Our aim is to try to help you to understand why you got to the point at which you harmed yourself and to help you look at whether there might be different ways of dealing with the difficulties that you are facing. We cannot tell you what to do, but you should know that we have tried and tested this training manual with many different people who have come to see us with similar difficulties as yours. Many of them have found it helpful. So perhaps you should buy yourself some time to practice these acquired skills. You owe at least that to yourself. What do you have to lose?

Session 1

Getting started
Psycho education: What is self harm?

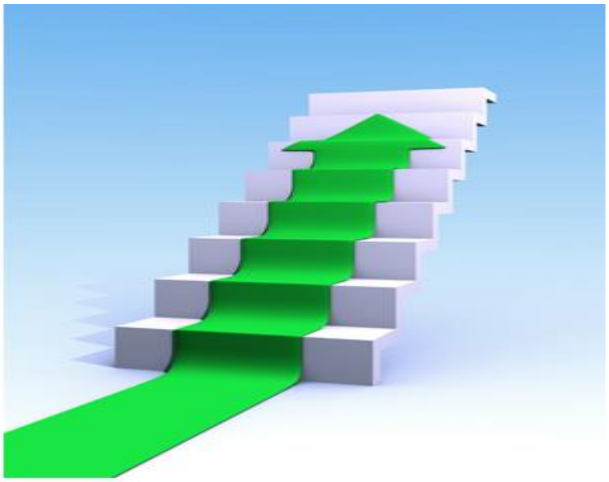

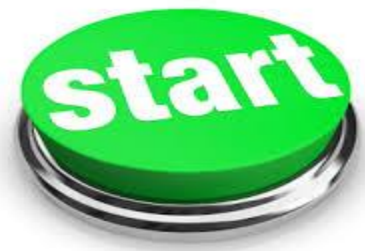

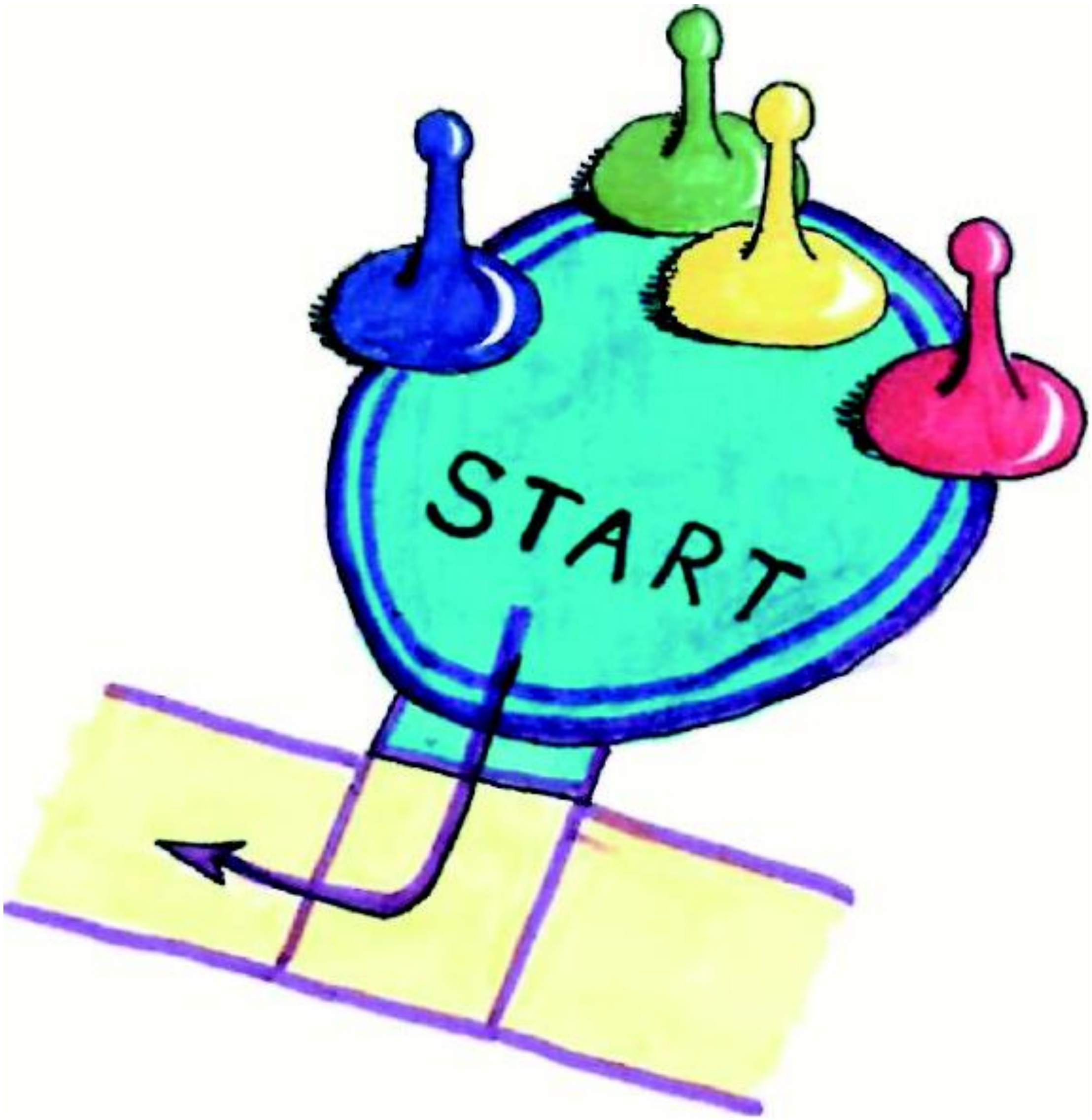
START

Aims:

This session focuses on psycho education about self harm, altering the young person to the fact that there are others who harm themselves and that it is a topic that has been fairly well researched. Psycho education is aimed to help young people feel less isolated, different, and to understand the mechanisms and function of behaviors.

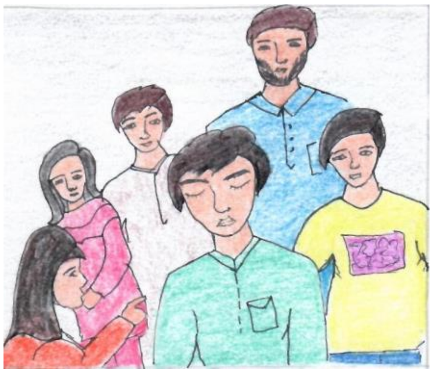

Agenda:

As this is the first session, the young person will probably not know what to expect. Explain, that you will be setting an agenda for each session which will always include;

1: Bridge from last session
2: Homework review
3: Any issues brought from the young person
4: Main session topic
5: Homework plan
6: Feedback

Why do people harm themselves?

There are many different reasons why people attempt to harm themselves. For some people harming themselves is like "pulling an emergency brake on a run-away train". They feel their life has got out of control and they don't quite know how to gain control again or make anyone notice.

'Real-life stories'

We talk throughout this program about 'virtual' stories of four people who have harmed themselves. You may feel you have things in common with some of them. We will introduce them properly later, but for now,here is a snapshot of their stories.

JAMILA
'I've done it forever . . . I can't imagine what my life would be like if I didn't self-harm'

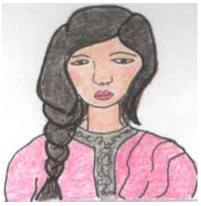

SARA
'The nothingness inside just takes over . . . I cut myself to feel something, anything'

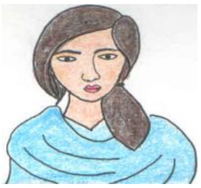

Ahmed
'I cut myself to get rid of the crap feelings inside . . . I like the sight of my blood, it makes me feel better'

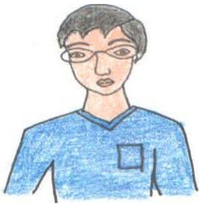

KIRAN
'The arguments and stress built up inside me until I couldn't handle it anymore . . . that's when I took the pills'

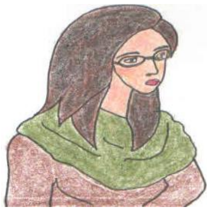

Give this page to participants

Trying to Understand Your Reasons for Harming Yourself

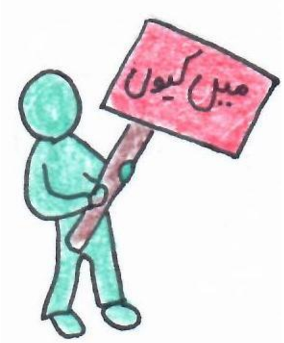

Many people find it very difficult to put into words why they harm themselves, because conflicting thoughts and feelings were present at that time, including those that most people don't like to own . You feel you'd rather forget about what you did. At the time you were too emotional or had taken some drugs so it is difficult to remember the state of mind that you were in. Perhaps you are the sort of person

who very quickly flips from one state of mind to another with little memory for what you felt before.

It is often very helpful, to try to remember exactly what happened and to talk to your therapist, health worker or another trusted person about it. The questions below are designed to help jog your memory. Answer the questions, if necessary with the help of your therapist. Do not worry if you do not have the answers to all the questions.

Note:

Therapist shall write down answers in the spaces given after each question and if necessary continue on a separate sheet.

How you feel before you self-harm
Note: you can also discuss these questions with someone you trust

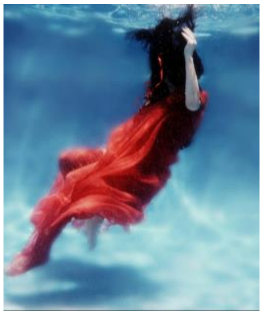

Think of your most recent experience of self-harm and answer any of these you feel comfortable with:

What happened?

What let you to do it?

What did you feel before?

Was there anything else in the background (something current or an echo from the past)?

Did you spend long time thinking about harming or was it spur of the moment, or both?

Is that your usual way (if not, what was the difference)? What else was important at the time (events, thoughts, memories, exhaustion, voices, etc.)?

The Function of self harm

This section continues with psycho education about self harm and also starts to elicit the particular functions of self harm for the young person.

Start by discussing some of the documented reasons young people have given for using self harm and ask the young person which they relate to and what other reasons of their own they might add to the list.

Remember, the list is not exhaustive.

Reasons why young people might self harm

- *Self harm can be about reducing tension.*
- *Self harm can be triggered by feeling hopeless; feeling that the harder you try, the more difficult things become.*
- *Self harm can provide a relief from thinking or feeling.*
- *Self harm can be about, wanting to die.*
- *Self harm can be a response to sudden mood changes.*
- *Self harm can be a way to gain control over life.*
- *Self harm can be an expression of anger.*
- *Self harm can be a way of managing the difficult emotions someone has after being hurt or abused.*
- *Self harm can be a way to punish yourself for other people.*
- *Self harm can be an expression of guilt.*
- *Self harm can help people to cope with emotional and psychological pain.*
- *Self harm can also have a calming effect. Some young people feel soothed by the sight of their blood or by caring of their own wounds.*

Timeline of self harm: Jamila

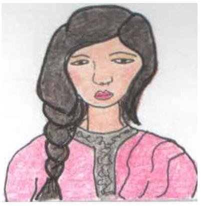

Following this discussion, you should have a few clues as to some of the reasons why the young person engages in self harm. The next step is to get an understanding of when the self harm started, the triggers and the frequency of occurrence.

Exercise: Timeline of self harm

Use “worksheet: Timeline of self harm” and explain that the aim of this exercise is to think about how self harm has fitted into the young person’s life and for how long. You will focus on the history, severity and frequency of self harm(Note: incase your participant had only one episode of SH, please collect information for that episode only. Please complete this activity relevant information from participant’s history)Discuss Jamila’s example prior to carrying out the exercise.

- First the young person needs to identify several “anchor points” to help remember where and why self harm occurred: For example, key events like birthdays, special occasions, or school changes etc. These are then, written around the time line using arrows.
- Next, other incidents can be added around the timeline, generated by a series of questions. “What is your earliest memory?” “Describe big event that pops into your head”, “What is your happiest/saddest/most exciting memory?”.
- Then, may be in a different color, incidents of self harm can be added(incase of single attempt please mention that attempt only), again generated by a series of questions. “Do you remember when it started?”, “when was the worst time/the most worrying time for others/the most worrying time for you?” etc.
- Then the discussion should be opened out and a chronology of incidents must be identified. “Has the self harm changed over time?”, “have you developed routines?”, “Do you do different things now?”(this section would only be relevant if participant had more than one attempt).
- As the timeline is being completed, start to explore with young person which particular events were happening around the various incidents/times when

they self harm. This data will be used for formulation and will reveal clues to predisposing, precipitating and maintaining factors.

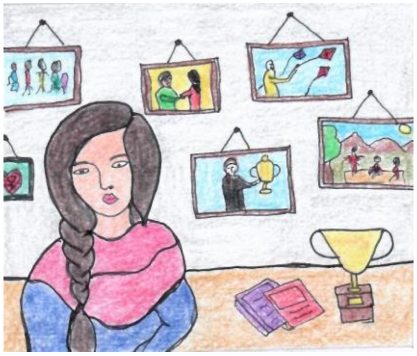

SOME FURTHER THOUGHTS

This section can be explain to the participant as

"It is very easy after you have attempted suicide, to feel very pressurized by others around you (including your therapist!) to give up being suicidal. It is likely that your attempt means a lot of different things to you, positive and negative and that at different times you feel different things about it. Now you have had a chance to reflect on what has happened that led to self harm, what your thoughts and feelings at that time, let's look at these issues more objectively.

What are the advantages and disadvantages of self-harm .

You or a member of your family may be shocked and appalled to hear that we are trying to get you think about the positive side of suicide.

You may think that we are trying to push you over the edge, or that we are trying to play clever, but very dangerous games with you. This is definitely not the case, if nobody talks with you openly about the advantages of harming yourself or suicide, you may go on in private having these thoughts. It is likely that these will linger and grow stronger and left on your own, you may find it very difficult to recognize the catch or the many catches that these thoughts always have.

Worksheet: Timeline of self harm

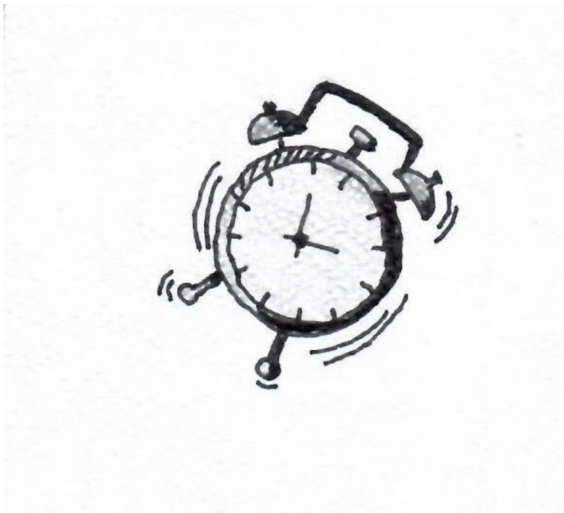

Age

0 18

Session 2

What to do in a crisis

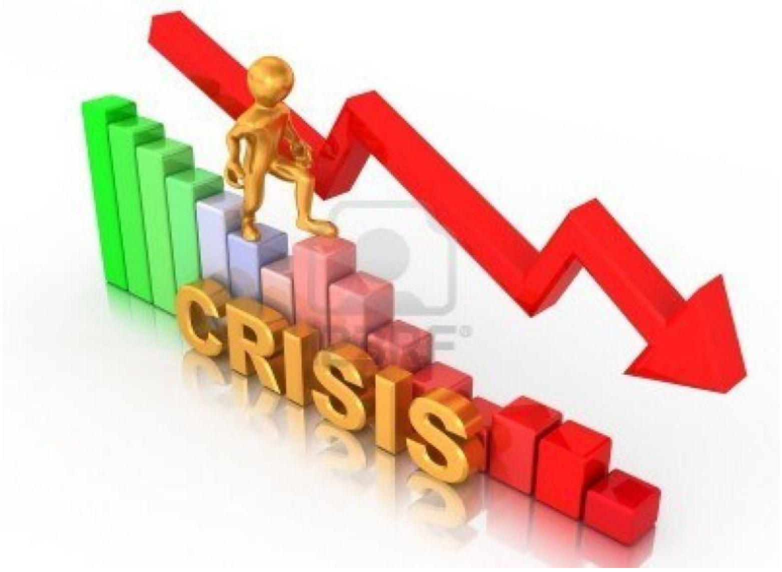

What to do in a crisis:
Even if at present young person is full of good intentions to work on reconnecting with life, it is very likely that he/she will come across considerable crises and difficulties and that may end up feeling suicidal again. The important thing is to be prepared for this and to think now what they can do if such a crisis occurs again. Over the course of their therapy and as they work through this manual, they should become better at dealing with any crises, but there are a number of things they can do right now, to stop from sliding down.

Aims:
In this session therapist will help them develop a written plan for dealing with any crises. This plan should contain important phone numbers of whom to contact when things are getting difficult again, and a number of ways of coping when they notice themselves getting more distressed and suicidal.

Agenda:
1: Bridge from last session
2: Any issues raised by the young person
3: Main session topic
4: Homework plan
5: Feedback

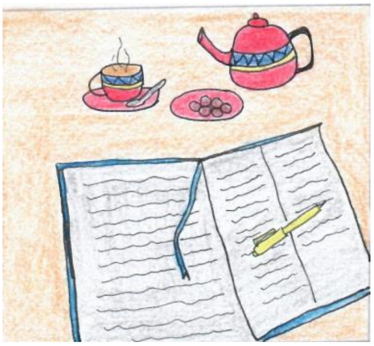

Main session topics:

Getting support
Start with following questions;
Is there anybody you can call or visit if you-are beginning to feel like harming yourself again? Or even stay with them for a little while? Is there anybody amongst people around you who you could use for support in a crisis, who is likely to listen, and give you time and space? Can you talk to this person now to discuss that you may need their support if in crisis? Write down their name and contact number on the crisis list below.

Keeping yourself safe
Ask following questions;

Do you store tablets at home, for example painkillers, sleeping tablets or antidepressants, etc? Do you keep razor blades, or the sharp knife that you used to cut yourself, or a noose? Just in case you may feel suicidal again, as a kind of insurance policy? You may say: "Yes, and I am very well going to hang on to these things. They make me feel safe." Try to think about it differently. By getting rid of your tablets and other "weapons" can be used to harm yourself, you will decrease the risk of any impulsive action.
Use of illegal drugs will greatly increase the risk of sliding into feeling suicidal again.

Trying not to slide down into a pit of loneliness and despair
Below a menu of different coping skills is given that may be of help in getting the upper hand on young person's distress. Some of the coping skills described may seem useless or even totally mad to them. Well, not everything works for everyone. The different skills described below are just ideas for young persons to choose from. There is no right or wrong way of dealing with distress. They may wish to add further strategies, not on the list.

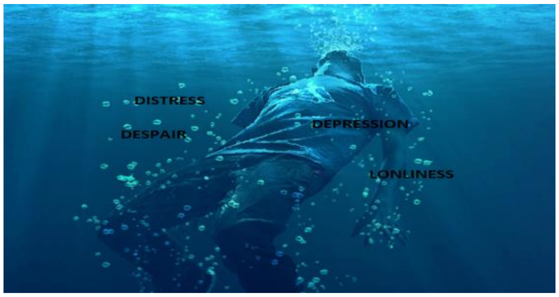

Do you really want to make a life or death decision on the spur of the moment, on impulse at a moment of great distress? Death is forever, whereas distress may only last a while.

Don't expect miracles! Remember practice makes perfect!
Say;
"Go through the list given below, carefully. Choose those coping skills that seems promising for you, think through whether the skills you have chosen will help you deal with negative feelings, thoughts and suicidal urges, wherever you are and whatever the time of day is. For example, if your main strategy for coping with distress is to ring a friend or to go out, this may not be terribly helpful if you frequently feel extremely lonely and suicidal at 3:00 a.m. in the morning (unless you have friends

who do not mind being called up at 3:00 a.m.). Discuss your chosen strategies with your therapist and think them through carefully.

You should also think about what you will do if a chosen strategy doesn't work, (It may be that some things work well helping to prevent distress or when you are not, yet, feeling very low, but that you need to back these up with other strategies, for when you feel very distressed and suicidal). **Write down an exact plan of action and then stick to it**. Add this to your crisis plan, but try to practice your skills for coping with distress regularly, even when you are not feeling too down. Monitor carefully how well the techniques, you used, worked for you.

Exercise: Activity scheduling

Explain to the young person the rationale behind this exercise: the goal of activity scheduling is to maximize engagement in mood-elevating activities. 1: explain the young person that depression or low mood and inactivity can become a vicious circle. It slows you down, mentally and physically, and makes everything an effort. You get tired more easily, you do less, and then you blame yourself for not doing anything, and that you will never get over it. That makes you even more distressed. It becomes even more difficult to do anything. And so it goes on.

Becoming more active is one way to break this vicious circle

Being active helps you;

- To feel better and less tired.
- To motivate yourself to do more.
- To improve your ability to think.
- To structure your time and help you feel that you are taking control of your life again.
- To increase pleasurable and fun activities that are missing.
- To make sure that you have a balance between things that gives you a sense of achievement as well as fun.

Explain that you are going to put your heads together to identify any activities (big or small) that may be pleasurable or might have been pleasurable in the past.

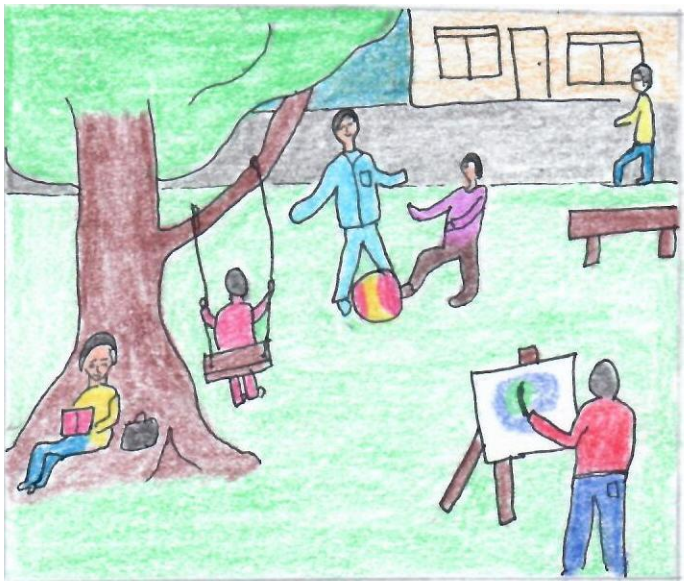

“Positive pleasant events”

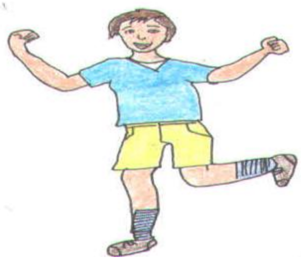	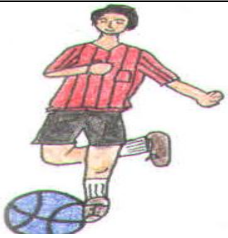	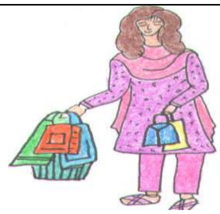
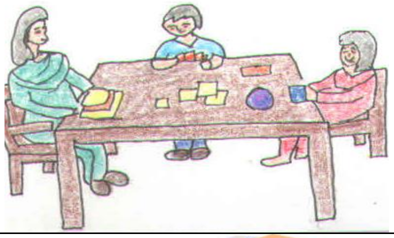	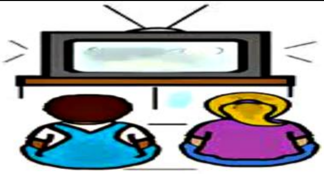	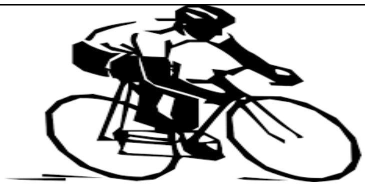
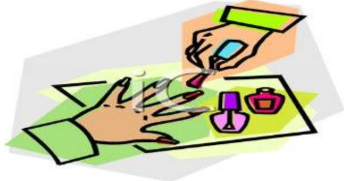	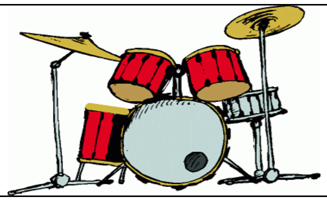	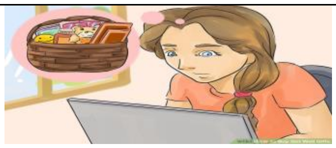
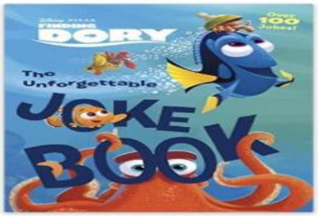	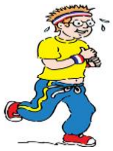	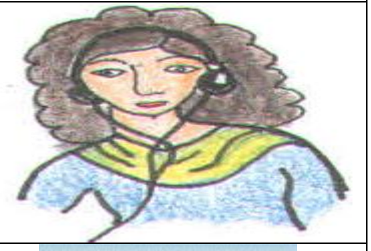
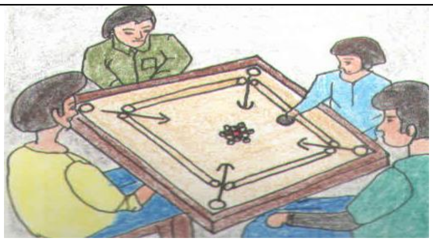	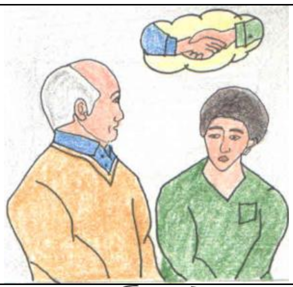	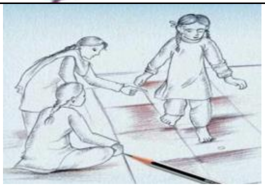
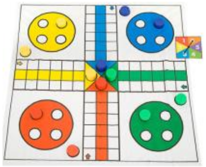	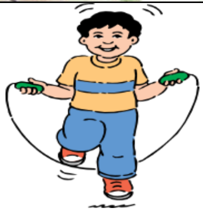	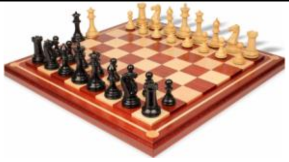

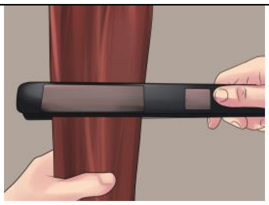	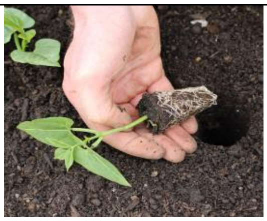	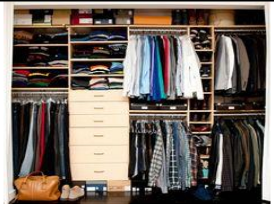

1: Going to the Park	24: Changing my room around
2: Play cricket	25: Doing my hair/dyeing it/straightening it
3: Video game	26: Reading jokes from a joke book
4: Play Luodo	27: Meeting up with an old friend
5: Play Carrom	28: Playing a musical instrument
6: Going to watch a film	29: Writing poems or stories
7: Recalling funny memories	30: Going to a beauty salon
8: Laughing	31: Photography
9: Listening to others (Grandparents)	32: Watching a favorite program
10: Reading a magazine	33: Going for a bike ride
11: Going for a run	34: Buying gifts
12: Going for shopping	35: Dancing
13: Arranging to meet a friend	36: Playing cards
14: Drawing	37: Spending time with grand parents
15: Looking after a plan or pet	38: Planning activities for the future
16: Painting my nails	39: Relaxing
17: Writing in my diary	40: Listening to music
18: Doing a puzzle	
19: Going on a picnic	
20: Cooking	

21: Getting a massage	
22: Going bowling	
23: Sitting in a café and watching people	

Crisis Plan

Crisis contact numbers:

Therapist's name and contact number:

GP:

Local hospital accident and emergency department:

Contact of psychiatrist:

Other phone numbers:

If I should begin to feel like hurting myself again I will call one of the following people:

(1) ____

(2) ____

(3) ____

If I can't get through to anyone I will use the following strategies:

(1)

(2)

(3)

(4)

(5)

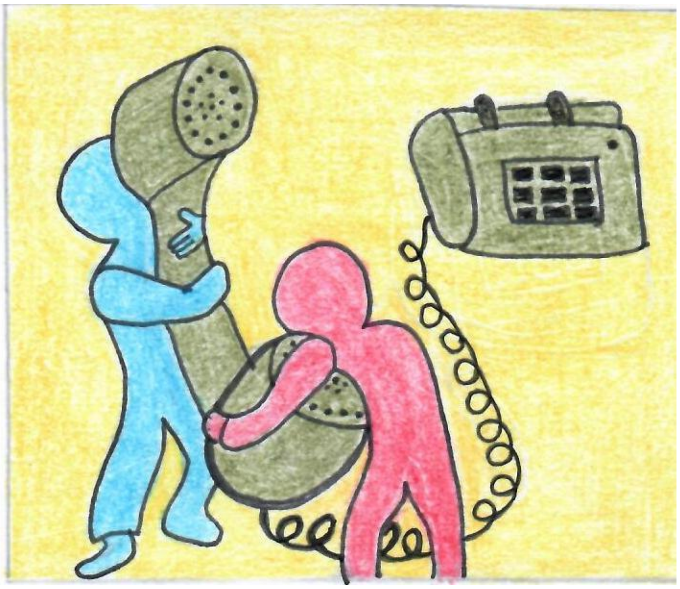

Your crisis plan may need reviewing and updating on a regular basis. You may also wish to have several copies of it, one to carry around with you, other copies at home left in places where you can easily find it, e.g. near the phone, above your bed or pinned at your fridge.

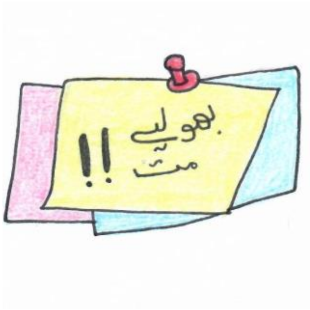

- Keep yourself safe by getting rid of tablets and other "weapons" likely to be used to harm yourself.

- Produce a written plan for dealing with crises and keep it with you at all times. Update it regularly.

- Practice your chosen skills for coping with distress on a regular basis. Practice will makes perfect.

Session 3

Cognitive Behavior Therapy Emotions and Feelings

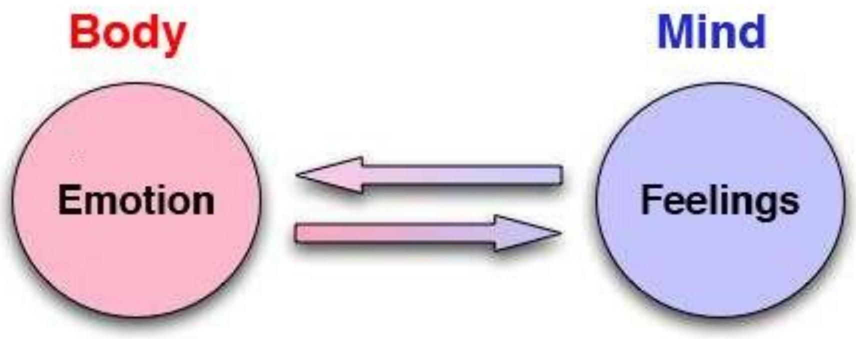

This session is comprised of following components;
We will start by identifying the young person's problems and specific goals they want to achieve in the therapy.
After this, we will introduce the CBT model and describe how it can help them to achieve their identified goals.
Finally, in this session, the first key aspect of CBT – "feelings" – will be introduced.

Aims:

- Identifying problems and goals.
- What is CBT
- What are emotions?
- Keeping a feelings diary/record.

- Agenda:

1: Bridge from the last session.
2: Homework review.
3: Any issues raised by the young person.
4: Main session topic.
5: Homework plan.
6: Feedback.

Problems & Goals:
The aim of this section is to get full understanding of the young person's current difficulties, definition of the problem, how severe they are and to identify the specific goals they want to achieve in therapy.

- What areas are problematic in the young person's life?
- Define the problems clearly and specifically.
- Break down vague goals into behavioral and measurable concepts. They must be realistic and clearly defined, so that it is clear when they are achieved. For example, if the young person says, 'I want to feel better', ask them, 'how would and/or others know you were feeling better about yourself?' and 'what would you be doing differently if you felt better?'

Kiran's Problems	
Problems	Rating
1. Difficulty in getting on with my mum	9
2. Feeling bad about myself	8
3. Cutting my arms	8
4. Not being able to say what I think	5

Note that the young people sometimes believe that there is a great discrepancy between how they feel compared to how they would like to feel, or where they would like to be. This can often lead to a sense of hopelessness (which in turn may contribute to an episode of self harm). When discussing goal setting, recognize this difference and try to introduce a sense of hope that realistic achievements and changes can occur by working together and by learning the CBT strategies.

Kiran's Goals

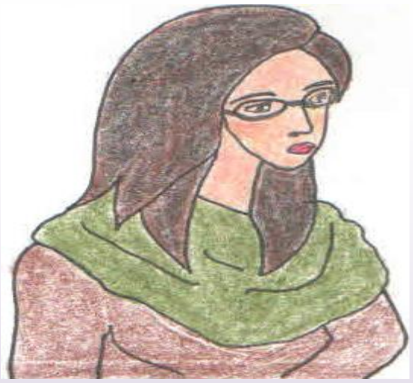

- Fewer arguments with mum; more fun time with her. Talk to each other more (calmly).
- Increased confidence (saying what I want, more to my friends, making decisions myself, not worrying about asking teachers for help), going back to trying to look good, e.g., putting on make-up, nail varnish, etc., and taking care of my appearance.
- Stop cutting – find different ways to manage feelings.
- Learn to stick up for myself and say what I think. I need to believe that I have a valid opinion and that it matters as much as other people's.

Worksheet

Problems and goal setting

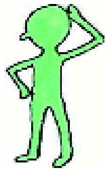

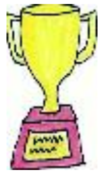

Problems<br>(Scores 0 -10)	Goals

What is CBT and how does it relate to my self harm?
Aim:
This section aims to educate the young person about the main principles of CBT and how this way of working might help them reach their goals. It also links the functional analysis of their self harm, as discussed in session 1, to CBT by highlighting the relationship between thoughts, emotions, behaviors and environmental factors. It aims to educate the young person the principles of CBT and how it is useful in recognizing triggers and maintaining factors in their self harm behaviors. This will lead on to the strategies to monitor and challenge thoughts directed to self harm, and learning alternative behaviors to manage intense emotions that have historically led to self harm.

Exercise: Two scenarios

Use **“worksheet: What is CBT”** and discuss the following examples with the young person.

Imagine, that you are at home alone (others in the house have popped out for a while) and you are sitting downstairs when you hear a loud bang upstairs (see figure 1). Take the young person through the two possible scenarios, and encourage them to think specifically about the difference between them.
Explain to the young person that in both Scenario 1 and Scenario 2 the situation is exactly the same, but the emotions/feelings experienced vary depending on how the noise is interpreted. Ask them to write down, in their own words, the connection between thoughts, and emotions and to generate examples of their own when they have noticed feeling strong emotions about something (a happy time or a time when they felt very angry). Then ask them to identify the associated thoughts and behaviors. Use **“Worksheet: The help triangle”** to record the various examples.

Explain that in CBT, they will be learning ways to manage difficult emotions by becoming expert at identifying their thoughts. When they can do this, they will learn the ways to challenge distorted thinking (thinking bias), find alternative, more adaptive ways of thinking and in turn alter what they do in response to the thoughts.

Worksheet
What is CBT?

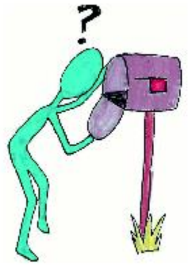

The Cognitive-Behavioural model – what's it all about?

Cognitive Behavior Therapy is an effective way of helping people to deal with their problems. It explores the link between what we think, how we feel, and what we do, our behavior.

For example:
Feeling Sad:
Thinking that you are not very good at talking with people may make you feel very worried, when you are out with your friends. You may go quiet (behaviour) and not talk very much.

Thinking that no one likes you may make you feel sad. You may stay at home alone.
Feeling Angry:
Thinking that you never get things right may make you feel angry. You may cut yourself to get rid of this feeling. You may then feel sad because, yet again, you have done something wrong.

Let's look at a more detailed example and think about how the thoughts that go through your mind affect how you feel and what you do (your behavior).

Worksheet
"Help Triangle"

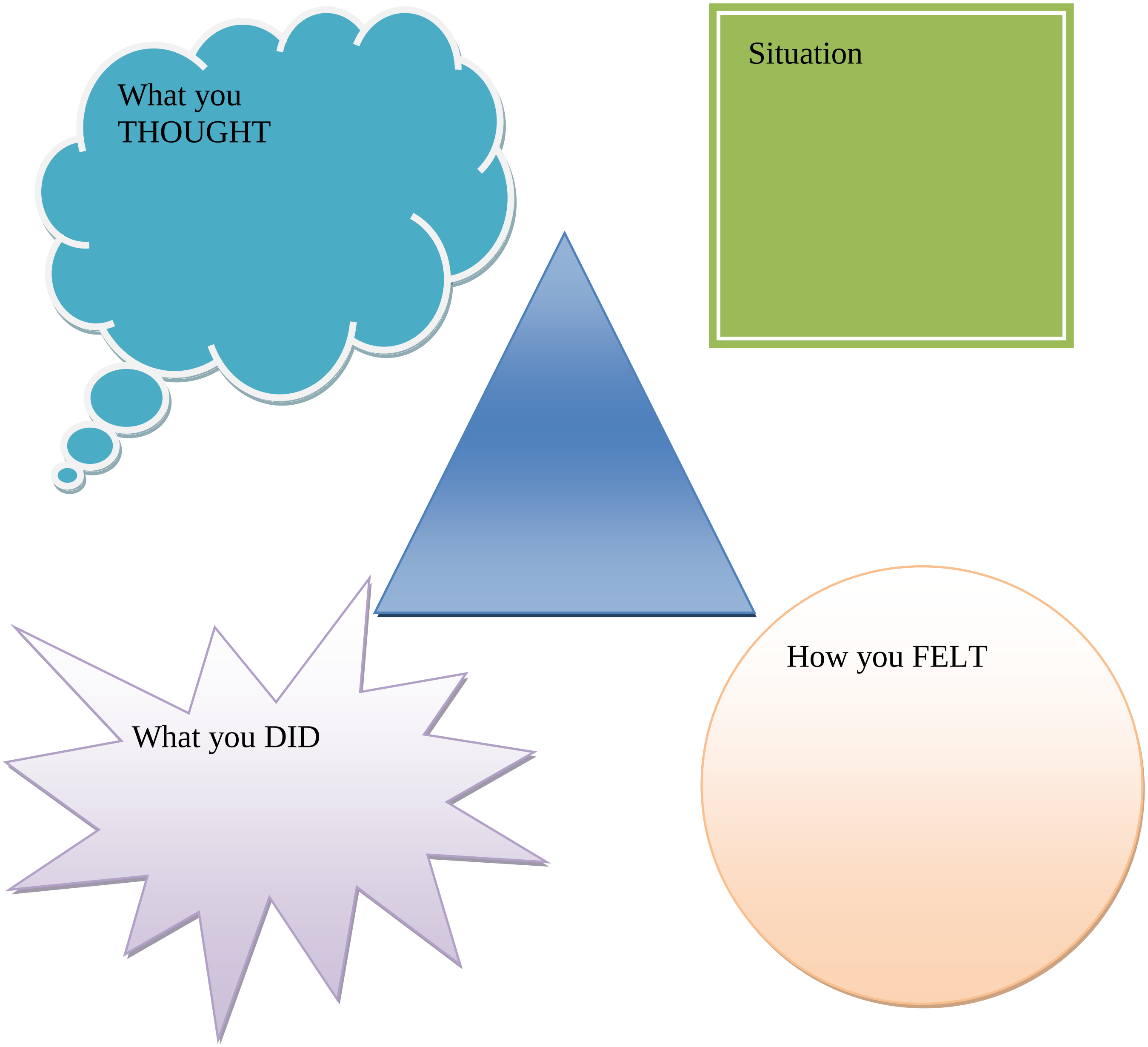

Figure 1

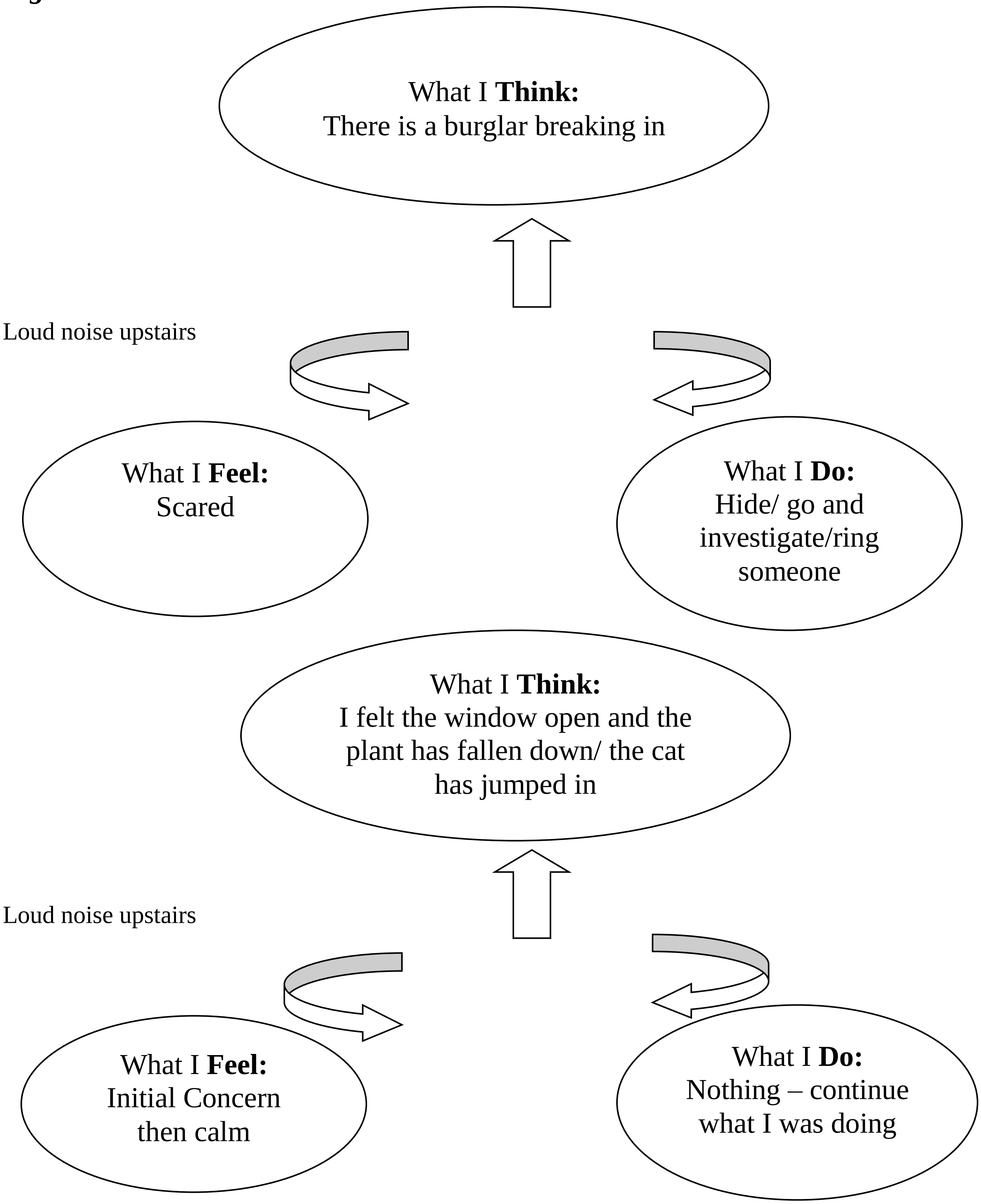
What I **Think:**
There is a burglar breaking in
Loud noise upstairs
What I **Feel:**
Scared
What I **Do:**
Hide/ go and investigate/ring someone
What I **Think:**
I felt the window open and the plant has fallen down/ the cat has jumped in
Loud noise upstairs
What I **Feel:**
Initial Concern then calm
What I **Do:**
Nothing – continue what I was doing

Psycho education

What are emotions?

The feeling of an emotion is a bit like someone knocking on your door to deliver a message. If the message is urgent, the knock is loud; if it is very urgent, the knock is very loud. If it is very urgent and you do not answer the door, the knock grows louder and louder until you either open the door or it is broken down. Whichever, the emotion will continue to bang away until it is acknowledged. As soon as you open the door by listening to the message, the emotion can be dealt with and it will eventually disappear.

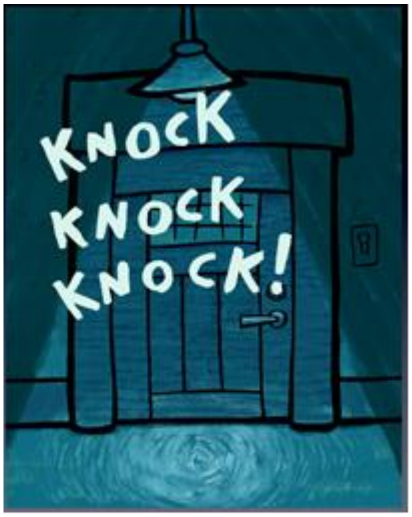

Explain to the young person that they may not experience it like this at the time: they may just want to get rid of the emotion as quickly as possible. At times, self harm may seem like the only option, when emotions are felt very strongly.
People often struggle to know which emotion they are feeling. Discuss the analogy of emotions mixing together like squash and water, which makes it difficult to tease them apart and name them separately. If this happens, it can feel very overwhelming for the young person.

Exercise: Different emotions

This exercise aims to teach the young person about different emotions, how to separate them from each other, and how to make them easier to understand and deal with it.

Use the **“worksheet: Unmixing the feeling cocktail”**. Tell the young person that we are going to look at their feelings in more detail. First they should look at the list of feelings in table 2.

Table2: Emotions

Scared	Afraid	Confident
Brave	Happy	Content
Lonely	Tearful	Calm
Hurt	Anxious	Disgusted
Disgusting	Out of control	Relaxed
Jealous	Proud	Ashamed
Embarrassed	Frustrated	Miserable
Disappointed	Helpless	Exploited
Furious	Uncomfortable	Love
Sad	Guilty	Excited
Worried	Bored	Annoyed
Used	Grumpy	Mad
Upset		

Instructions: therapist donot necessarly share the complete list. This list can be used as a prompt when young person is not able to name emotions or vocal.
Ask the young person to pick three of the emotions from the list. Depending on the client, you could use one of the several different methods, depending on their level of emotional literacy and what they prefer;

- Close your eyes and randomly select three different emotions.
- Pick out the three emotions you most identify with.
- Pick out the three emotions you least identify with.
- Pick out the three emotions that other people tell you they think you exhibit.

Once they have picked up their three emotions, ask them to read each one aloud and describe what it means as best as they can. Make it clear that this is not a test some emotions are hard to describe. The idea is to try to get an impression of how the young person understands emotions and their emotional vocabulary.

Following this, talk about feeling scale. Use **"worksheet: Feeling scale"**, and encourage the young person to rate how intensely they feel certain emotions (10 = most intense; 0 = no intensity).

Objective of Activity: Enhance awareness about emotions leading to improved to self control or self evaluation.

When young person is not to express himself/herself fully he or she feels even more frustrated this exercise will may help them to find better words to express their emotions

Next, show the young person, Sara's example. Then ask them the following questions about the first of the three emotions they chose earlier.

- Can you give an example of when you have recently felt like that or when you have observed a friend feel like that?
- Can you describe exactly what was happening?
- Can you remember what you were thinking at that time?

Sara's Example:

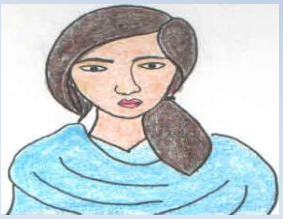

Sara often feels numb inside, which is when she cuts. She recognizes the following emotion that she feels she struggles to tolerate.
Scared: When your heart races so fast, you think you are going to die and something horrifying is going to happen. The last time I felt like this was when I had to go to my therapy group. I thought I would be judged and everyone would hate me. Then, I felt even more scared and anxious. I think that if another person of my age felt scared, would also feel the physical sensations and might avoid doing the things.

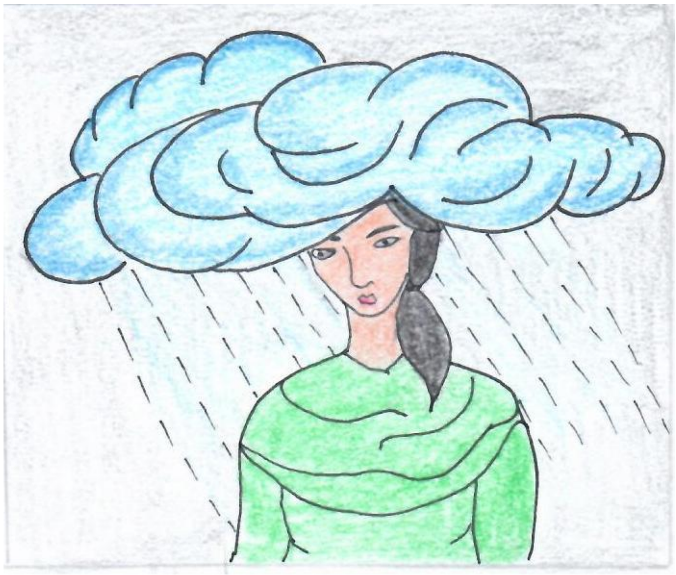

Worksheet: Feeling Scale

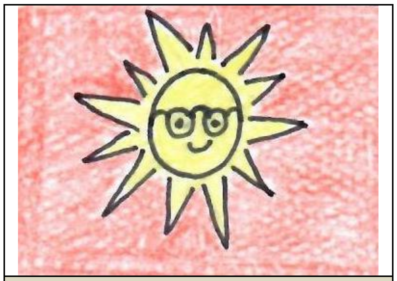
10<br>9<br>8<br>7<br>6<br>5<br>4<br>3<br>2<br>1<br>0
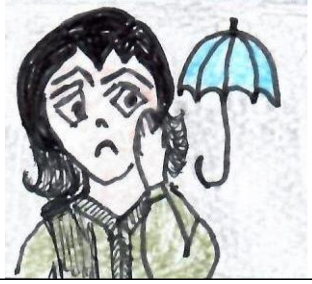

Next, ask the young person to describe their personal experience of other two emotions in the same way.

Homework:

Give the young person **“worksheet: Emotions diary”** as their homework task for this session. Following on from this homework, you can draw on their examples when completing **“worksheet: feelings are our friends”** and **“worksheet: What feelings do I squash, bottle or swallow?”**

Worksheet: Emotions Diary

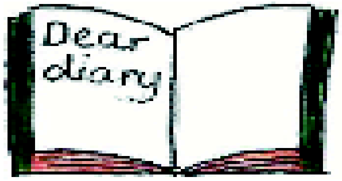

Keeping a diary of your feelings can be helpful in a number of ways. By looking back at the diary, you might discover that there is a link between what you were doing and how you felt. You might find that your feelings are stronger at certain times of day, or that they are not as frequent as you thought they were.

	Monday	Tuesday	Wednesday	Thursday	Friday	Saturday	Sunday
8 – 9am							
9 – 10am							
10 – 11am							
11-12pm							
12 – 1pm							
1 – 2pm							
2 – 3pm							
3 – 4pm							
4 – 5pm							
5 – 6pm							
6 – 7pm							
7 – 8pm							
8 – 9pm							
9 – 10pm							
10 – 11pm							
11- 12pm							

Worksheet: Feelings are our friends

Okay, so maybe that sounds a bit silly, but it’s true! Think about it for a minute . . . Most people would say that happiness is a positive feeling, and that anger is a negative feeling, almost one that you shouldn’t have at all. But we all have lots of different feelings at different times, even if some of them are pretty unpleasant! It’s not bad to feel angry; it’s what you do with the feeling that counts.

Let us consider some of the positive aspects of some of the more ‘difficult’ feelings

Note: It is not necessary that the young person can reflect on all the feelings. It is fine if they come up with just one related to their personal experience.If they can’t please give 2-3 examples

ANGER: Can give you strength to stand up for something<br>What does my anger do for me?	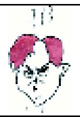
ENVY: Can help you strive for something<br>When has being envious helped me?	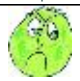
GUILT: Can help you change how you act<br>How has feeling guilty helped me?	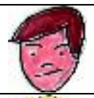
FEAR: Can help you to protect yourself<br>How has my fear helped me?	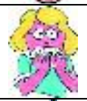
SHAME: Can help you to be more considerate of those you love in the future<br>How has it helped me to feel shame?	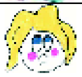
SADNESS: Can help you to move on<br>How has my sadness helped me?	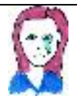

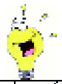

DISAPPOINTMENT: Can help you to be more realistic in your expectationsof others and yourself<br>When has my disappointment helped me?	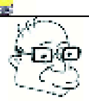

What have you found out about yourself from doing this exercise?

……………………………………………………………………………………
……………………………………………………………………………………
……………………………………………………………………………………

Worksheet: What feelings do I squash, bottle up or swallow?

Self-harm is often considered to be a way of managing overwhelming feelings such as anger, frustration, despair or sadness.

Sometimes it can feel like these intense feelings are so great that they will overflow like a volcano and this might be too much to handle. So, when we experience these feelings, we often try to find a way to manage them. Sometimes we might swallow our feelings or bottle them up in order to feel in control of them and some people may harm themselves to get relief.

For some young people, it can be difficult even to know how to describe such intense feelings. By unmixing the feelings cocktail earlier on, you may feel more able to identify and understand your feelings. The next task is to help you to identify those particular feelings that you try to squash or bottle up .

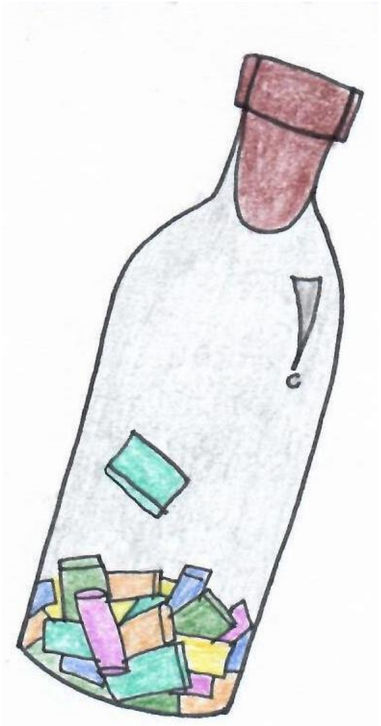

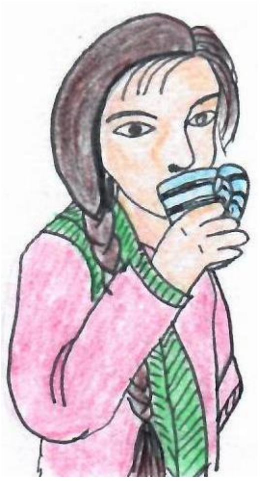

Session 4
Motivation to Change

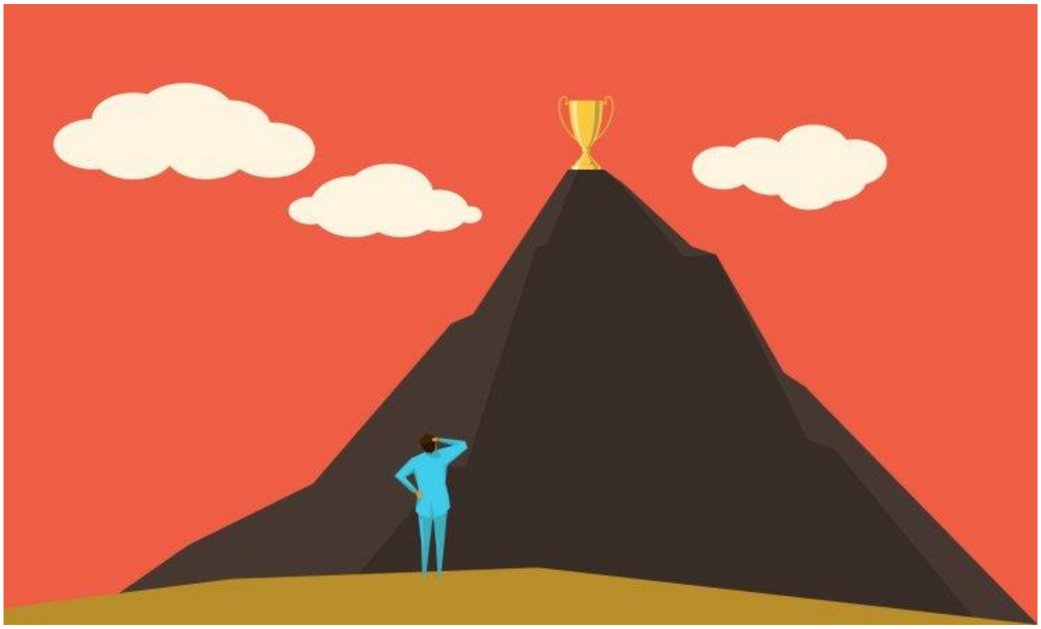

Aim:
The overall aim of this session is to continue to work from session 3 on emotions and get some sense of the young person's life and relationships outside of the therapy.

Agenda:
1: Bridge from the last session
2: Homework review
3: Any issues raised by the young person
4: Main session topic
5: Homework plan
6: Feedback

Main session topic:
Start session by reviewing the emotions diary homework and think about the next step in understanding emotions. Use the **"worksheet: Feelings are our friends"** and **"Worksheet: What feelings do I squash, bottle up or swallow?",** and work through these with the young person. If the young person could not complete the worksheet you can look at the Ahmed's example below. Once you have compiled this work, move on to the next step, which focuses on the young person's relationships and personal strengths.

Emotions are our friends: Ahmed

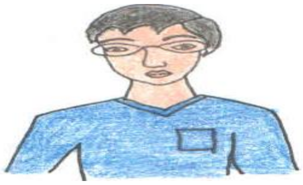

Okay, so maybe this sounds a bit silly, but it is true! Think about it for a minute. Most people would say that happiness is a positive feeling, and that anger is a negative feeling, may be, even one, that you should not have at all. But we all have lots of different feelings at different times, and some of them are pretty unpleasant! It's not bad to feel angry; It is what you do with feelings that counts.
Let's consider some of the positive aspects of a few of the more difficult feelings.

Anger: Can give you the strength to stand up for something
What does my anger do for me? Stops me being walked over.

Envy: Can help you strive for something
When has being envious helped me? When my brother started to beat me at running. That made me work harder to win.

Guilt: Can help you change how you act
How has feeling guilty helped me? When I take out all my anger at my mum, I feel guilty. It is not her fault (it's my dad). I can then apologize and try to make her feel better.

Fear: Can help us to protect ourselves
How has my fear helped me? Fear of dying has helped me to think about how to make my life better, so I actually want to live.

Sadness: Can help me to move on
How has my sadness helped me? I still feel sad, but I decided I could not deal with it on my own and it was too much. That is when I asked for help.

Squashed feelings: Note: ask for the feedback on young person’s reflection on types of feelings they try to squash or bottle up.(This was the home work given to young person in previous session)

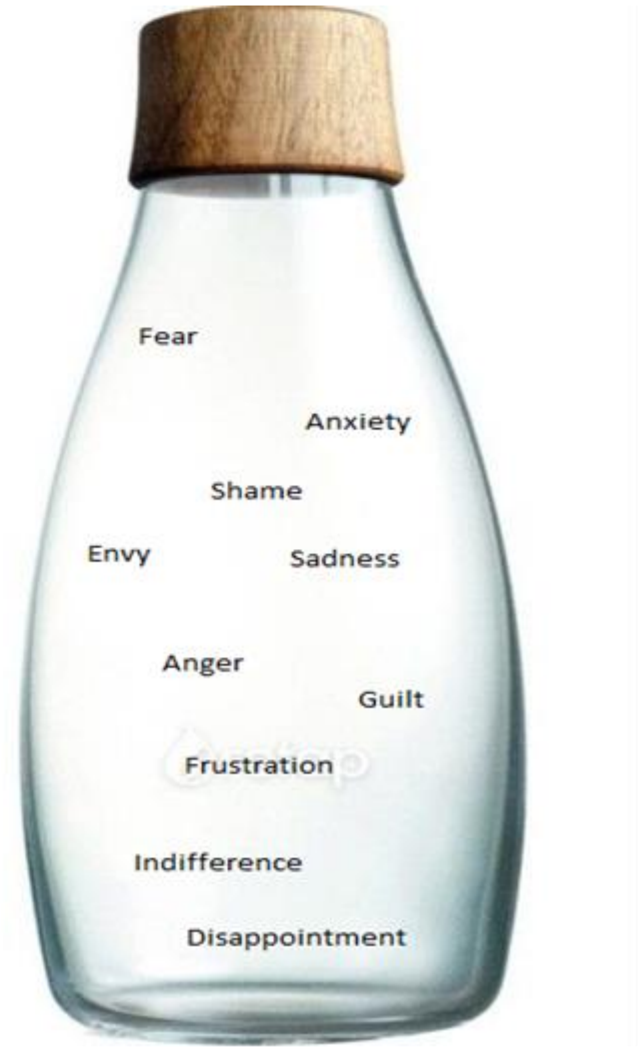

When you go through the exercises with the young person, ask them which feelings they tend to bottle up, or squash or swallow. You can use the appropriate sheet, or all of them. It is useful to ask why they think they do and what advantages or disadvantages they feel in using this strategy with their feelings.

Are you ready to make some changes?

The aim of this section is to assess motivation to change, in preparation for the next phase of therapy and to find alternative ways to manage problems other than self harm.

It is not unusual for people who self harm to feel ashamed or embarrassed about their way of coping. However, it can also be difficult for the young person to think of what their life would be like without self harm, and they can feel ambivalent or even unmotivated to give it up. Even so, the fact that they are starting to engage with this program implies that at least a small part of them (or someone who cares about them) is considering change.

Exercise

Use **"worksheet: Are you ready to make changes"** to assess the young person's motivation using the simple motivational rules. Then complete the questions with the young person. If motivation is assessed to be low, encourage the young person to weigh up the "cost" and "benefits" of their self harm and related problematic behaviors they may have started to discuss.

Look at what Ahmed said when asked how self harm is in his life. Then ask the young person the same question and discuss.

Are you ready to make changes?: Ahmed

Ahmed would like to stop hurting himself, but he is finding it very difficult to stop, particularly when he is feeling anxious or low. He describes how the sight of his blood helps relieve tension and induce a sense of calmness. It also makes him feel real. However, Ahmed recognizes that his self harm provides only temporary relief and is not the solution to his problems. Furthermore, he feels guilty that his self-harming behavior is causing more arguments between his parents and leading them more to worry about him. Ahmed has stated that he would like to overcome his urges to self harm by finding more healthy ways to deal with his difficult feelings.

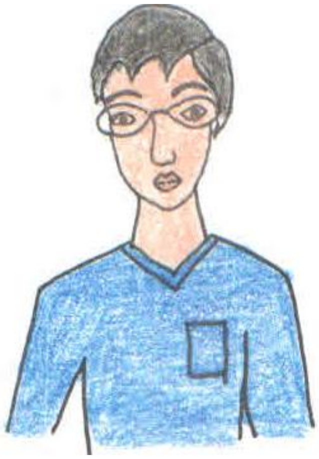

Tips when going through the worksheet:

- Try to use open ended questions; for example, "tell me about…".
- Validate by expressing why the young person might be experiencing something: for example, "it is natural you should have mixed emotions".
- Make sure you effectively listen and summarize what the young person is saying, for example: "is this what you mean?"
- Try to include motivational statements relating to the young person's recognition of the problem; any concern they might have about the effects (now and in the future) on family, friends, health; their current intention to

change, their level of optimism and past experience of self efficacy (i.e., when they have successfully effected changes in the past).

- Explore the young person's goals: for example, "what is important in your life?" and "how do your problems get in the way".

If the scores are below 5 but more than 2, this means that more motivational work might be useful. Explain to the young person that you will be doing some further work on motivation using more exercises and worksheets.

Worksheet: Are you ready to make changes

The fact that you are reading this, means that at least a small part of you is considering change. Any decision about change is not easy as it involves juggling mixed feelings. The following rulers may be able to help you.

Note for therapist: Please remember these ratings are based on young person's subjective evaluation or self report.

Importance of change:

Ask yourself the following questions. How important is it for you to change? What are your reasons and need for change? What score would you give yourself out of 10?

0 1 2 3 4 5 6 7 8 9 10

Ability to change:

Ask yourself the following questions. How confident are you in your ability to change? What score would you give yourself out of 10?

0 1 2 3 4 5 6 7 8 9 10

Once you have rated yourself on both rulers, think about the following questions?

- Why have you given yourself this score rather than a 0 or 10?
- What needs to happen to give you a higher score?
- What would you notice about yourself if you had a higher score?
- How would other people be able to help you to get a higher score?
- What strengths and supports do you have that would help you to get a higher score?

Do other people around you wants you to change? If yes, how much do other people close to you want you to change? Mark this on the line below

0 1 2 3 4 5 6 7 8 9 10

If there is a big difference between your own rating and that of other people, what does that tell you?

Exercise: Getting motivated

Start by letting the young person know that you will be working on their reasons why they are reluctant to give up self harm. Explain that it is very common to have mixed feelings about entering therapy to give up, in part because it can feel like an effective short term solution to seemingly intolerable stress. However, something has brought them to therapy; which implies a most important aspect of motivation or at least curiosity for change. Let them know that you are ready to work with them to make an informed choice and address any ambivalence or reluctance.

Explain that you will be exploring pros and cons of self harm. Use worksheet: Getting things into balance and discuss with the young person the balance sheet of Jamila.

**The pros and cons of self harm: Jamila** 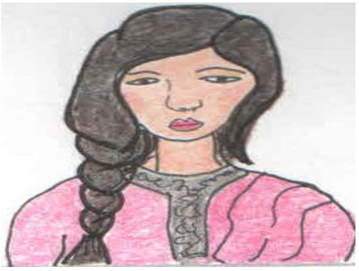	
Good things about my self for me now	***Not so good things about my self harm for me now***
It makes me calm and focused.<br>I like the sight of blood<br>It wipes away any bad feelings	It makes me ashamed that I have not been able to cope better and it hurts

Guide the young person that they should think about the positive and negative aspects of self harm.

Exercise: Looking at self harm through other people's eye (role play)

Use the **"worksheet: Looking at self harm through other people's eyes"**. The aim of this exercise is to enable the young person to explore how others view their self harm. This can be done through either role play or letter writing. See Sara's example of a role play exercise.

Worksheet: Looking at self harm through other people's eye

The aim of this task is for you to look at your self-harm through the eyes of other people. With your therapist, you are going to act out the following situations. Your therapist will pretend to be you each time and you will imagine you are . . .

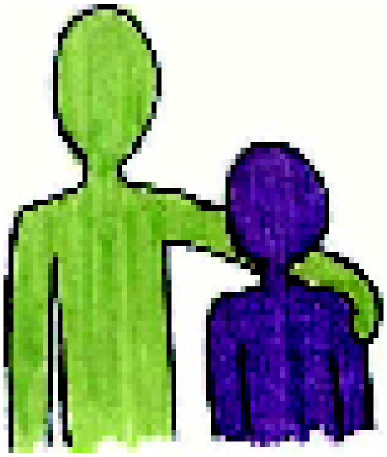

An adult figure like a teacher, family friend, favorite aunt or parent.
It should be someone you know will be fair and whom you respect. If you do not know someone like that, make him or her up. It could be someone from TV, a film, a book, or a character from history who has these qualities.
In a letter, write what you think this person would say to you about how they understand and see your self-harm. Explain the advice you think they would give you on how to change your life for the better (5 minutes).

A close and kind friend.
Imagine someone whom you deeply trust who will accept you, no matter what. Again if you can't think of anyone like that in your life, invent such a friend. How does your friend see you? How has your self-harm affected them? What are their thoughts and feelings? What advice do they have for you and your future?
Again, talk to your therapist as you imagine your friend would talk to you (5 minutes).

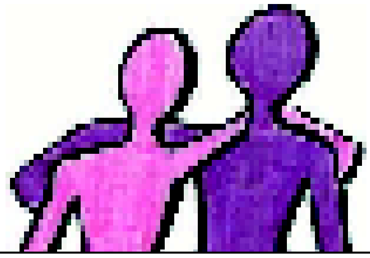

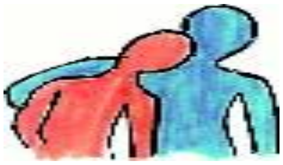

Yourself (when you are older and wiser) talking to yourself (now) about your self harm.
What would you tell yourself? Talk to your therapist in the way you would like the 'future you' to talk to you as you are now (5 minutes).

Reflection box: Spend the final 5 minutes thinking back over what you have talked about. Briefly write down what you've learned from this . . .

Role Play: Sara

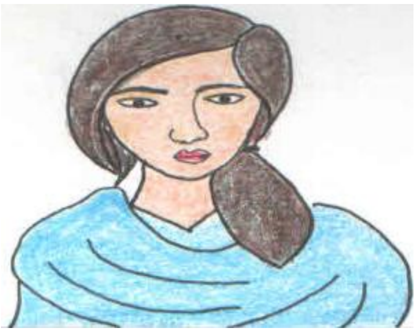

Sara and her therapist carried out a role play, so that Sara could gain insight into herself and others. Sara adopted the role of someone whom she respected and valued – her best friend Zainab – whilst the therapist assumed the role of Sara herself. The therapist then asked Sara/Zainab several open ended questions.

- **How do you see me?** You seem happy but there are times, when I know you are feeling sad and I want to be there for you.
- **How has it affected you?** I am concerned about you, but I am often scared to raise the topic of your self harm in case you get angry or defensive with me. When you go off by yourself I am always very worried about you and I would like to support you more if you would let me.
- **What are your thoughts and emotions?** I am frightened for you and I worry that one day you will go too far and inflict a severe injury upon yourself or worse.
- **What advice would you give me?** I would advise you to call me or someone else when you are feeling low and will try to distract you, listen to some uplifting music or watch your favorite TV program. I would advise you to relax and focus on something pleasant. If you have cut yourself, I would tell you not to feel ashamed or weak but to try to prevent it happening again.

In a role play, with the therapist pretending to be the young person each time, ask the young person to adopt three different personas in turn:

- **An adult figure**, such as teacher, a family friend, a favorite aunt or a parent. They should respect this person and know they will be fair. If they do

not know someone like that in real life, they should invent a person with those qualities, or use a character from TV, a film or a book. Once ‘in character’, they should be encouraged to talk to the therapist in that person’s voice about how they understand and see the young person’s self harm, including offering advice on how the young person might change their life for the better (5 minutes).

- **A close and kind friend** – someone who accepts the young person no matter what and whom they trust deeply. (Again if they can’t think of anyone like that, they should invent such a friend). How does this friend see them? How has the self harm affected them? What are their thoughts and emotions? What advice do they have for now and the future? Again the young person should be encouraged to answer these questions ‘in character’, as the friend (5 minutes).
- **An older and wiser version of the young person himself/herself.** What would they tell their younger self? What advice would they give? (5 minutes).

Exercise: Thinking about the Future

Use **“Worksheet: Thinking about the future”**. The aim of this exercise is to enable the young person to think about their future and the long term impact of self harm on their life. It is therefore another motivation exercise.

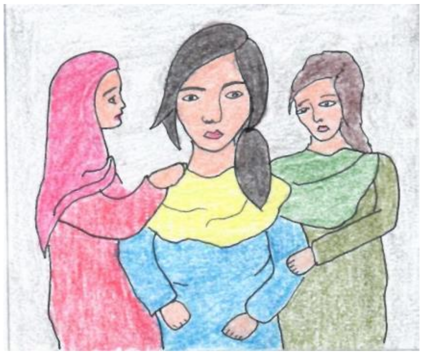

Thinking about the future: Kiran

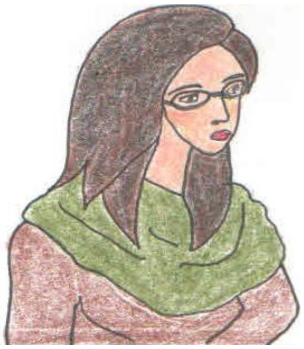

When the therapist asked Kiran to reflect on the longterm impact of her self harming behavior, she described, how she did not want her future husband or children to see her body covered in scars. She also feared that one day she might get a serious infection, accidently cut too deep or take too many pills. Keeping her self-harm a secret from her family resulted in feelings of shame and she often lashed out at her parents and siblings, fearing that they would discover what she was doing. As a result, Kiran felt growing separation between her and her family and she worried that she would become isolated from them in the future.

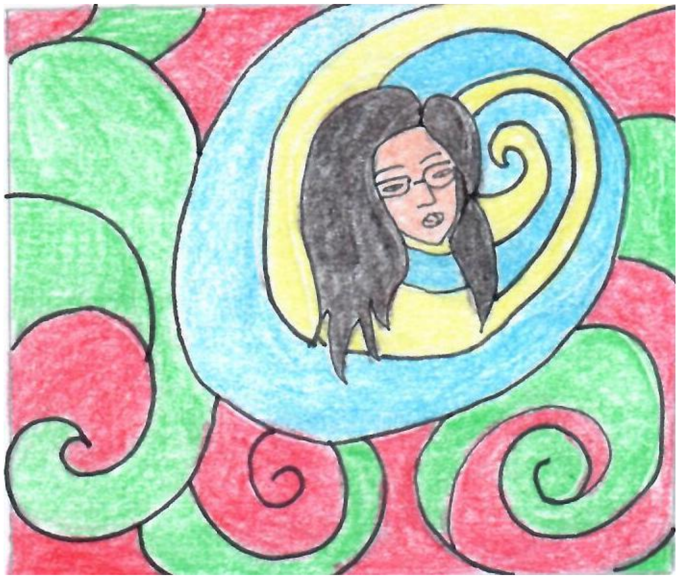

Session 5
Negative Automatic Thoughts (NATs)

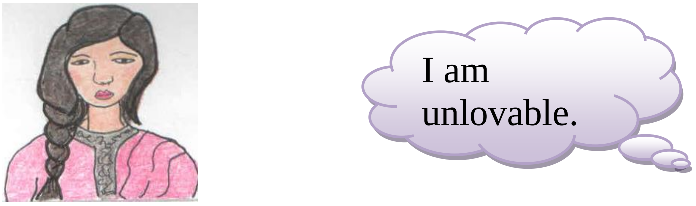

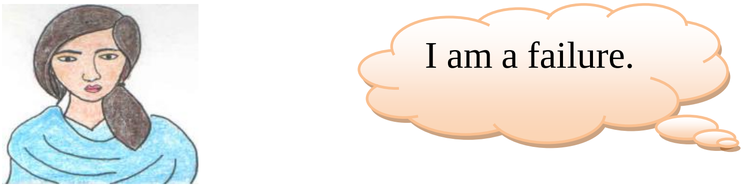

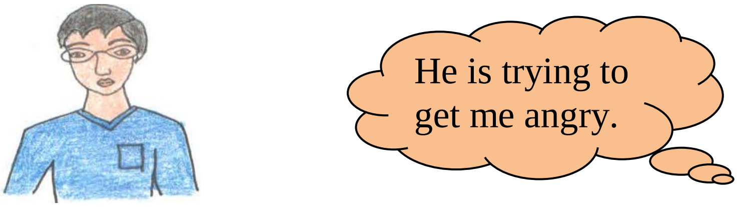

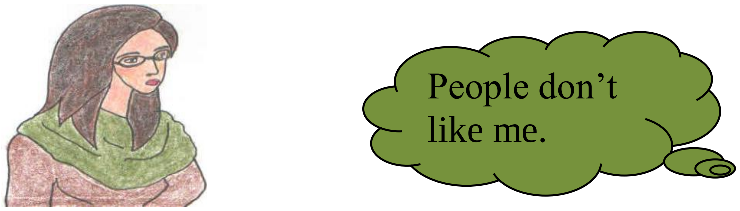

Aim:
The aim of this session is to become "thought detective" especially to understand what NATs are, and to recognize when they arise, their links with emotions and particular themes of NATs that the young person experiences.

Agenda:
1: Bridge from the last session
2: Homework review
3: Any issues raised by the young person
4: Main session topic
5: Homework plan
6: Feedback

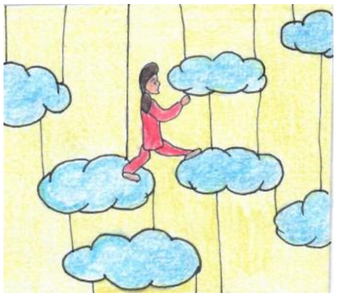

Main session topic:
Remind the young person of the central principle of Cognitive Behavior Therapy: that the way in which an individual behaves are determined by immediate situations(triggers), and the individual's interpretations of those situations. Explain to the young person that our minds are always busy. It can seem like there is a running commentary in our heads as we do things if we pay attention to our thoughts. We think about all sorts of things, what is happening around us, about ourselves and other people. These thoughts can be positive or negative (or both), but when we are feeling low or under-confident, they can be extremely negative and persistent. We all think like this from time to time especially when we are feeling low, depressed or anxious, the tendency is for our thinking to become extremely negatively biased(thinking error). At these times our thoughts are negative, habitual and quick, and therefore they can be difficult to identify. The fact that they are automatic and involuntary means they can also be hard to control.
Explain to the young person that NATS are due to biases in the way we process information. This means that we are seeing the world through a type of "negative filter".
These thoughts may be about the way we see ourselves:

I am fat.
I have lots of friends.
I am very moody.
People think, I am funny.

They may be about how we judge and comment on what we do:

I am no good at revision.
I am quite sociable.
I am good at listening to other people's problems.

They may describe our view of the future:

No one will ever want to go out with me.
I will fail my exams.
I will be a professional cricketer.

A key part of trying to manage our feelings is to become a sort of **"thought detective"** and to start to recognize the thoughts that appear when we feel strong emotions. In order to feel better we need to recognize our thoughts and then try to disentangle helpful and unhelpful thoughts and question the validity of some of the repetitive negative thoughts.
At this stage you can go back to **"worksheet: what is CBT"** and use that to discuss the link between thoughts and feelings, as appropriate. Show the young person Sara's NATs about herself, the future and others and discuss them, then complete the exercise below to help them identify some their own NATs.

Some of Sara's negative thoughts about herself, the future and others

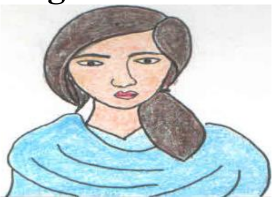

I am not good at school work.
I am going to fail my exams.
No one cares about or loves me.

For this exercise, read through the outline problem scenario and think together about what the emotional, physical and behavioral reactions the young person might have. Following this, think about what automatic thoughts the young person has that link with the reactions they have given.

Problem:
You are sitting at school, eating lunch with a friend when one of your teachers walks by and says to you in a irritated tone, "please come and see me in my office as soon as you have finished your lunch."
What reactions you might have?

- Emotional (e.g. afraid, anxious)
- Physical (e.g.increased heart beat, sweating)
- Behavioral (e.g. avoidance)

What automatic thoughts you might have?

When you feel that the young person can identify NATs, either work through another blank help triangle, or use a recent event and ask them to identify the NATs from their thoughts. Again highlight the thoughts, emotions and behaviors in the appropriate sections.

The next step is to consolidate this skill and continue completing real life examples. Depending on time, you can either continue with examples during the session or set this as homework task.

Continue to practice using help triangle. Take everyday examples of feeling surges or switches and fill in more triangles during the week.

Thought distortions

Explain to the young person that now that they have some experience in identifying their thoughts, highlighting their NATs and recognizing the impact negative thoughts can have on emotions and behaviors, we are going to look at ways to break this cycle and challenge the NATs. Look at their help triangle. Continue to identify and pick out themes of thoughts that keep arising by asking, 'Can you see any thoughts/types of thinking that come up again and again?' Tell the young person that we sometimes muddle up thoughts with facts and might accept them without question.

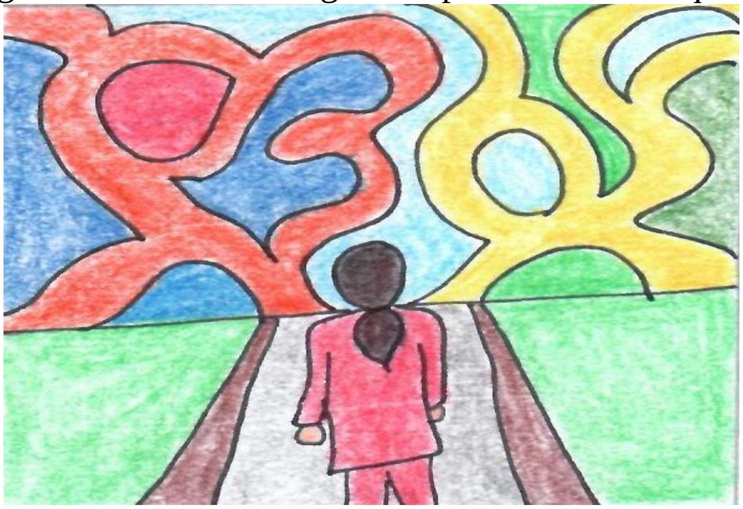

Explain that the expression **'looking through rose-tinted spectacles'** is used to describe someone who always sees things in a hopeful or cheerful way, even when they are bad. This is a kind of thought distortion or thinking bias because

it is unrealistic. The person sees the situation from only one viewpoint and does not see the negative aspects of it. Conversely, someone else might see things only from a negative perspective: 'My friend never rang. Nobody ever wants to talk or listen to me.' Thought distortions or thinking biases are common and unhelpful ways of thinking. Everyone makes these distortions, but when they happen regularly, they can make you feel bad, and affect your decisions about things and how you behave. A useful strategy is to learn, to analyze these thoughts and challenge them, both to test out if they are true or not and to help you feel better. There are many different types of thinking bias, which you should now discuss further. The five distortions listed below are the main ones to look out for, although there are many others, too.

Exercise: Thought Distortion

Use **“worksheet: Glasses”** and **“worksheet: Thinking pitfalls”** to illustrate and prompt discussion of the five main distortions. Go through each example in turn, highlighting the glasses analogy. For example, in 'black-and-white' thinking, the person has the black-and-white glasses and interpret situations in an 'all-or-nothing' way (see Kiran's example).

Black and White thinking: Kiran

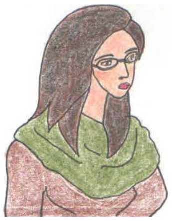

Kiran had an argument with a good friend and thought, 'That's it, I'm not friend with her any more. I'm never going to speak to her again.'

Note: It is not necessary to explain all thinking errors in one session.

<u>Black-and-white thinking</u>: Looking at things in an 'all-or-nothing' way: for example, someone who sees things as either wonderful or terrible, total success or complete failure, with nothing in between.

<u>Jumping to conclusions:</u> When you conclude that things are going to go wrong, or you have done something wrong, without considering possible alternative explanations. Thinking that you know how someone else thinks or feels (`mind-reading') or thinking that you know what will happen ('fortune-telling'):

For example,

Not sitting a test because you 'know' you will fail or thinking that someone no longer likes you because they did not say hello.

<u>Over-generalising:</u> Blowing things out of proportion. You can often spot these when there is an 'always', 'never', 'everyone' or 'no one' in the thought:

For example,

Getting a bad grade and thinking, *'Everyone* else is better than me. I am *never* any good at *anything*.'

<u>Should/must/ought</u>: Giving yourself a hard time:

'I must do better', 'I should be better', 'I ought to have known better'

As you look at the various thinking pitfalls, get the young person to think about a time when they might have fallen into each particular thinking trap. Use their help triangles to find their personal thinking pitfalls.

Tell the young person not to worry if all of the above sound familiar. Explain that these are all thinking biases that everyone makes. You may find that you do one more than the others or that you combine several types of thinking bias.

Note for therapist: It will be helpful enough if the participant can identify his/her thoughts as an unhealthy/thinking bias and not able to identify type of thinking error.

Thought-challenging

Thinking biases can make us experience some unpleasant feelings, such as sadness, anger, etc. Sometimes we are so self-critical that we start to accept these thoughts as true facts. Checking these thoughts out, and challenging them to test whether they are true, can help to stop them going round and round in our heads. If we don't challenge them, we can end up feeling worse.

Remind the young person, again that what we think affects, how we feel and what we do. Explain that challenging our thoughts is more than just thinking positively about life; it is about having *balanced thinking,* which involves looking for evidence to *support and challenge* our thoughts.

Exercise: Thought-challenging

Consult the feelings diary the young person completed and/or one of the help triangles they did or find a new example. Highlight one negative automatic thought, the more extreme the better: for example, 'I'm useless at everything.' Be careful to choose a thought that is about the young person, as these thoughts tend to be more powerful and prone to distortions based on young people's beliefs about themselves, others and the world. It is not a good idea to try to challenge a thought about another person at

this stage (as we don't know the whole story), so avoid thoughts such as: 'Zainab doesn't like me.'

Evidence for – evidence against:
When you have identified a thought, explain to the young person that you will be looking for evidence for and against it. Make two columns on a piece of paper, with one side evidence for and the other side evidence against. Ask the young person to identify all the evidence for the thought being true by explaining the reasons why they believe that thought to be true. Really try to stretch the young person to tell you *every* reason - including such nebulous concepts as having a 'feeling' - as well as their previous experiences linked with the thought. The aim is to get everything down, now, to avoid later 'yes . . . buts'. It is important that this stage is done thoroughly so that the young person has a chance to say why this thought keeps recurring. Although they may feel like they are re-experiencing the unwelcome emotion, it can be very validating to hear it in this way.

In the second column (evidence against), instruct the young person to look for and record any evidence that challenges their thought. Tell them that they are now thinking of evidence that goes against the specific thought: for example, the idea that they are 'useless at everything'. This is often the hardest part of the exercise and may require more prompting. Ask what alternative explanations there might be, ask them to think what someone else (who knows the young person) might suggest as evidence against this thought.

After they have identified all the evidence against, spend some time reading both columns back to the young person. Ask them what they make of the exercise and what they are thinking now. Can they re-rate their belief in this thought now? Then ask if they can identify a more balanced thought or alternative - less negative - interpretation of the same thought based on this exercise. This is also an opportunity (if they have not done so earlier) to identify which type of thinking bias(es) this thought might be.

Exercise: The thought record
Use **"worksheet: The thought record"** and explain that this is a more advanced version of the help triangle. It is divided into ten columns, with key symbols for most columns. The advancement from the help triangle is in the strategy of challenging the NAT, as you have just done in the previous exercise. After looking at the instructions and Kiran's example, start to fill in the columns one by one, using the earlier example.

Explain to the young person that the thought record should be used when they next experience an unpleasant mood or troubling thought. As you fill it in, discuss each column in turn.

Situation. Where were you? What were you doing? When? With whom?

Feelings. Use one word to describe each of your feelings at the time. Rate how strong each was using the feelings scale (0-10).

Thoughts. Describe any thoughts you noticed going through your mind. Highlight at least one NAT from the example and write it in the column.

Beliefs. Next, ask the young person to rate how much they believe that thought as a percentage (100 percent = believe it to be true without any doubt; 0 percent = believe it is totally untrue). The young person may describe believing it 100 percent at the time but now being less sure (for example, 80 percent).

Note for therapist: you can also use 0-10 scale for rating belief

Balancing. The next step is for the young person to record evidence that they can see supports their thoughts. After they have identified all the evidence against, ask them to work on the steps 6-9.

Thinking distortions/bias. Looking back at their NATs, can the young person spot any thinking biases/exaggerations in their thinking?

Beliefs again. Now the young person should re-rate the percentage of their belief in each thought. Have these changed?

Alternative thoughts. Now that the young person is able to perceive their thought in a more balanced way, they should try to come up with an alternative - more carefully considered or balanced - thought.

Note for therapist: These alternative thoughts could be put into a flashcard that the young person could carry with them at all times to deal with crises

Feelings again. Finally, the young person should think about whether their feelings have changed and record this.

Table: Sample Thought Record – Kiran's Situation

Situation	What were your feelings?<br><br>Scale rating<br>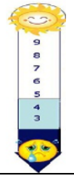	What thoughts were going through your mind? (NATs)	How much do you believe thoughts?	What evidence supports your beliefs in these thoughts?	What challenges your beliefs?	Can you spot thinking pitfalls?<br>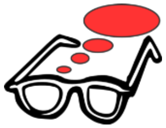	How much do you belief thoughts now?	Possible alternative thoughts?<br>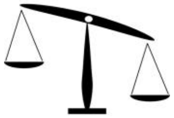	How do do rate feelings now?<br>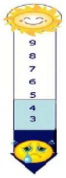
Argument with my best friend	Angry (100%) hurt (90%) cheated (75%)	I am unvalued.<br><br>I have never meant anything to her.<br><br>I am pathetic.	100%<br><br>80%<br><br>60%	She often makes me feel worthless.<br><br>She seems to pick fights, to upset me and break our friendship.	We are both strong personalities with different ideas.<br><br>Afterwards, we can laugh about the disagreement.	Over-generalizing	60%	It is OK to disagree.<br><br>I can still be important to someone even if we argue.	Angry (40%)<br><br>Hurt (60%)<br><br>Cheated (30%)

Homework:

- Help triangle.
- Young person should also complete thought record form.
- Also give handout for session 5.

Handout for Young Person: Session 5

Thought distortions:

Black-and-white thinking: Looking at things in an 'all-or-nothing' way: for example, someone who sees things as either wonderful or terrible, total success or complete failure, with nothing in between.

Jumping to conclusions: When you conclude that things are going to go wrong, or you have done something wrong, without considering possible alternative explanations. Thinking that you know how someone else thinks or feels (`mind-reading') or thinking that you know what will happen ('fortune-telling'):

For example,

Not sitting a test because you 'know' you will fail or thinking that someone no longer likes you because they did not say hello.

Over-generalising: Blowing things out of proportion. You can often spot these when there is an 'always', 'never', 'everyone' or 'no one' in the thought:

For example,

Getting a bad grade and thinking, *'Everyone* else is better than me. I am *never* any good at *anything.'*

Should/must/ought: Giving yourself a hard time:

'I must do better', 'I should be better', 'I ought to have known better'.

These are often linked with over-generalizations: 'I should always', etc.

Blaming yourself: When you feel responsible for things that are not your fault or that are beyond your control:

For example,

'My dad left because of my behavior', 'It is my fault that I got beaten up', etc.

Completing thought record:

Situation: Where were you? What were you doing? When? With whom?

Feelings. Use one word to describe each of your feelings at the time. Rate how strong each was, using the feelings scale (0-10).

Thoughts: Describe any thoughts you noticed going through your mind. Highlight at least one NAT from the example and write it in the column.

Beliefs: Next, ask the young person to rate how much they believe that though is in a percentage (100 percent = believe it to be true without any doubt; 0 percent = believe it is totally untrue). The young person may describe believing it 100 percent at the time but now being less sure (for example, 80 percent).

Balancing: The next step is for the young person to record evidence that they can see which supports their thoughts. After they have identified all the evidence against, ask them to work on the steps 6-9.

Thinking distortions/biases: Looking back at their NATs, can the young person spot any thinking biases/exaggerations in their thinking?

Beliefs again. Now the young person should re-rate the percentage of their belief in each thought. Have these changed?

Alternative thoughts*:* Now that the young person is able to perceive their thought in a more balanced way, they should try to come up with an alternative - more carefully considered or balanced - thought.

Feelings again*:* Finally, the young person should think about whether their feelings have changed.

Worksheet: Glasses

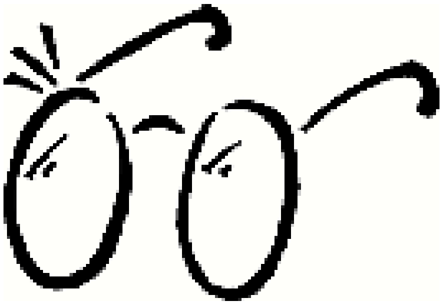

1 + 2 = 5

Jumping to conclusions

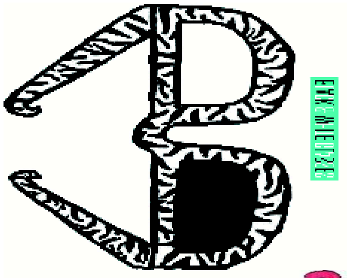

Black and White thinking

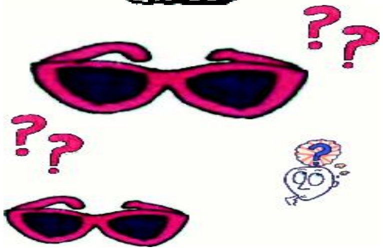

Over-generalization

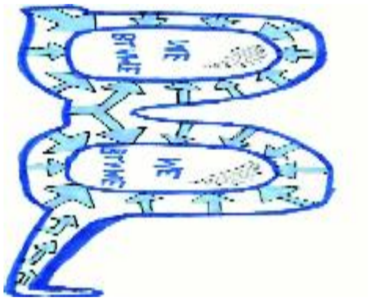

Blame Yourself Glasses

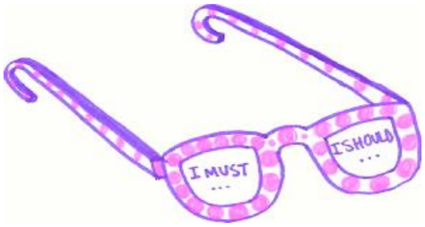

I Should/Must/Ought Glasses

Worksheet: Thinking Pitfalls

You may have heard the expression ‘looking through rose-tinted spectacles’ to describe someone who always sees things in a hopeful or cheerful way even when things are bad. This is a kind of thinking pitfall because it is unrealistic.
The person sees the situation from only one viewpoint and does not see the negative aspects of the situation.

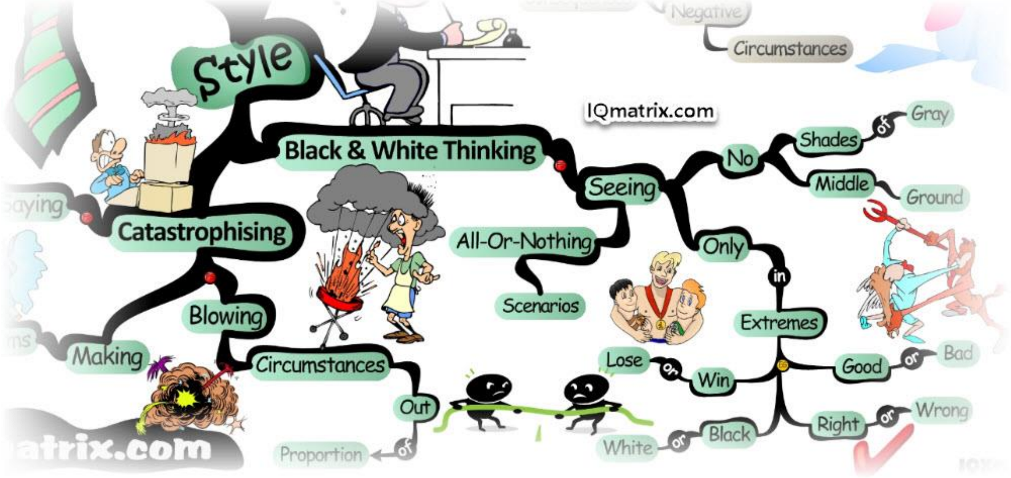

Thinking pitfalls or biases are unhelpful ways of thinking. Everyone makes these pitfalls, but when they happen regularly, they can make you feel bad and affect your behaviour.

Black-and-white thinking: Looking at things in an ‘all-or-nothing’ way.
Jumping to conclusions: Thinking that you know how someone else thinks or feels (‘mind reading’) or thinking that you know what will happen (‘fortune-telling’).

Over-generalising: Blowing things out of proportion, you can often spot these when there is an ‘always’, ‘never’, ‘everyone’ or ‘no one’ in the thought

Should/must/ought: Giving yourself a hard time! ‘I must do better’, ‘I should be better’, ‘I ought to have known better’.

Blaming yourself: When you feel responsible for things that are not your fault or that are beyond your control: ‘My dad left because of my behaviour’
Don’t worry if all of the above sound familiar to you! They are all pitfalls that everyone makes. You may find that you do one more than the others or that you combine different types of thinking pitfalls.

Worksheet: Sample Thought Record

Situation	What were your feelings? Scale rating 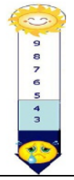	What thoughts were going through your mind? (NATs)	How much do you believe thoughts?	What evidence supports your beliefs in these thoughts?	What challenges your beliefs?	Can you spot thinking pitfalls? 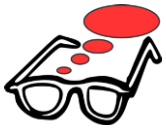	How much do you belief thoughts now?	Possible alternative thoughts? 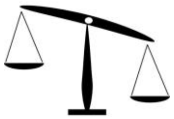	How do do rate feelings now? 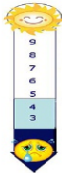

Session No. 6
Core Beliefs

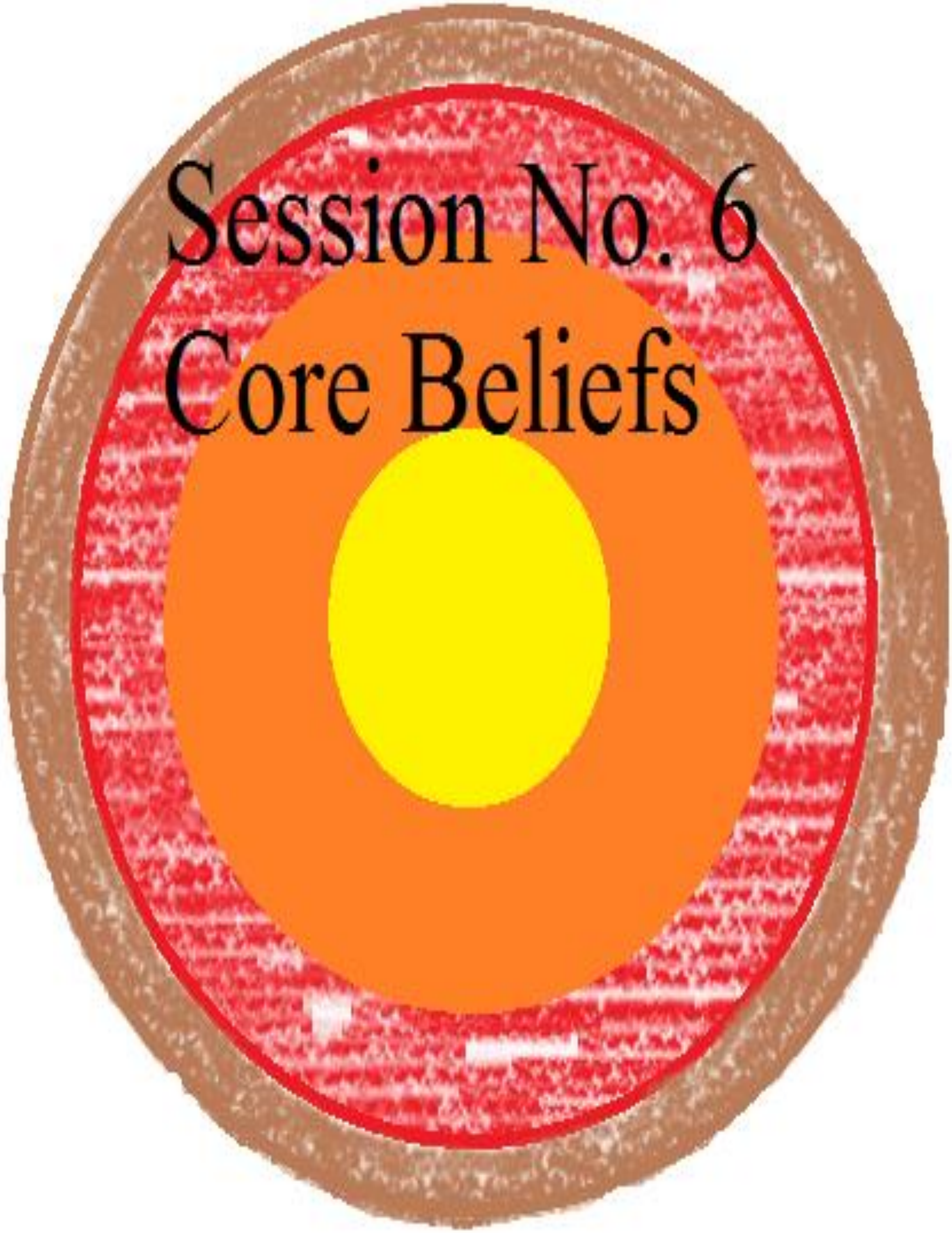

Aim:
If the young person is more insightful, very depressed or older, with well- established patterns of behavior and recurring NATs, it can be useful to identify their core beliefs.

Agenda:
1: Bridge from last the session
2: Homework review – Thought Record Form
3: Any issues raised by the young person
4: Main session topic
5: Homework plan
6: Feedback

Note for therapist: CBT triangle used to explain them connection between thoughts, feeling and behavior that will further help them to relate it with core beliefs.

Example:
I am not good enough
The belief that 'I'm not good enough', which may arise from living in a home where achievement is valued over and above anything else (or is perceived to be), may lead a person to develop the rule that they will be successful only if they do everything really well or even perfectly. This might result in stress and unhappiness as each piece of work is repeated over and over again or perhaps never even started.
This is an example of how a core belief - 'I'm not good enough' - can trigger an automatic thought and an assumption - for example, 'I'm stupid' - and create a rule of living: 'there's no point in even starting my schoolwork'

Main session topic:
Explain the young person that there are three different levels of interpretation when anyone encounters a situation. First, there are the automatic thoughts (discussed in the last session), which usually appear as verbal statements (or images) in our heads. The second level includes 'rules for living' or conditional assumptions. These are less obvious than automatic thoughts and we can figure them out only by looking closely at our actions. Look at Kiran's example.

Conditional Assumptions: Kiran's Example

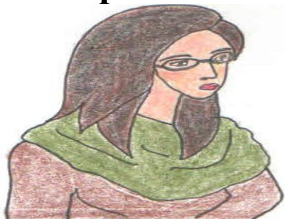

'A girl in the year above me disagreed with my opinion during the discussion we had after school. I immediately felt bad and thought, "They don't like me and they think I'm stupid."' In this case, Kiran's assumption might be either:

- *if* people disagree with me, *then* they don't like me

or

- other people's opinions are more important than mine

Assumptions often occur as 'if ... then' statements and sound like general rules. This will make more sense later, after core beliefs are explained. Ask the young person to hold on to this as a concept but reassure them that you will be explaining in more detail after focusing on core beliefs — the third and deepest level of cognition.(flash cards can be used)

Core beliefs:
Core beliefs are seemingly unquestionable opinions about;

- Ourselves
- Others
- World

Explain to the young person that when we are growing up, we develop these beliefs on the basis of both our actual experiences and our perception of our experiences. The patterns of thinking that develop, which are seen as rules and beliefs about the world around them, are not necessarily reflections of the 'true' environment but instead the consequence of an underdeveloped mental ability in the young child that is rather rigid and inflexible. As we grow up, most of the 'rules' we have developed during our childhood become more fluid and flexible as we see exceptions and alternative explanations. However, some of our childhood beliefs stay absolute even into adulthood.

When we think the same thoughts about ourselves over and over again, and

repetitively judge what we do, those thoughts and beliefs grow stronger and more fixed. As well as this, when early life experiences recur or are extremely traumatic, we can become convinced that this will continue to happen. This perception can start to rule our lives because the thoughts are often extremely negative and become too strong and plausible to challenge.

The second reinforcer of such beliefs is the information-processing bias that follows. In other words, any new evidence that contradicts the beliefs tends to be ignored or dismissed as unimportant and untrue, as it does not fit with our perception of reality; essentially, it is filtered out.

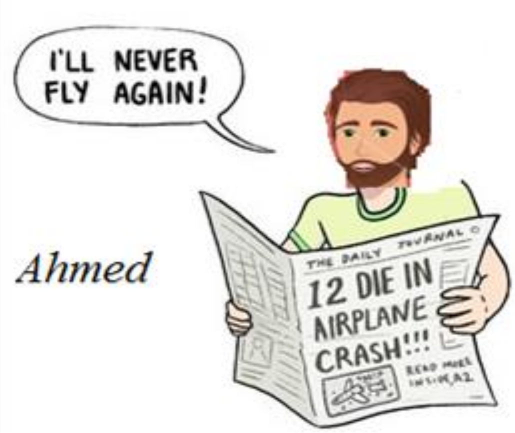

Core beliefs tend not to be in our heads all the time (or ever). Rather, they seem to be held below consciousness and manifest only occasionally as short, brief statements: for example, 'I am lovable' or 'I am worthless'. If beliefs are negative — for example, `I'm a failure' — they often make you feel bad and can filter the way you see the world.

Part of the information-processing bias is that anything that supports the negative core belief, however trivial, is seized upon as 'proof' of its veracity. As well as this, because core beliefs help us to make sense of our world at a young age, it may never occur to us to evaluate whether there are more useful ways to understand our current experiences. Instead, as adolescents (and into adulthood), a person might act, think and feel as if these beliefs are 100 percent true.

Sara's Core Beliefs

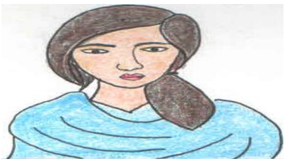

"Everything I do must be perfect"
"I always get things wrong — I'm a failure"
"I am unlovable"

Exercise: Core beliefs

Look at Sara's core beliefs and ask the young person which dangers/ difficulties she might experience by holding on to them. (For example, by believing that everything she does must be perfect, she might avoid trying new things because of a fear of failure; or she might judge herself harshly whenever she doesn't obtain a top grade at school).

Exercise: Rules of Living

Remind the young person that core beliefs are so entrenched that they can influence the decisions we make. This means that they can lead us to develop certain rules and patterns of living. Go through two examples: 'I'm not good enough' and 'I'm unlovable and no one will ever love me'.

I am not good enough

The belief that 'I'm not good enough', which may arise from living in a home where achievement is valued over and above anything else (or is perceived to be), may lead a person to develop the rule that they will be successful only if they do everything really well or even perfectly. This might result in stress and unhappiness as each piece of work is repeated over and over again or perhaps never even started.
This is an example of how a core belief - 'I'm not good enough' - can trigger an automatic thought and an assumption - for example, 'I'm stupid' - and create a rule of living: 'there's no point in even starting my schoolwork'

The rules of living brought about by Sara's core beliefs

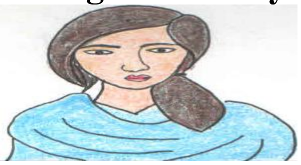

"There's no point in even starting my assignment as it won't be good enough (done perfectly)"

"People will like me only if I do what they want me to do."

"If I'm successful, then I'll be happy."

I am unlovable and no one will ever love me

The belief that `I'm unlovable and no one will ever love me' might lead a person to assume that others don't really want to spend time with them, so they will probably feel sad and avoid other people. Avoiding other people means that they have no chance to experience others showing that they like them and want to be around them. This in turn could lead to others thinking that the person doesn't want to be friends with them, so they might start to avoid them too. This strengthens the person's conviction that their belief is true.

In this example, the core belief 'I'm unlovable' has led to the assumption 'people don't want to be around me' and caused the rule of living 'avoid other people, as they don't want to be around me'. In turn, this has strengthened the core belief.

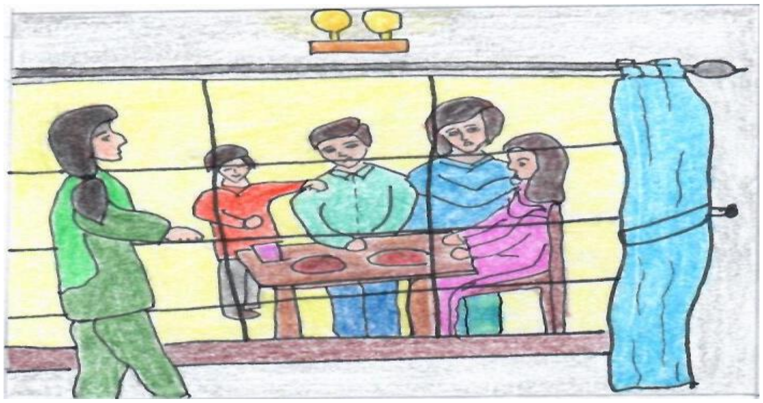

Exercise: The pen-friend, or new Facebook friend

Use the **"worksheet: The new Facebook friend"** and explain to the young person that you are going to try to identify their core beliefs and rules of living. Ask them to imagine that they are writing to a new facebook friend, someone they have never met, so they need to provide a description of themselves. Explain that the idea is to think 'off the cuff': there is no right or wrong answer and the description should be based on 'gut feeling'.

On the worksheet, use the following prompts and then fill in the details (which can be discussed together):

- I am . . .
- Other people see me as .. .
- Other people are .. .
- Relationships are .. .
- The world is . . .
- The future is .. .

Pull together the previously completed thought records and help triangles and ask

the young person to think about the themes that come up for them. What do they think their core beliefs might be and what are their associated rules for living.

Worksheet: The Facebook friend

Imagine you are writing to a new facebook friend, someone who has never met you, and you are describing yourself.

Use the following prompts to fill in the details (can be discussed with your therapist).

- I am . . .
- Other people see me as .. .
- Other people are .. .
- Relationships are .. .
- The world is . . .
- The future is .. .

**My core beliefs are:**<br><br>I am unloveable

**My core beliefs lead to these rules of living:**<br><br>"People will like me only if I do what they want me to do."

Formulation – My journey

To piece together why problems have started, what maintains them and where they might have originated. This is the cognitive behavioral formulation. It's a bit like making a map of their life. Explain that it can sometimes feel uncomfortable to revisit the past because it can dredge up unpleasant memories, but it can also be a very helpful process to help us understand what makes them unique, to identify their `danger spots', and to stop them from falling into them again and again.

Start by saying that there is increasing evidence that what we experience in our early lives (maybe even from the time when we are babies) is programmed into our minds and leads us to have certain beliefs about ourselves, the world, other people and the future. These implanted beliefs help us to process information efficiently and allow us to make sense of a rapidly changing world. When we are very young, this is quite a good thing, as, for example, it would be very tiring (and a very inefficient use of our brains) if every time we looked at a chair we had to relearn what it was and what it was used for.

However, if, for example, a young child feels scared when they are near certain people, they might start to believe that ‘other people are dangerous’ and this belief might then start to dictate their behavior. Alternatively, if they start to believe that they are being compared unfavorably with a sibling who has different skills, they might start to believe that ‘other people are more successful than me' or ‘I'm no good'.

Thus, core beliefs are established when we are very young and continue to be established and strengthened as we grow up, and these can lead to certain rules of living that we follow, sometimes without even knowing that we are doing it. Sometimes, a specific event might 'activate' a core belief: for example, a person who feels 'unlovable' might become depressed when a relationship breaks down. This, in turn, will lead to an upsurge of NATs and behaviours that reinforce the core belief.

To illustrate this process, look at Jamila's formulation (Worksheet: Jamila's formulation).

Jamila’s Formulation:

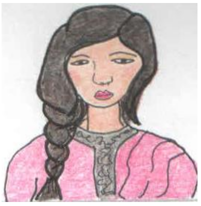

Why me?

- Early experiences: Grandmother died; parents divorced and difficult relationships with Dad; sister was the 'golden child'; I was bullied at school; Mum was depressed; at home there was lack of expression of emotion and no arguments.
- My beliefs and rules of living (based on pen-friend exercise and early experiences): I'm not good enough; I'm unlovable; other people's needs always come first; if people disagree with me, then they don't like me, or their opinions are more important than mine.

Why now?

What happened before (my problems got really bad)?

- Mum got admitted in hospital

Why still:

What keeps my problems going?

- Avoid going out.
- Don't get close to anyone.
- Don't tell people how I'm feeling.

Good stuff

What helps?

- Listening to music.
- Talking to a Cousin.
- Forcing myself to go to friend's birthday.
- My positive self-statements and challenges to my crucial thoughts.

Exercise: My Journey

Use the **“worksheet: My journey”** and start to fill in the young person's own formulation. Explain that there are no wrong or right answers and sometimes they will have to guess. This is just the start of the process and it will evolve over time, as we look at the young person's 'journey' in more detail over the next few sessions. The journey proceeds from the young person's early experiences, to their core beliefs and rules of living, to 'Why now?' and 'Why still?', before concluding with the 'Good stuff' that helps them to manage their current life.

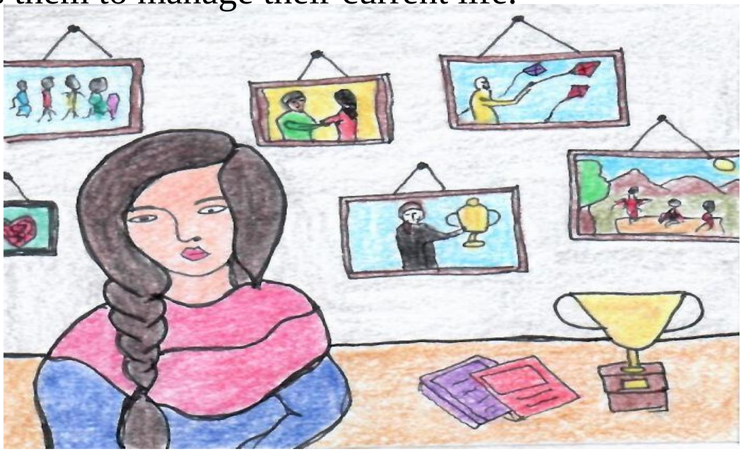

Tell the young person that they should begin with their early experiences. This is the moment when everything they have told you previously about their childhood should be written down. They should include significant life events, parental break-ups, mental health problems in the family, bullying, etc. Useful prompt questions include:

- Can you think of any happy and sad memories from your childhood?
- What was the atmosphere like at home when you were younger?
- What is your mum/dad/sibling/carer like? Can you describe them?
- What were you like as a younger child?
- How was school? What were your parents' views on academic success, homework, etc.?
- What were the rules like at home? Was there much discipline?
- Did people show their emotions or hide them?

It can be useful to start by writing in key issues that the young person feels are highly relevant to their story. Ask if they feel that certain events/ issues in their life are particularly relevant. They might reflect on their childhood and remember specific incidents, but might not know if these are relevant to their formulation at this stage. This is a learning phase for both the therapist and the young person, so it is fine to

write some ideas in pencil or outside the boxes if you cannot find obvious places for them within the formulation.

Next you should move on to the beliefs section. Ask the young person: `What kind of beliefs might someone who has experienced the things in your early experiences, beliefs about the world, others and himself/ herself?' One way of accessing the young person's core beliefs and rules for living is to look through their thought record. Looking at recurring themes that arise might help you to come up with core beliefs and rules of living collaboratively. Another way is to use the **'downward arrow'** technique. For this, you take one of the young person's negative thoughts and ask them, 'What does this situation mean or say about you?' and `If this were true, what would be so bad about that?'. The question `What does this mean or say about you?' should then be repeated after each of the young person's answers (a bit like peeling away the layers of an onion), until you arrive at their core belief. For two examples of this process, use **“worksheet: Ahmed’s core beliefs”** and **“worksheet: sara’s core beliefs”.**

Move on to the 'Why now?' section and ask the young person what happened before the problems started to get really bad. Was there a particular trigger? Why did things slip into the way they are now?

Once you have completed 'Why now?' go on to 'Why still?' This section looks at maintaining factors. It can be completed once the young person starts to gain an insight into why their self-harm has developed into a cycle. The idea is to understand the behaviors and responses from others that maintain the problems.

The exercise concludes with the 'Good stuff'. In this section, the aim is to identify all of the positive things with which the young person is engaging.

Worksheet: My Journey

Why me?
difficult relationships with Dad; brother was the 'golden child'; I was bullied at home; Mum also supporting brother; at home there was lack of expression of emotion and everyone fights

Why now?
Father stopped me to study further

Why still?
My other friends going to school
Family member started looking for my proposal

Good stuff?
Started stiching at home
Going on work along mother

Worksheet: Ahmed’s Core Belief

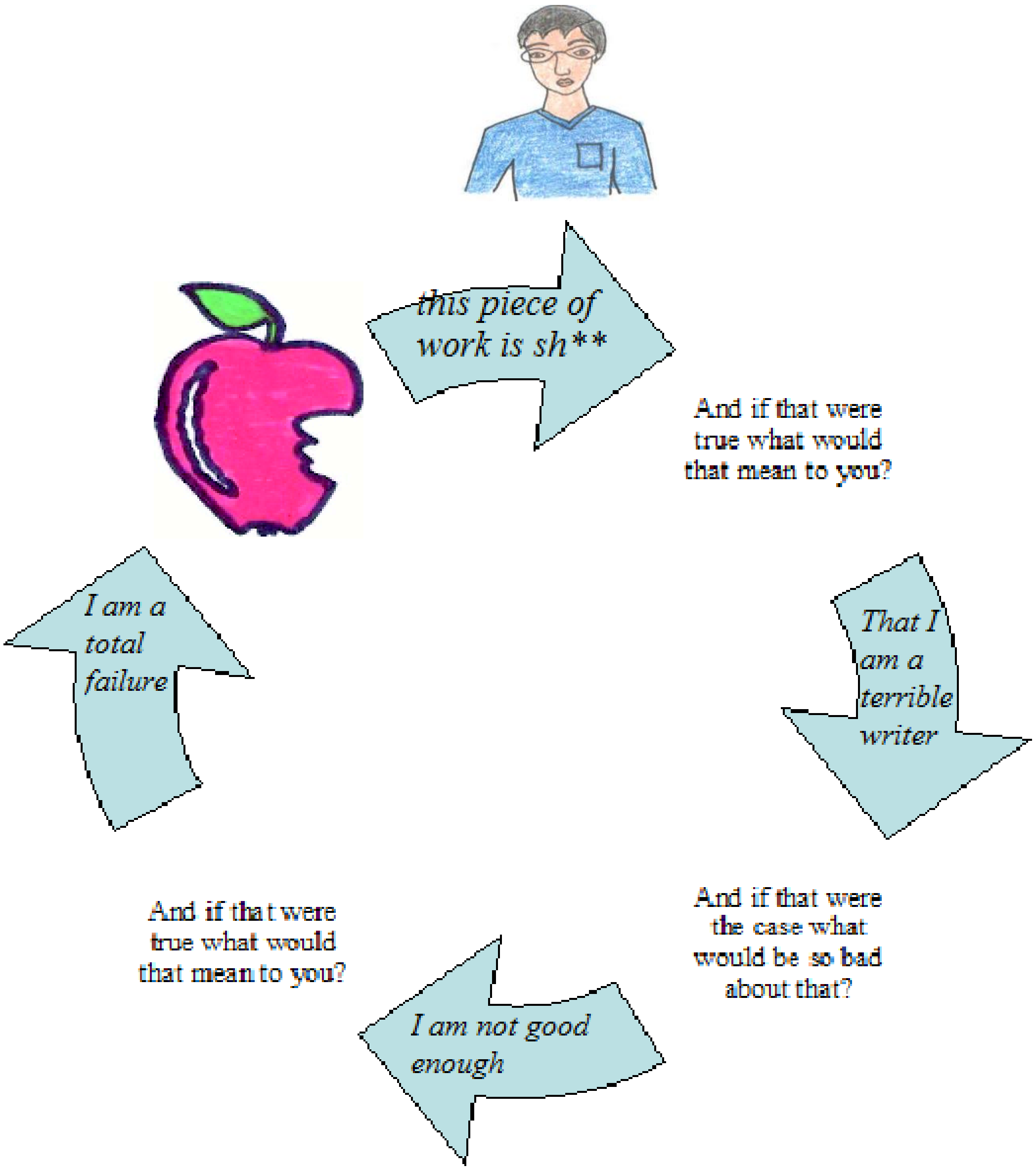

Worksheet: Sara's Core Belief

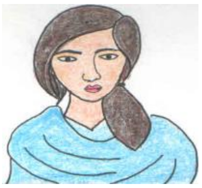

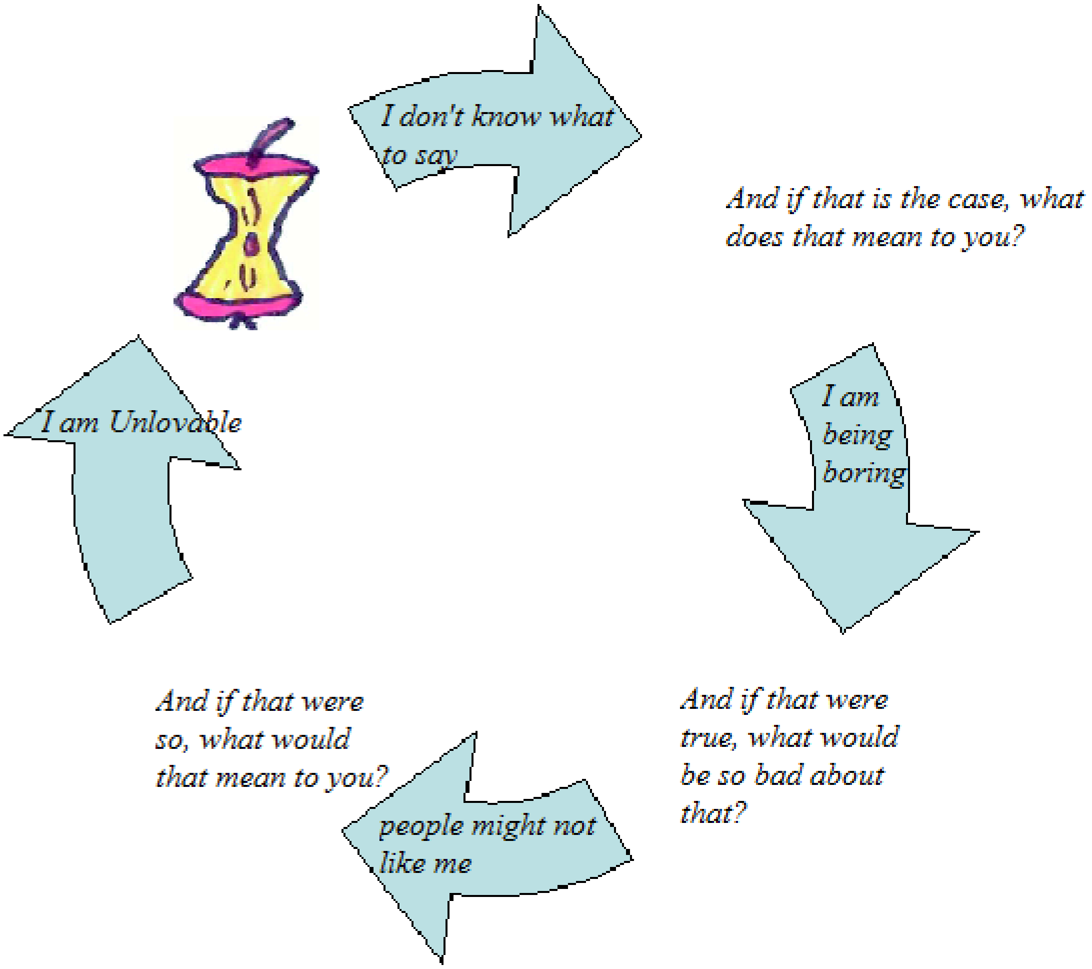

Homework Assignment:

- Continue with thought record form and the identification of core beliefs.
- Give handout for session 6.

Handout for the young person: Session 6

Formulation

The formulation is how your 'story' fits together. In the previous sessions, you have focused on the individual parts that make up the formulation: the core beliefs, NATs and the rules for living. The aim of formulation is to piece together how you have ended up being the person you are, and why you experience the world in the way that you do. It's a bit like making a map of your life. It can sometimes feel a bit uncomfortable to go back to the past and it can dredge up unpleasant memories, but it can also be a very helpful process to help us understand what makes you unique, to identify your 'danger spots' and to stop you falling into them over and over again.

The formulation comes from the theory that what we experience in our early lives (maybe even from when we are babies) is programmed into our minds and leads us to have certain beliefs about ourselves, the world, other people and the future. These implanted beliefs are designed to help us process information efficiently and make sense of a rapidly changing world. This is quite a good thing, as, for example, it would be very tiring (and a very inefficient use of our brains) if every time we looked at a chair, we had to relearn what it was and what it was used for.

However, if, for example, a young child feels scared when they are near certain people, they might start to believe that 'other people are dangerous', and this belief might then start to dictate their behaviour. Alternatively, if they start to believe that they are being compared unfavourably with a sibling who has different skills, they might start to believe that 'other people are more successful than me' or 'I'm no good'.

Thus, core beliefs are established when we are very young and continue to be established and strengthened as we grow up, and these can lead to certain rules of living that we follow, sometimes without even knowing that we are doing it. Sometimes, a specific event might 'activate' a core belief: for example, a person who feels 'unlovable' might become depressed when a relationship breaks down. This, in turn, will lead to an upsurge of NATs and behaviours that reinforce the core belief

Session 7

Coping Strategies

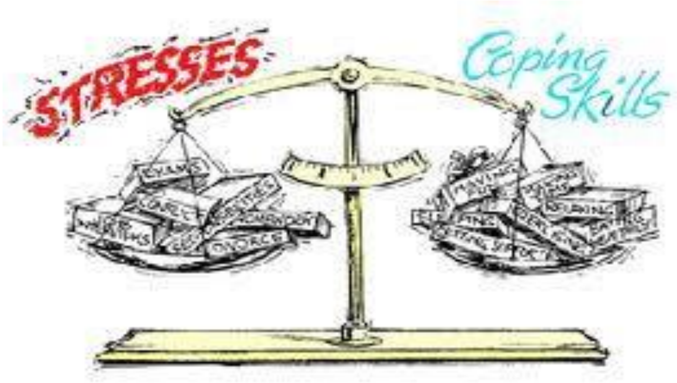

Aim:
The overall aim of this part is to enable the young person to explore a variety of coping strategies as alternatives to self-harm and to learn skills that help them manage difficult situations and emotions effectively. It is envisaged that by the time you reach this phase of therapy, you will have a fair idea of any relevant strategies for coping that the young person already utilizes. You might also be aware of possible new strategies that might be beneficial for them to learn. This part teaches specific coping strategies, and skills, and expands to consolidate and develop these skills further.

Agenda:
1: Bridge from the last session
2: Homework review
3: Any issues raised by the young person
4: Main session topic
5: Homework plan
6: Feedback

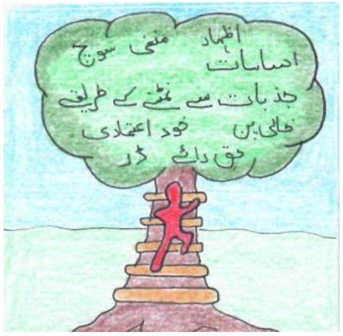

Main session topic:
Use the **"Worksheet: The coping tree"**. Ask the young person to look at the tree and explain that you will take them through the diagram. Tell them that this is a way of deciding which coping strategy to use when they are faced with a 'surge of emotion'. Explain that sometimes it is best to challenge negative thoughts immediately in order to alleviate feelings. However, on other occasions, it might be difficult to identify such thoughts or quicker and easier to accept a thought/emotion and let it go for the time being, with a view to dealing with the problem later, when they are feeling less emotionally aroused.

Explain the young person that they can use the coping tree whenever they notice a strong feeling on their feelings scale (5 or more). Starting from the bottom, they should 'go up' the roots of the tree to make a decision about the best course of action — which coping strategy to use. At this point, the young person needs to take a step back from the situation and analyze what is going on — what is happening in their environment and what is happening in their head and body, as measured through their thoughts and emotions. You might want to help them pick a recent emotionally loaded event and try this out.

After they have figured out what is going on, the young person can move up the tree trunk, where they have two primary options. They can move to the left and try one of the change-based strategies if there are obvious negative interpretations or NATs (identified through completion of a help triangle).

Sara's Coping Tree

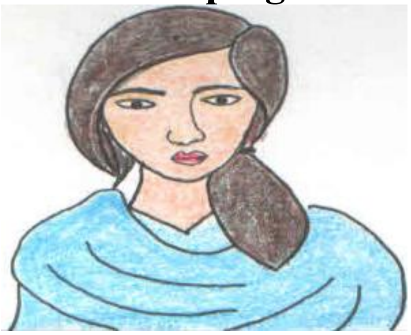

Sara described an intense feeling of emptiness and loneliness, when her friend did not call around to help her with her homework. She rated this 6 on the feelings scale. Using the coping tree as a guide, she was able to separate herself from the situation that had triggered her emotion.

She reflected on the way she handled the situation in terms of her immediate thoughts and behaviors. For instance, one automatic thought that came into her head was: 'Everyone always lets me down; no one is there for me.' Her physical response was to cry and she described how her body felt numb. To alleviate her feeling of 'emptiness', she cut the top of her leg with a razor.

Reviewing the situation, Sara was able to come up with several thoughts to challenge her original negative, dysfunctional, ones. For example, she was able to think of times when her family and friends *had* come to her aid and helped her with various tasks. She was also able to look at the situation from her friend's perspective and remembered that her friend had been very busy and had simply forgotten to call. There was no reason to think that her friend did not like her.

Another coping mechanism might have been for Sara to let go of her negative

Alternatively, they might just feel the strong emotion, in which case they should go to the right and try one of the acceptance-based strategies. Explain that they can alternate between these two options, but they should always give the one they have chosen a full go before moving across to the other side.

Deciding which side of the tree to choose is often based on the level of distress the young person is feeling and how skillful they are at using cognitive restructuring techniques. It is often very difficult to challenge thoughts or use change strategies when you are feeling extremely anxious, angry or depressed, so it can be useful to use the mindfulness/ acceptance strategies to reduce the level of distress before trying them out.

Worksheet: The Coping Tree
Note: if Participant facing it difficult then 2-3 example would be enough

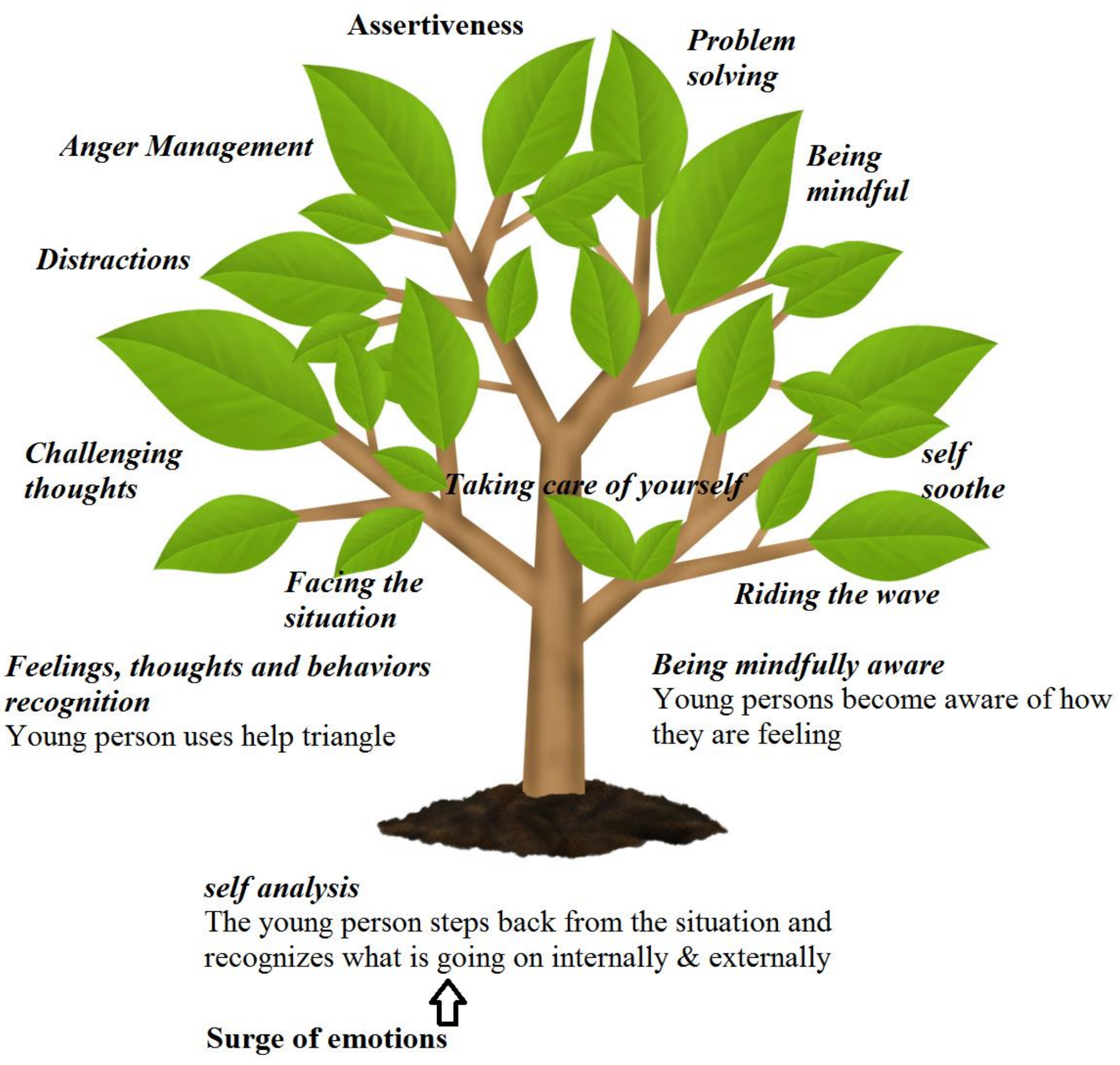

Coping Tree Examples:
Distractions: (backward counting/ reading book, riding cycle)
Assertiveness: (role modeling, learn to say no)
Anger Management: (do some exercise,take a time out)

Problem Solving: (Pros and Cons)
Challenging thoughts: (evidence for and evidence against)
Being mindful: (focus on here and now, your senses)
Self soothe: (warm bath, watching favorite TV show,listening to your favoriate music or taking hot cup of tea)
Riding the wave: (As the wave comes in you breathe in and as it goes out you blow out the anxiety and sometimes say the word ‘relax’ in your mind. Pay particular attention to your breathing and imagine the wave getting smaller and smaller).

Problem solving:
Feeling depressed, low in mood, anxious or lack ofconfidence can disable our ability to solve problems effectively. Furthermore, some young people have *never* acquired the skill; instead, they have learned to manage their distress through dysfunctional behaviour, such as self-harm. Alternatively, any attempts they have made to solve problem might not have been reinforced, or might even have been punished by their environment. For instance, a teenage girl who is struggling at school might suggest moving to a less academically orientated establishment. Instead of thinking this through and supporting her proposal, her parents might get angry and forbid the move because they cannot understand why their daughter would want to leave a 'good school'. Although they might not intend to be punitive, their behaviour does not show any understanding of or support for their daughter's attempt to try to solve a difficult problem.

The main aim of this section is to enable the young person, to generate alternative strategies to self-harm. Tell them that hassles and problems are aspects of everyday life for everyone. Parents, friends, boy/girlfriends, school, work - almost anything - can create problems in our lives at one time or another. Luckily, we are usually quite good at coping with many of these problems and are able to address them quickly and successfully.
However, other problems can be more difficult to resolve. This might be because:

- They happen fairly often.
- They have been around for some time.
- They feel totally overwhelming (this can happen with either one big problem or lots of smaller problems).
- They seem to affect everything you do.
- They occur in a context of high emotional arousal.

Problem-solving involves breaking down a worry or an issue into a specific problem,

analysing that problem to generate possible solutions, then attempting to fix it, if indeed it is fixable(can be solved). It is no good worrying constantly about world hunger as that is not a problem we can fix. However, it is possible to fix the problem of worrying constantly. This can be done by defining the potentially *solvable* problem (worry), generating several possible solutions, highlighting the pros and cons of each, then testing them out in turn, starting with the most plausible.

Exercise: Problem solving

Use the "worksheet: Problem solving" and go through Kiran's and Jamila's examples. Ask the young person at each step what they might do if they were in a similar situation.

Problem solving: Kiran

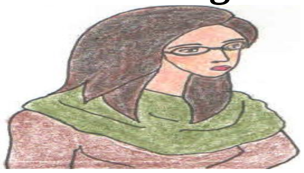

I do not know what to do. It is awful. It happened yesterday. What happened was I was on the bus with my so-called friend Saba and we were talking about my other friend Zahid. I only said he was nice. But she got totally the wrong. I arrived at school this morning and everyone is talking behind my back. Zahid's girlfriend is giving me evil looks and I am scared she is gonna hit me. Everyone is laughing at me. I cannot ever go back to school.

Problem solving: Jamila

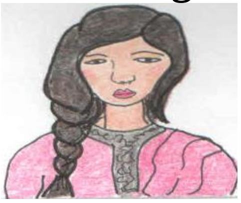

This always happens. I am sick of his stupid rules, he makes my mum do what he thinks and he does not even live at home. Why cannot he just leave me alone and let me have a life. This time it is about this school festival that everyone is going to. It is so stupid. I am not allowed to go because apparently it finishes too late for a school evening. What does he think he is? He has never cared about me before.

Step 1: Identify the problem

How is Kiran going to deal with her friend, Zahid and other people at school?

Step 2: List every possible solution

1: Never go to school again.
2: Send Saba a nasty email.
3: Arrange a fight with Zahid's girlfriend.
4: Ignore the people laughing.
5: Go home, cry and take some tablets.
6: Confront Saba and sort it out.
7: Talk to Zahid and his girlfriend about what really happened.
8: Ask another friend to talk to Zahid.
9: Tell a teacher.

Step 3: Assess each possible solution

For example, for possible solution 1: Never go to school again:
Pros: I don't need to feel embarrassed; I don't like school anyway.
Cons: My dad would kill me; I won't get admission in college; they will think they have won and I will feel weak; I will lose all my friends.

Step 4: Choose the best solution or combination of solutions

Solutions to try following after assessing all possible solutions in Step 3 (in order, with best option first)

7: Talk to Zahid and his girlfriend about what really happened (although it won't be easy, there are more pros than cons for this option).
4: Ignore people laughing because I don't want them to feel that they have won.

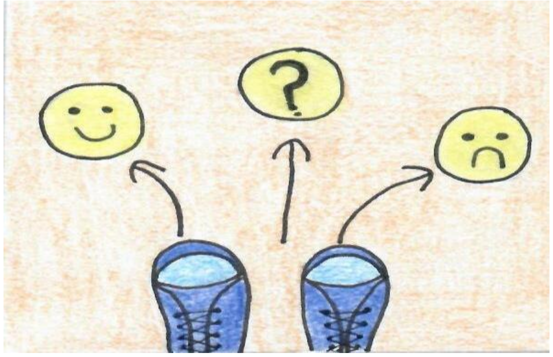

Step 5: Plan how to carry out the best solution

- Jot down what I want to say so it's clear in my head.
- Plan a good time to catch Zahid with his girlfriend on their own (after

English class).

- Let them know that I want to *talk* to them, not fight.
- Explain the situation that I only like him as a friend and I'm not trying to steal him from her! Saba got it all wrong.
- Make sure I've got my friend to talk to afterwards.

Step 6: Review how it's going

- What obstacles might get in the way?
- How might you deal with them?
- What is your plan B?

This could work and the problem will be solved, which would be great. But if it fails, remember kiran has lots of other options to try.

Finding a solution that works: Kiran

'I tried really hard to find them on their own, as there were always loads of people with them and I just couldn't do it in front of everyone else. So I wrote them a note instead and it's all OK again now.'

Now ask the young person to come up with their own example and go through the six steps with them:

1. Identify the problem.
2. List every possible solution. (Be as open-minded and creative as possible with potential solutions. It doesn't matter how extreme they are. The idea is to get the young person thinking.)
3. Discuss the pros and cons of each possible solution.
4. Choose the best solution or combination of solutions. (This might be the one with the most pros and the fewest cons, or it could simply be the one that the young person is willing to try).
5. Plan how to carry out the best solution.
6. Review how it's going:
    - What obstacles might get in the way?
    - How might you deal with them?
    - What is your plan B?

Remember, if the first solution doesn't work, you can always go back to Step 4 and try a different one.

Homework:

- Identify a problem and go through the six steps.

- Give handout on coping tree

Worksheet: Problem solving

Step 1: Identify what is the problem?
Parents not allowing me to study further

Step 2: List every possible solution

-try to convince them to study till they get some good proposal
-talk with aunt or uncle to support your decision and discuss this with parents
-can try permission for tuition or can do self study and appear in private exam
-enroll herself in some vocational training center to get command in some skill

Step3: Assess each solution

Possibility	Pros	Cons
Continue study till they get some good proposal	Parents continue their search and till then I get chance to appear in exam	My parents would put more restrictions on me

Step 4: Choose the best solution.

-discuss with aunt to convince parents for private exam

Step 5: Plan how to carry out the best solution.

-collect information for private exam, expense and procedure from teacher and friends
-jot down all points in mind before talking to aunt (like go for exam with aunt or brother,wants to continue study till she get married, tried to bear expense by stiching cloths)
-talk with aunt secretly and inform her about whole procedure of private exam

Step 6: Review how it is going.

-aunt successful is convicing parents for my private exam.

Hand out for the young person: Session 7
The Coping Tree: Which strategy should I use?

The coping tree is a way of deciding which coping strategy you should use when you are faced with a 'surge of emotion'. Sometimes it might be most useful to challenge negative thoughts to reduce intense feelings. At other times, it might be difficult to identify the thoughts or it might be quicker and easier to accept a thought/emotion and let it go, with a view to dealing with the problem when you are less emotionally aroused.

The bottom of the tree is the place to start. This is when you first notice a strong feeling (a rating of 5 or more on your feelings scale). When you have noticed the feeling, 'go up' the roots of the tree and make a decision as to the best course of action — which coping strategy to use. At this point, you should try to take a step back from the situation and analyse what is going on. Think about what is happening around you and what is happening in your mind and body. Focus on your thoughts and emotions.

After figuring out what is going on, move up the tree trunk. Now you have two alternatives. You could try one of the change-based strategies if you know you have put a negative spin on the situation or you have noticed any NATs; use a help triangle. Alternatively, you might just feel the strong emotion, and have no obvious thoughts, in which case you might want to move to the right-hand side of the tree and try an acceptance-based strategy. You can alternate between these two options and your therapist will explain more about them and run through some examples with you in future sessions. If you do alternate, you must try to give the strategy you picked first a good go, before moving to the other side.

Remember, it is often very difficult to challenge thoughts or use change strategies when you are feeling extremely anxious, angry or depressed. So it can be useful to use one of the mindfulness/acceptance strategies first, to reduce the levels of distress. Then, when you are feeling calmer, employ one of the change strategies.

Session 8
Assertiveness
Continuing Journey

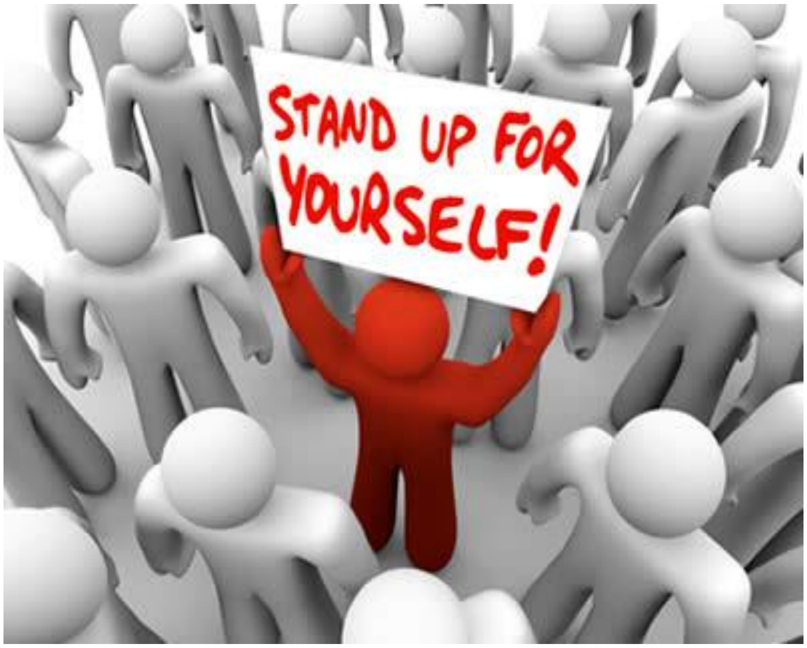

Aim:
The aim of this section is to enable the young person to assert their needs, desires, wishes and feelings effectively.

Agenda:
1: Bridge from the last session
2: Homework review
3: Any issues raised by the young person
4: Main session topic
5: Homework plan
6: Feedback

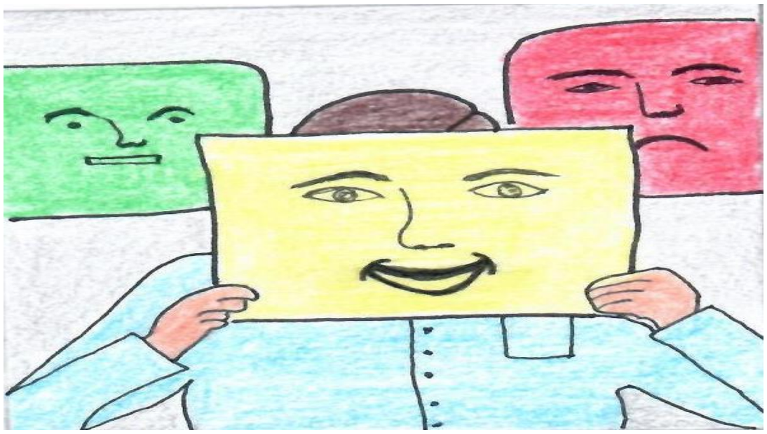

Main session topic:
Assertiveness is an essential skill that is required for healthy and productive relationships. Problems are encountered when issues arise between people and when the immediate behaviours used are either passive or aggressive in nature. Possessing the ability to behave assertively can help avoid any feelings of pent-up frustration, which can often result when a person ends up doing things they do not want to do, leading to feelings of regret, self-disgust and, ultimately, self-harm.

The first key skills in being assertive are saying what you really feel and negotiating what you want. Ask the young person: 'Do you sometimes find yourself going along with something when you don't really want to, and then feeling angry or upset with people afterwards?' If they relate to this feeling, continue with the discussion. (Bear in mind that some young people might struggle with the opposite problem — getting angry and aggressive when pressure is placed on them. However, even if this is the case, further discussion of assertiveness should be useful).

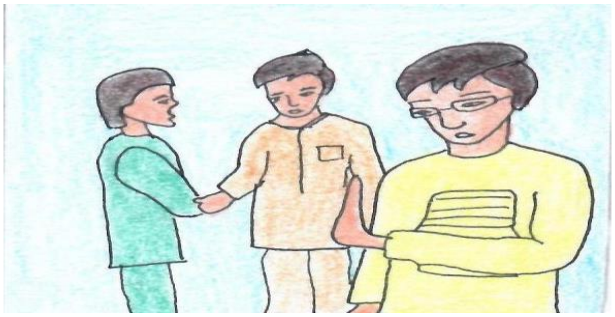

Tell the young person that it's not always easy to say 'no' to someone, especially a friend, and explain that this can be particularly difficult when we want to fit in and don't want to stand out as being different. Many people worry that they won't be liked if they say what they really feel. The idea of not being liked is pretty horrible, so there is pressure to do things we don't really want to do, including: revealing intimate details about our lives; taking drugs; skipping school; or going to a party.
However, *always* going along with others and trying to please them usually leads you to feel worse about yourself and reduces the respect other people have for you. Standing up for your rights or having a different opinion means that it is sometimes essential to say `no'. There are various ways to do this. For example, it can be done aggressively, by shouting at or threatening the other person, but this is usually ineffective, because it just makes the other person angry and can lead to arguments. Alternatively, saying 'no' can be done in an assertive way: you stand up for yourself in a calm manner, while continuing to respect the other person's feelings and wishes. This usually works very well, but a lot of young people (and many adults!) find it very hard to do.

Exercise: Asserting yourself
Use **"worksheet: Assertiveness"**. Look together at Sara's options for dealing with her difficult situation and ask the young person if they can remember times when they have felt under similar pressure. Then discuss the possible consequences of submitting to peer pressure.

Sara's situation:
'Although it was a huge effort, I did go to my friend's party but when we left my friends decided to go for shopping. I didn't want to, because I felt too tired and miserable, and I just wanted to go home'.

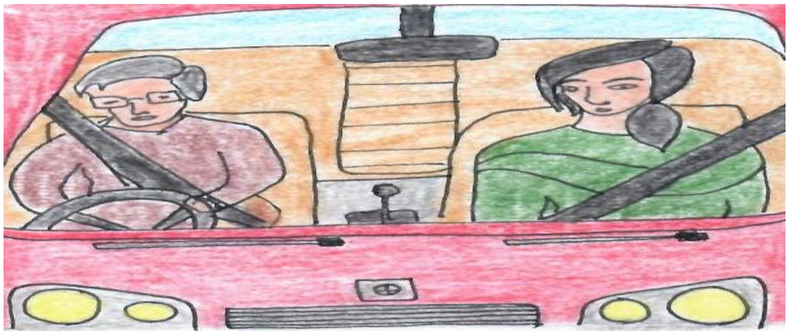

Sara’s options:
The 'pleasing others' response: 'I ended up going and hated it! Then I got into a lot of trouble as I had to get my step-dad to pick me up. I felt so horrible that I went home and cut my arm really badly. I wish I'd stuck to what I wanted to do.'
The aggressive response: 'None of them appreciated that I'd come out when I didn't want to and said I was selfish because I wanted to go home. I went mad and told them all to shut up. When they left I threw a can of cock after them. Now I've lost *all* my friends!'
The assertive response: 'I really didn't want to go, so I said I was really tired and that I'd had enough. They were disappointed and tried to persuade me but I didn't budge. Eventually my friend Saman said she was tired too and she'd come with me. We had a really good chat on the way home.

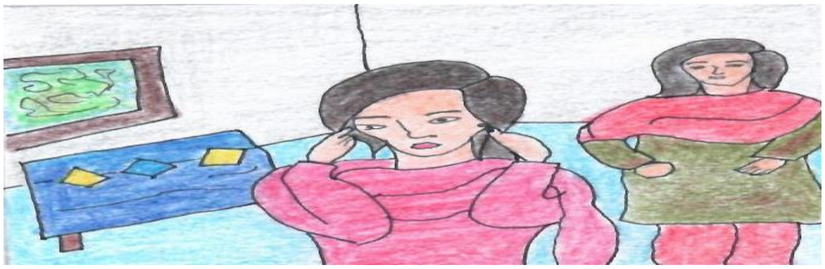

Exercise: Assertiveness role play
Now, using the blank boxes, you can explore what the young person might have done in a similar situation. They should choose a real-life situation when they wanted to say something but ended up going along with the group, even though they did not want to. (If they are unable to come up with an example, use Sara's.)

Scenario 1: The young person acts as himself/herself (non- assertive) and the therapist plays the role of friend/other.

Scenario 2: Discuss what the young person wishes they had said, or what they imagine an 'assertive person' might have said. Play around with various ideas: for

example, someone they think is cool, a strong fictional character, someone in their class, a family member, etc. Using the identified strategies, the young person should play the 'assertive person' while the therapist gives feedback on what it feels like to be face to face with such a person.

Notes: Feedback not only based on content but also on tone, gesture.(Feedback sandwich)

Scenario 3: Swap, so that the young person starts to learn how it feels to be on the other end of assertiveness. Encourage a discussion to dispel their beliefs that being assertive will result in a loss of friends and help them see that it is more likely to encourage respect.

Exercise: How do you become assertive?

Training in assertiveness involves helping the young person to develop social behaviors to cope with pressure. This is best achieved by planning, rehearsing and practicing refusal behaviors.

Using “Worksheet: Assertiveness”, go through the following three steps with the young person.

Step 1: Preparation

- ***What I want to say:*** Describe the situation or the problem that is important to you, rather than focusing on the other person or their actions. Try to be as specific as possible: for example, 'I am thinking that you all want to go to a movie.'(mimimum effective response)
- ***My feelings:*** Say how you feel about the situation or problem: for example, 'I am sorry/sad I won't be able to go with you to the movie.'
- ***My needs:*** Say what you want to happen to make things different: for example, 'I need to go home to go to bed.'
- ***The outcome:*** Being assertive and behaving in a certain way will improve the situation for you *and* for the other person: for example, `If you all go to the movie and I go home, we will all be happier.'

Step 2: Strategies

Use **“Worksheet: BEST skill”** - an acronym to help the young person to remember the basics when they need to be assertive in real-life situations:

- Be clear - state what the situation is.
- Express your feelings - use 'I . . .' statements, not 'You make me feel . . .' statements.

- Say what you want calmly and simply.
- Try to negotiate if it is not working, or if you meet with resistance.(communication training)

Once the young person is familiar with this basic guide, they can explore more skills. Go through them together and ask the young person to think about situations when such strategies might be useful (Worksheet: Assertiveness).

- ***Broken record:*** When you want to stick to your guns, try imagining you are an old style vinyl record that has got stuck, or a CD that is scratched, saying `no' or expressing your opinion over and over again. No matter what the other person says or does, just keep repeating the same point.

- ***Script it:*** Write down what you want to say beforehand and rehearse it.

- ***Ignore:*** If you are feeling pressurised in any way, try ignoring what the other person is saying or doing. This can be really hard to do, but it sends out a powerful message!

- ***Turn the tables:*** Turn the problem over to the other person. Ask them to think of something you can do together, but don't agree to do anything that is unappealing: for example, 'I can't say "yes" to that, even though you want me to. What can we do about it?'

- ***Act cool:*** Concentrate on acting confident, even if you don't feel it. Imagine how a friend or someone you admire might behave in the situation and try to act in the same way.

- ***Give to get:*** Sometimes you have to 'give to get'. Without forgetting about what you want, try to find a way to meet the other person half-way. See if there is something else you can offer while maintaining your 'no'. Maybe reduce your demand to something that can be fulfilled.

What happened when Sara used an assertive strategy?

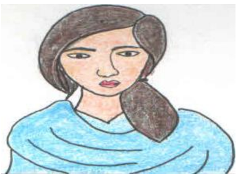

'My friend wanted me to not attend English class with her as she hadn't done her homework. I knew she was really worried about it and I didn't want to let her down, but at the same time English is my favourite lesson! It's the only one I really enjoy at the moment, as we are writing poetry, and really didn't want to miss it. I was also worried that we will get in trouble with the school if we will miss this class. She said I was selfish if I went to the lesson, as then she would have to go too. I decided to try being a "broken record". Told her she was worried about her homework but I wasn't going to miss the lesson with her. She tried really hard to convince me but I just kept saying, "No. I like English. I'm not missing this class." Felt stronger every time said it! After a while she accepted it and we talked about what excuses she could give for not doing her homework.'

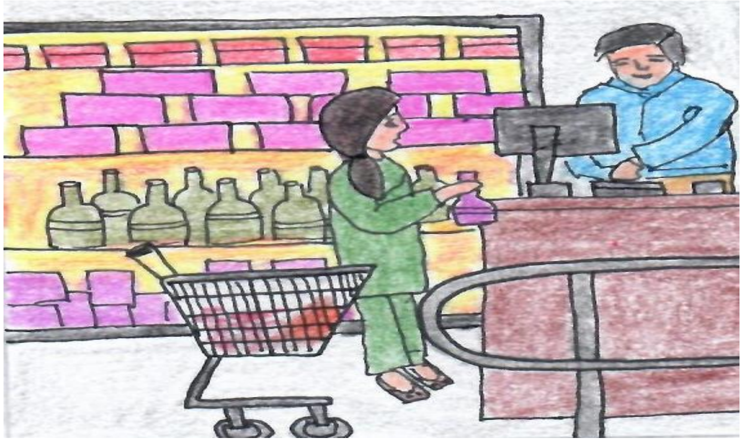

Step 3: Practice

Being assertive is not always easy, and the best way to build confidence is through practice. Ask the young person to think of situations in their day-to-day life where they could practice being assertive. (Remind them that it might be easier to start with small steps). If nothing comes to mind, you could suggest the following examples (see Worksheet: Assertiveness).

- Go to a shop and ask where something is (e.g. in a supermarket, ask for the shampoo).

- While talking with someone, change the subject to something you want to talk about.
- Ask for no sauce/a different topping in a fast-food restaurant or stall.
- Ask for the bill when you're out for a meal with a friend.
- Ask the shopkeeper to show you some more dresses' options.
- Call a tuition teacher and ask if you can attend a class and pay separately
- Invite a friend out to the shopping.
- Go into a shop or petrol pump and ask for change for the bus.
- Go into a neighbor home and ask for some water.
- Ask a friend to do you a favour.
- Send back a meal in a restaurant because it is too cold/too small for the price.
- Give someone a compliment/accept a compliment by saying 'Thank you'.

The next skill you will be discussing with the young person is a continuation of the skills that enable them to respect their own needs while also getting along with other people. They will have a clear idea about the link between their thoughts and emotions and in particular about any negative thoughts they have about themselves.

Session 09

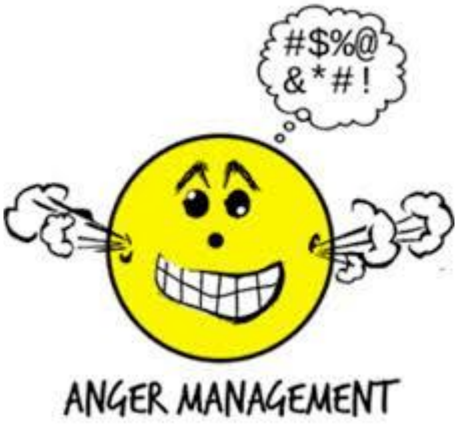

Anger management:

If it has not already come up as an issue, ask the young person if they have ever got into trouble because of their anger, or if it has wrecked a specific situation for them. If they say that this is not a problem for them, you can move on to conclude your therapy sessions.

However, if they admit that they have had trouble with their anger, stick with this module. Explain that anger is not a problem per se (look back at the exercise 'Feelings are our friends'). A problem arises only if anger impacts negatively on relationships or impairs the young person's functioning in another way. For example, see Ahmed's anger scale (Figure: Ahmed’s anger scale). His anger can get out of control at times and he has been suspended from school for hitting another pupil in front of a teacher.

After looking at Ahmed's example, tell the young person that the first step in anger management is to learn how to become aware of the stages of anger as it develops. Then it will be possible to escape from the situation by using adaptive interpersonal skills. (e.g. assertiveness; or mindfulness, then distraction) *before* the anger gets out of control.

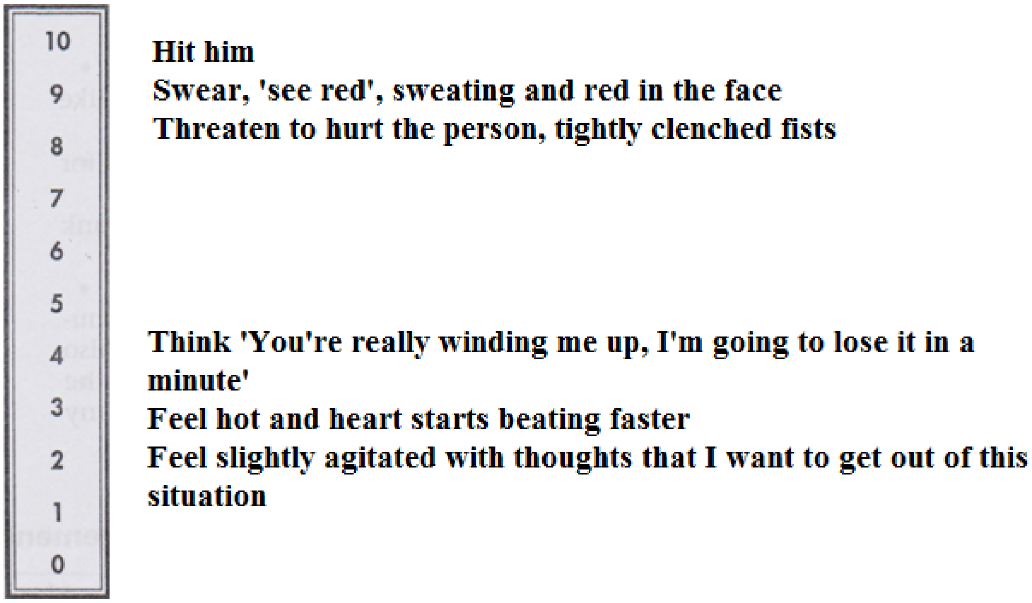

Figure: Ahmed's Anger Scale

Exercise: My Anger Scale

Use **"Worksheet: My anger scale"** and ask the young person to rate how quickly they move from a 1 to a 10 when they are angry. Write the stages of anger next to the relevant positions on the scale to identify what happens as the anger escalates. When you do this exercise with the young person, try to identify their physical sensations at the various stages, as well as their thoughts and any actions or predicted actions. The more sensations, feelings, thoughts and actions they can identify at each stage/ (body signals), the easier it will be to stop the process early.

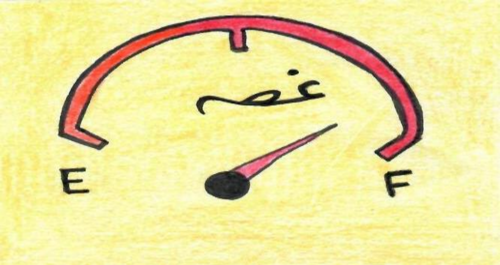

Exercise from Living life to the full (anger)
Exercise: Anger Management Techniques

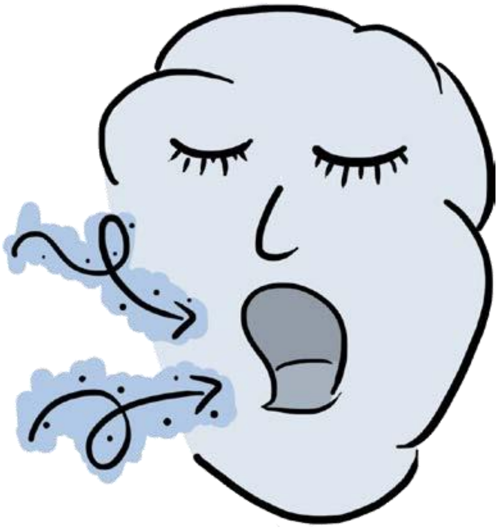

Take 20 Deep Breaths

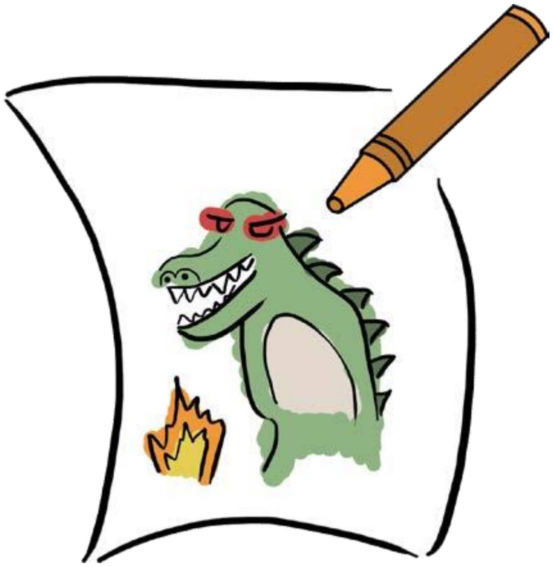

Draw Your Anger

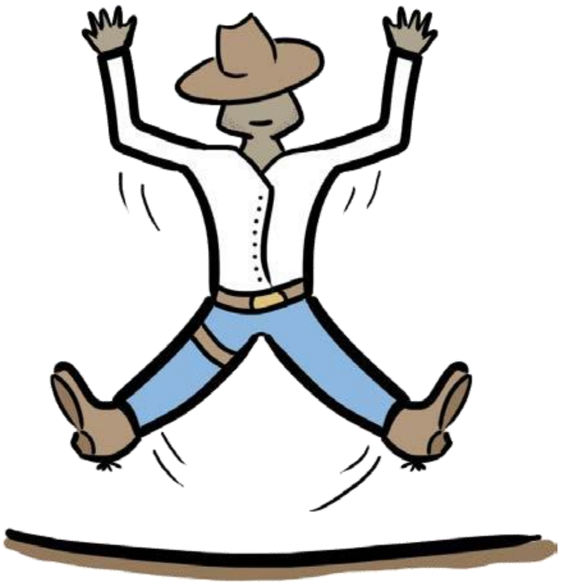

Do 50 Jumping Jacks

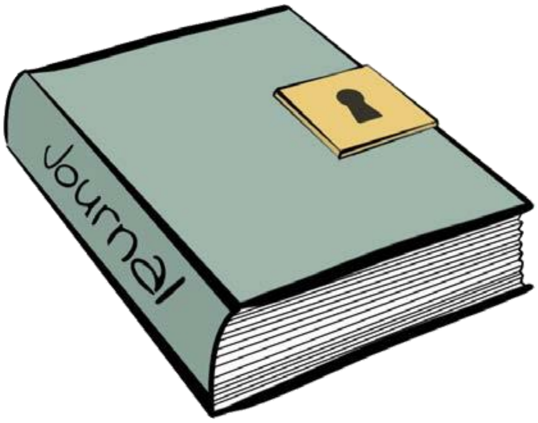

Write About Your Anger

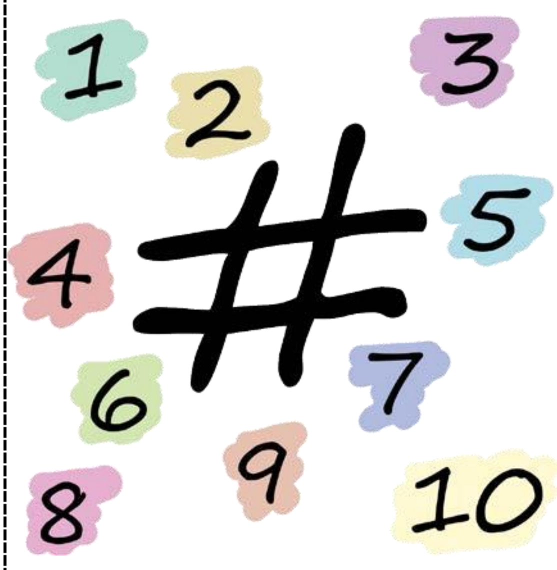

Count to 100

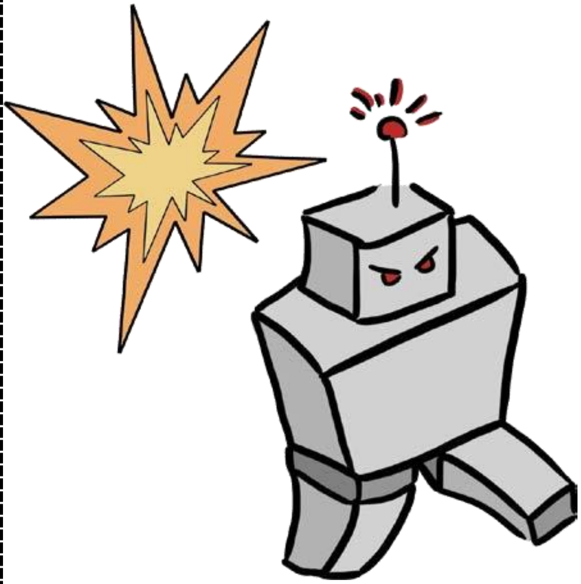

Walk Away

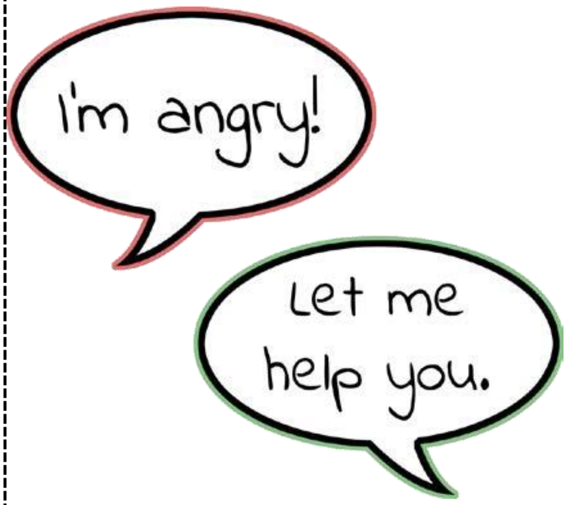

Talk to Someone about Your Problem

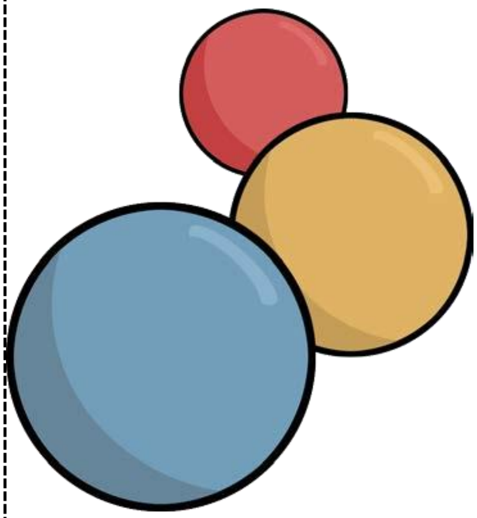

Squeeze a Ball

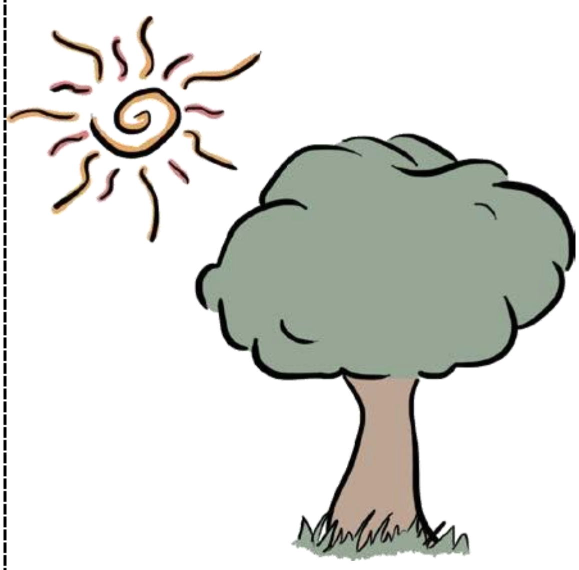

Play Outside

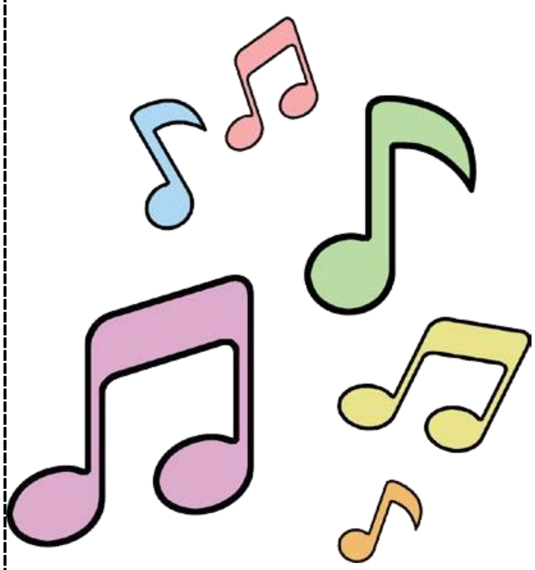

Listen to Music

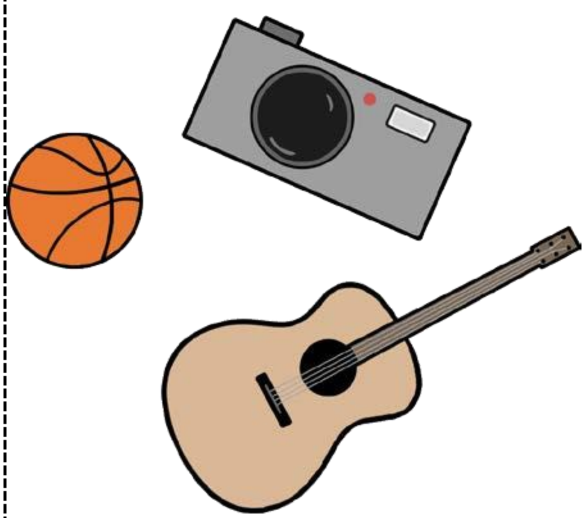

Practice a Hobby

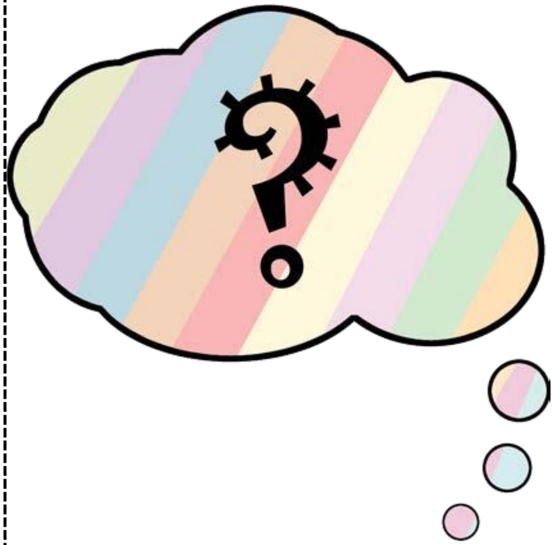

Your Own Idea:

Exercise: Imagination task
Ask the young person to think about the last time they got extremely angry and completely 'lost it'. Then tell them to imagine reaching 2 or 3 on the anger scale. State the signs that correlate with this stage which the young person identified in the previous exercise. Then get them to imagine employing a coping strategy (e.g. walking away or being assertive, as appropriate). Practice imagining the triggers for the anger (e.g. someone winding them up) but remaining calm. This exercise needs to be practiced every day, so it would be useful to schedule it as a homework task for this session.

Continuing journey
In order for the young person to continue on their journey and 'stay well', their life has to have balance. They will need to continue to make and maintain healthy relationships, which will include using their assertiveness skills, anger management skills and other cognitive strategies. Moreover, they will need to identify a network of supportive, trustworthy friends and family (something they should have done on their relationships map, which they could redo now, if they think it would be useful). It is also important for them to identify any people who are more likely to encourage self-destructive patterns of behavior.

Another important element in keeping well and moving on is to achieve your life goals and maintain your values, as far as possible. These goals and values might be study-related or hobby-related, or they could include other things that help the young person to establish a sense of purpose and meaning in their life.

Overall, young people who have a history of self-harm need to learn to 'take good care of themselves'. This involves maintaining a balance between achievement and fun, accepting support from others and doing things for themselves, and trying to follow a generally healthy lifestyle (through exercise, a good, well-balanced, healthy diet, a regular sleep pattern, etc.). However, it is important to recognize that exceptions to these general rules are acceptable, as long as they do not lead back into self-harm or other dysfunctional behaviors.

Exercise: Tool kit and tool box

Use **“worksheet: Tool kit and tool box”** and explain that this is a 'crisis' first-aid kit: it should be used when the young person is in distress or experiencing intense and difficult emotions.

Now take the folder of worksheets that the young person has worked through over the past few weeks and your reference book and help them identify which particular strategies they find most useful at specific times and what they would need to get back on track in a time of crisis.

Discuss how ready or able they feel to try out the identified strategies and add the best ones to the spaces in the first-aid kit.

Sara’s Tool kit:

What Sara will do to stay well?

- Make sure I keep up regular scheduling of activities that give me a sense of pleasure and achievement.
- Keep my list of thought-challenges in my phone and the poster I have made on my wall.
- Keep a copy of the coping tree in my phone and on a small laminated card in my purse, so I can easily find the best skill to use when I can't think straight.
- Remember to balance my time, so that I don't end up spending too much time with one friend or just my family. Remember to schedule in 'me time'/'taking care of myself'.
- Continue to practice my assertiveness skills, rather than 'flying off the han
- dle'. The more I practice, the easier it will be to manage my anger.

Session 10 Family Session

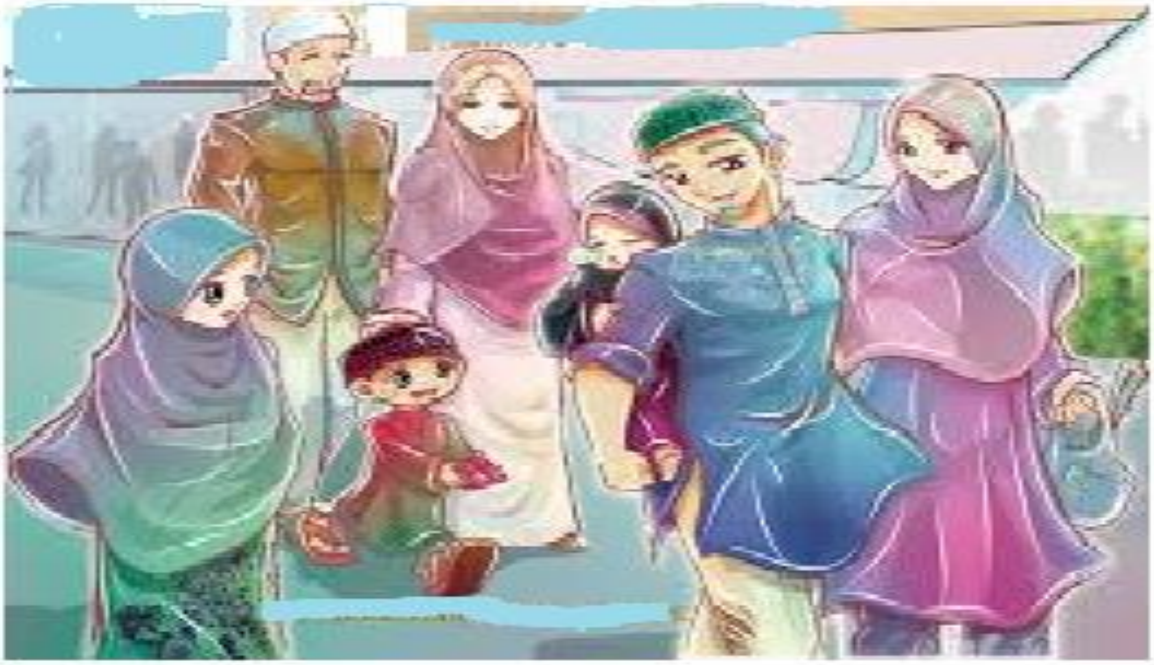

You may have given permission to look at this book by the person you Know who has harmed himself or herself. Have just stumbled across this book and did not know that your friend or relative has harmed themselves. Either way it is likely that the fact that someone close to you has harmed herself or himself has a deaf effect on you .It will probably have come as a shock to find this out.

You may feel up set angry or confused about what has happened. You may think that others may blame you as having somehow contributed towards a situation that your relative or friend found too difficult to bear and tried to seriously harm him or herself. You may have even thought that you are responsible and if only you had done or said something differently this would not happened.

Before you read on, we want you to know that you are not responsible for another person trying to harm themselves. When someone tries to harm themselves, they are unlikely to be thinking in a logical way and are likely to be viewing their problems as being overwhelming. Their resources to cope with their problems will have been greatly diminished and harming themselves may have seemed to be their only way out of an unbearable situation. Not only should you not blame yourself you should also try not to be angry with them for having tried to harm themselves.

Most people once they have recovered from harming themselves realize that their friends and relatives do want to help them and may have noticed that their friends and relatives do want to help them and may have noticed that something was wrong and that they were behaving out of character. Your relative or friend

may have seemed to be depressed or overly sensitive or emotional to you in the recent past. It is likely that they have been struggling with their problems and have not been able to share the depth of their distress with anyone.

We know that when a person in such a state of distress is suicidal, they often think that they cannot ask for help, or accept help if is offered. Often an individual will feel ashamed of being in such adept start of distress or in a situation that they cannot readily resolve. They will often view their only way out of the situation as escape by self-harm. It is as if they have already closed off alternative options and ways to solve problems, and are only focusing on escaper away out of intolerable distress.

We know that you may be asking yourself why they or why they could not ask for help as you would have been more than willing to help, but in such deep distress they are unlikely to have viewed this as a realistic option.

Mark Williams, in his book the Cry of Pain, has described this and a catastrophic failure of empathy. He believes that suicidal despair switches off the ability to understand how others will react and feel. Although to you, the individual who has harmed himself or herself may appear to have disregarded the feelings of those close to him or her, it is likely that your friend or relative had little control over this and was unable to think through the potential consequence of self-harm for those to whom they are close.

What can you do now?

There may be no single right thing to say or right way to behave in this situation, But there something you can do to help both yourself and the person who has self-harmed.

Your own feelings

The first thing you should do is to be aware of your own feelings about your relative or friend having harmed him or her. You may feel guilty, thinking that you have in some way contributed to be person close to you having harmed them. You may feel concerned and anxious that this will happen again and that you do not want to say or do something that may make the situation worse.

You may feel shut out by the person who has self-harmed. It might be helpful to talk to someone you trust about your own feelings so that you can have some support for yourself in this situation. You may find that you are not alone in

feeling the way you do as someone harming him or she affects many people. Often there are several people who have felt that they should be doing more to help someone who has been talking of harming himself or herself or behaving in a manner that suggests that they are distressed or troubled.

What if you have been implicated or blamed for the person self-harming?

Clearly this would be a painful situation and you are likely to feel very distressed by this. You may feel angry. Try not get drawn into the situation further and not to over-react. Trying to establish who is to blame, or who is being blamed is a pointless exercise. Try to move beyond this point. Again find someone who is open-minded to talk to before you talk to the person who has self-harmed. Most importantly, open up a dialogue with the person who has self-harmed. They are likely to have misunderstood your motives, feelings and behavior. If you take a non-critical stance about why they have self-harmed and listen to what they have to say, then there is a reasonable chance that you will be able to clarify any misunderstandings that have arisen. What matters now is that they see you as someone who cares for them and who can help.

Non-critical listening

Many people who self-harm are afraid that others would have criticized them for not being able to resolve their problem or for having restarted to harming

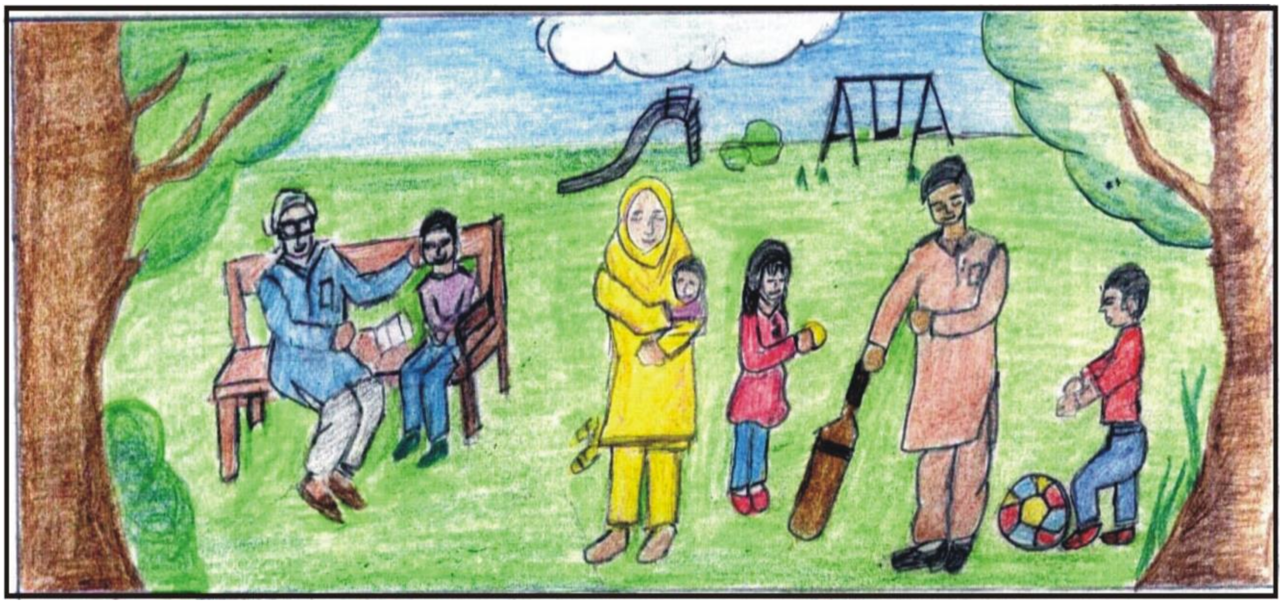

themselves. It is important to listen to what the person has to say about their feelings and problems without being judgmental. As we have stated before, most people who try to harm themselves seriously have not been stated before, most people who try to harm themselves seriously have not been able to think clearly about the situation they are finding difficult and often feel too ashamed to talk to others about how they feel and think. By taking the person seriously and listening without criticizing, you help a great deal to restore an individual's sense of self-worth and confidence in others.

Increasing self–worth

After listening, you can tell them that you value them and that they do matter to you and to others. Ask how you can help further but be prepared just to be there if you are needed and most importantly, keep communicating.

Getting involved with life again

We know that this may be a very emotionally draining time for everyone concerned. Talking and listening will help but it is also helpful to begin to do things together again. The individual who has harmed themselves may have been less active, more depressed and more withdraw before they harmed themselves-as if they were disengaging from life. It will be helpful to involve them in activity again. Simple things such as going for a walk together, shopping, gardening or going out for coffee and cake can help to bring them in touch again with life. 130 **CMAP**

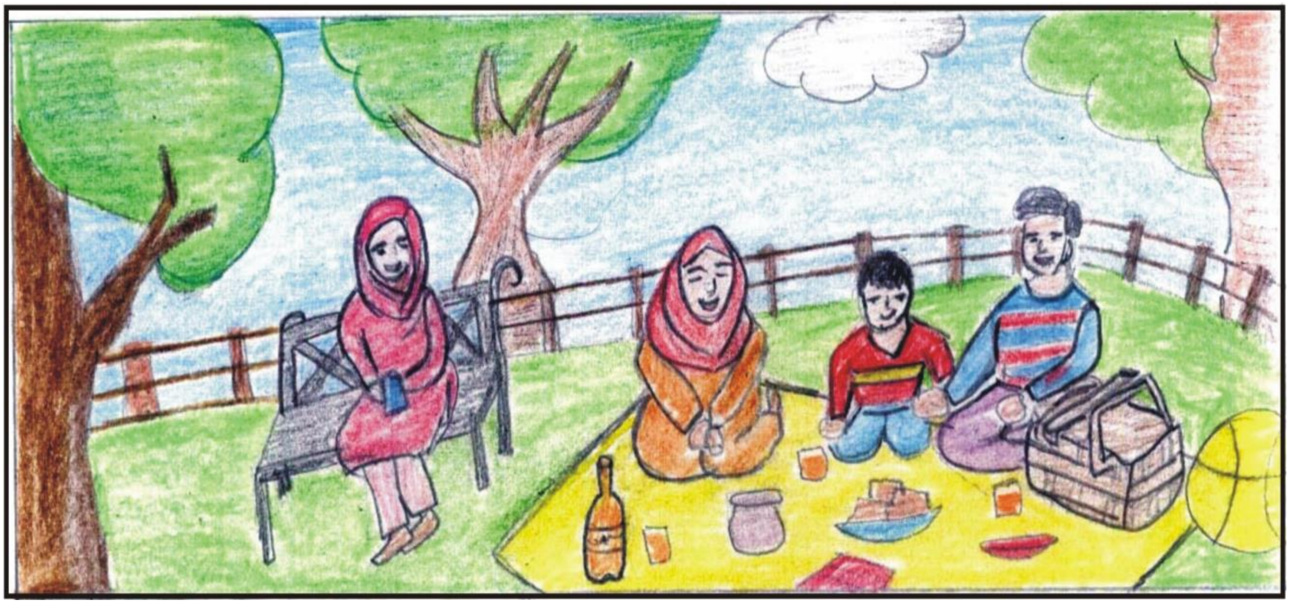

Professional help

Lastly, after the crisis has passed and everything may appear back to normal, do not assume that it way stay the same. If someone has gone as far as to harm him or herself seriously, the problems are unlikely to disappear completely in a short time. You can still keep communication between you open, talking and listening to them and helping them to get involved in things again, however, outside help may also be needed and you might want to encourage the individual to seek help through their doctor. In this way they will also have a professional person to help them.

The certificate:

Conclude the program by awarding certificate to the young person. You should summarize key points you have observed from the sessions spent with the young person in the spaces provided. Think carefully about useful points for feedback that are person-specific, honest and useful for the young person to take away.

This is to certify that

____

Has successfully completed the YCMAP program

Particular achievement are;

____

____

Signed:---------------------

Good Luck
