# Supplementary material for: Effectiveness of YCMAP (youth culturally adapted manual assisted problem solving) intervention in adolescents after self-harm in Pakistan: multicentre, randomised controlled trial
Source: BMJ. 2025 Sep 12;390:e083272. doi: 10.1136/bmj-2024-083272 (PMC12426884; doi:10.1136/bmj-2024-083272)

Supplementary Table 1: Proportion of missing data across different variables

	Complete	Missing	P*
Age			0.035
12-15	223 (33)	0 (0)	
16-18	451 (67)	10 (100)	
**Marital status**			0.066
Single	652 (97)	8 (80)	
Married	20 (3)	2 (2)	
Separated /Divorced	2 (0)	-	
Number of earning members in family			0.12
1	400 (59)	3 (30)	
2	182 (27)	4 (40)	
3 or more	92 (14)	3 (30)	
Total income in Rupees			0.78
<15,000	152 (23)	1 (10)	
15,000- <30,000	296 (44)	5 (50)	
≥30,000	224 (33)	4 (40)	
**Gender**			0.081
Male	317 (47%)	2 (20%)	
Female	357 (53%)	8 (80%)	
**Status of home**			0.34
Own	434 (64%)	5 (50%)	
Rent	240 (36%)	5 (50%)	
**Education**			0.60
No formal education	89 (13%)	3 (30%)	
Less than primary (5yrs)	189 (28%)	2 (20%)	
Middle / Secondary (8yrs)	180 (27%)	2 (20%)	
Matric (10yrs)	159 (24%)	2 (20%)	
Intermediate (12yrs)	48 (7%)	1 (10%)	
Other (diploma)	9 (1%)	0 (0%)	
**Employment**			0.60
No	582 (86%)	9 (90%)	
Yes	92 (14%)	1 (10%)	
**Intent to die (Suicide Attempt Self Injury Interview SASII)**			0.049
Obviously no intent/Minimal	80 (12%)	4 (40%)	
Definite intent but very ambivalent	185 (27%)	2 (20%)	
Serious intent/Extreme intent	409 (61%)	4 (40%)	
**Communicated about self-harm.**			0.040
No	538 (80%)	5 (50%)	
Indirect communication	20 (3%)	1 (10%)	
Direct communication	116 (17%)	4 (40%)	

*p-value from Fisher's exact test

Supplementary Table 2: Analyses of primary outcome after reweighting the data using stabilised inverse probability of missingness weights

Treatment Group	No Repetition	Repetition	Odds Ratio (95% CI)	P-value
E-TAU (Reference)	93%	7%	Ref	—
YCMAP Plus E-TAU	99%	1%	0.09 (0.02 to 0.35)	<0.001

Supplementary Table 3: Analysis of primary outcome after assuming that all those who were censored by 12 months had a self-harm event

Treatment Group	No Repetition	Repetition	Odds Ratio (95% CI)	P-value
E-TAU (Reference)[1]	325 (95%)	17 (5%)	Ref	—
YCMAP Plus E-TAU	331 (97%)	11 (3%)	0.65 (0.31 to 1.36)	0.33

Supplementary table 4: Pattern of healthcare services utilization among trial participants

Variables	Responses	Frequency		
		E-TAU	YCMAP	Total
Sought help for Physical Care	Yes	433	512	945
Sought help for Physical Care	No	929	808	1737
Sought help for Psychological Care	Yes	3	16	19
Sought help for Psychological Care	No	1359	1304	2663
Inpatient for Physical Care	Total Days	43	96	139
Inpatient for Psychological Care	Total Days	1	5	6
Out-patient clinic for Physical Care	Acquired Service for (1) time	220	111	331
Out-patient clinic for Physical Care	Acquired Service for (2) times	171	35	206
Out-patient clinic for Physical Care	Acquired Service for (3) times	60	14	74
Out-patient clinic for Physical Care	Acquired Service for (4) times	22	3	25
Out-patient clinic for Physical Care	Acquired Service for (5) times	6	0	6
Out-patient clinic for Physical Care	Acquired Service for (6) times	2	0	2
Out-patient clinic for Physical Care	Acquired Service for (7) times	2	0	2
Out-patient clinic for Physical Care	Acquired Service for (8) times	2	0	2
GP for Physical Care	Acquired Service for (1) time	122	31	153
GP for Physical Care	Acquired Service for (2) times	62	16	78
GP for Physical Care	Acquired Service for (3) times	30	9	39
GP for Physical Care	Acquired Service for (4) times	5	3	8
GP for Physical Care	Acquired Service for (5) times	2	3	5
GP for Physical Care	Acquired Service for (7) times	0	2	2
GP for Physical Care	Acquired Service for (10) times	1	0	1
Any other doctor for Physical Care	Acquired Service for (1) time	8	2	10
Any other doctor for Physical Care	Acquired Service for (2) times	1	2	3
Any other doctor for Physical Care	Acquired Service for (3) times	2	0	2
Any other doctor for Physical Care	Acquired Service for (6) times	1	0	1
Out-patient clinic for Psychological Care	Acquired Service for (1) time	4	1	5
Out-patient clinic for Psychological Care	Acquired Service for (3) times	1	0	1
GP for Psychological Care	Acquired Service for (1) time	1		1
GP for Psychological Care	Acquired Service for (2) times	0	1	1
Any other doctor for Psychological Care	Acquired Service for (1) time	12	7	19
Any other doctor for Psychological Care	Acquired Service for (2) times	1	0	1
Any other doctor for Psychological Care	Acquired Service for (3) times	1	0	1
Imams/molvi for Healthcare	Acquired Service for (1) time	95	69	164
Imams/molvi for Healthcare	Acquired Service for (2) times	1	0	1
Pirs for Healthcare	Acquired Service for (1) time	42	23	65
Pirs for Healthcare	Acquired Service for (2) times	1	0	1
Hakim/Homeopathic for Healthcare	Acquired Service for (1) time	7	4	11

Other non-medical healers for Healthcare	Acquired Service for (1) time	0	1	1

Supplementary Figure 1: Pattern of face-to-face vs remote sessions of YCMAP intervention

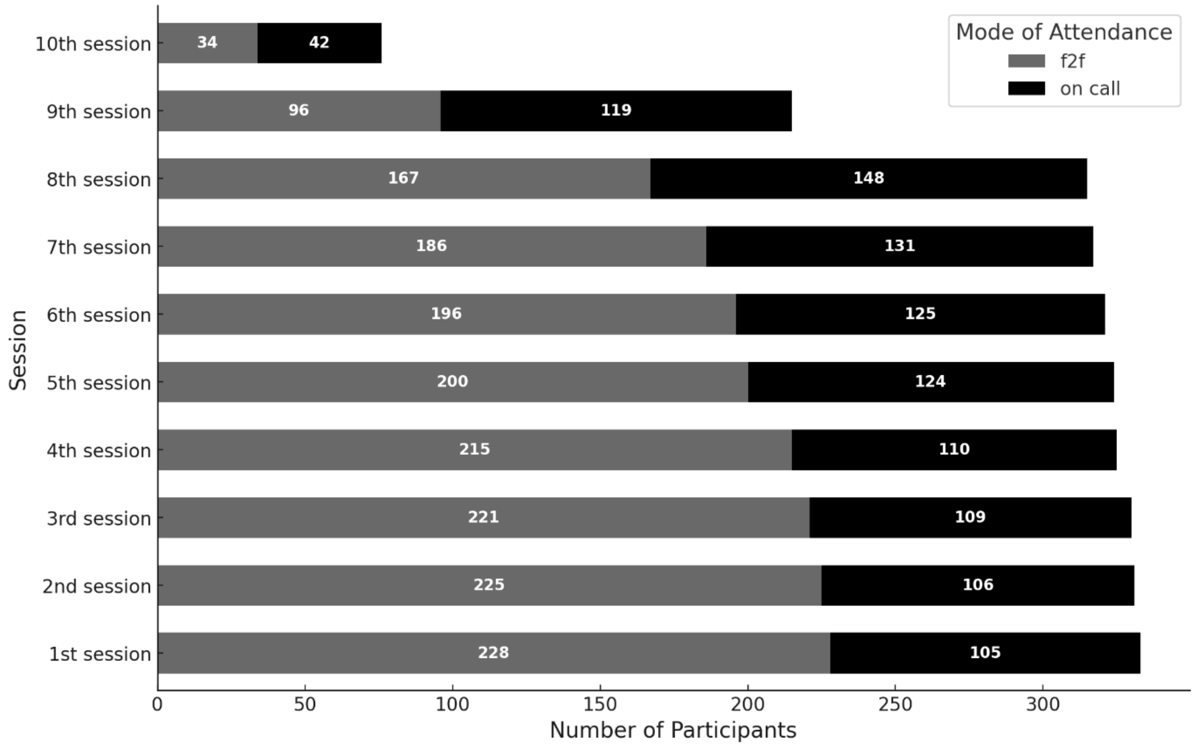

Supplement: Supplementary file 2 — Web appendix 2: Supplementary tables and figure [file husn083272.ww2.pdf]
